# Embedding Atomically Dispersed Manganese/Gadolinium Dual Sites in Oxygen Vacancy‐Enriched Biodegradable Bimetallic Silicate Nanoplatform for Potentiating Catalytic Therapy

**DOI:** 10.1002/advs.202307424

**Published:** 2023-11-30

**Authors:** Jin Ye, Kefen Zhang, Xing Yang, Mengting Liu, Yujie Cui, Yunlong Li, Chunsheng Li, Shuang Liu, Yong Lu, Zhiyong Zhang, Na Niu, Ligang Chen, Yujie Fu, Jiating Xu

**Affiliations:** ^1^ Key Laboratory of Forest Plant Ecology, Ministry of Education College of Chemistry, Chemical Engineering and Resource Utilization Northeast Forestry University Harbin 150001 P. R. China; ^2^ The Second Affiliated Hospital Heilongjiang Provincial Key Laboratory of Ecological Utilization of Forestry‐Based Active Substances Northeast Forestry University Harbin 150040 P. R. China; ^3^ Guangxi University of Science and Technology Liuzhou 545006 P. R. China; ^4^ School of Laboratory Medicine Wannan Medical College Wuhu Anhui 241002 P.R. China; ^5^ College of Biological Sciences and Technology Beijing Forestry University Beijing 100083 P.R. China

**Keywords:** biodegradation, dual active sites, multi‐catalytic therapy, oxygen vacancies, silicate single‐atom nanozymes

## Abstract

Due to their atomically dispersed active centers, single‐atom nanozymes (SAzymes) have unparalleled advantages in cancer catalytic therapy. Here, loaded with chlorin e6 (Ce6), a hydrothermally mass‐produced bimetallic silicate‐based nanoplatforms with atomically dispersed manganese/gadolinium (Mn/Gd) dual sites and oxygen vacancies (OVs) (PMn_SA_GMSNs‐V@Ce6) is constructed for tumor glutathione (GSH)‐triggered chemodynamic therapy (CDT) and O_2_‐alleviated photodynamic therapy. The band gaps of silica are significantly reduced from 2.78 to 1.88 eV by doping with metal ions, which enables it to be excited by a 650 nm laser to produce electron‐hole pairs, thereby facilitating the generation of reactive oxygen species. The Gd sites can modulate the local electrons of the atom‐catalyzed Mn sites, which contribute to the generation of superoxide and hydroxyl radicals (^•^OH). Tumor GSH‐triggered Mn^2+^ release can convert endogenous H_2_O_2_ to ^•^OH and realize GSH‐depletion‐enhanced CDT. Significantly, the hydrothermally generated OVs can not only capture Mn and Gd atoms to form atomic sites but also can elongate and weaken the O‐O bonds of H_2_O_2_, thereby improving the efficacy of Fenton reactions. The degraded Mn^2+^/Gd^3+^ ions can be used as tumor‐specific magnetic resonance imaging contrast agents. All the experimental results demonstrate the great potential of PMn_SA_GMSNs‐V@Ce6 as cancer theranostic agent.

## Graphical abstract

A biodegradable single‐atom nanozymes with Mn/Gd dual sites embedded in the oxygen vacancy‐enriched mesoporous bimetallic silicate nanospheres is massively produced by a facile hydrothermal method. After loading with chlorin e6 and PEGylation, the innovated nanopatform of PMn_SA_GMSNs‐V@Ce6 is used for achieving O_2_ self‐sufficient PDT, tumor GSH‐triggered CDT as well as tumor‐specific MRI.

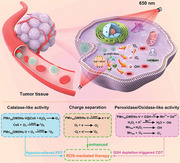

## 1 Introduction

With the development of nanotechnology, nanozymes with a tumor microenvironment (TME)‐specific catalytic nature have attracted considerable interest.^[^ ^1^ ^]^ Therefore, reactive oxygen species (ROS)‐mediated nanocatalytic strategies are considered promising cancer treatments.^[^ ^2^ ^]^ Generally speaking, ROS mainly include singlet oxygen (^1^O_2_), hydroxyl radicals (^•^OH), and superoxide anions radicals (^•^O_2_^−^), which are often employed to disrupt cell‐adaptation mechanisms and induce cell death.^[^ ^3^ ^]^ However, most reported nanozymes have fewer catalytic active sites and lower atomic utilization efficiency for enzyme‐like catalytic process, severely limiting their catalytic activity.^[^ ^4^ ^]^ Therefore, improving the atomic utilization of the nanozyme catalytic centers can be regarded as an effective strategy for enhancing the catalytic activity of nanozyme.

Recently, carbon‐based single‐atom nanozymes (SAzymes) by high‐temperature pyrolysis method have been increasingly attracting attention in tumor catalytic therapy owing the adjustability of the coordination environment, well‐defined electronic and geometric structures, maximum atomic utilization efficiency (100%), and unique quantum size effects.^[^ ^5^ ^]^ Shi et al. reported a bioinspired hollow N‐doped carbon sphere doped with a Cu‐based SAzymes that can directly catalyze the decomposition of both oxygen (O_2_) and H_2_O_2_ to ROS to suppress tumor growth.^[^ ^6^ ^]^ Meanwhile, SiO_2_ was introduced into PCN‐222 (Fe) to obtain high‐loading single‐atom catalysts to inhibit the agglomeration of Fe during pyrolysis.^[^ ^7^ ^]^ However, most of the reported carbon‐based SAzymes are challenging to degrade, metabolize, and remain in the body for a long time, which poses potential toxicity risks and greatly limits the use of SAzymes in cancer.^[^ ^8^ ^]^ Therefore, the development of biodegradable SAzymes is necessary.^[^ ^9^ ^]^ Compared to carbon‐based supports, silicon‐based supports are considered as ideal candidates owing to their unique structure, easy functionalization, and good biocompatibility. Benefiting from incorporation of transition metals (M = Mn, Fe, Cu, etc.) into the silsesquioxane framework (–Si–O–Si–), a metal silicate hybrid framework (–Si–O–M–) with additional functions, such as defect‐engineered biodegradability and photosensitizing ability, can be used for photodynamic therapy (PDT) application. Due to their merits of variable composition, low toxicity, and low price, silicate‐based materials have also been widely used in photocatalytic anticancer.^[^ ^10^ ^]^

PDT, which relies on external energy to convert O_2_ into ROS, is one of the most optimum anticancer therapies because of its minimal side effects, good therapeutic effect, and strong space‐time specificity.^[^ ^11^ ^]^ Due to its high singlet quantum yield, strong tissue penetration, high biocompatibility, and good PDT efficiency, Chlorin e6 (Ce6) has been widely employed for PDT.^[^ ^12^ ^]^ However, low ROS generation induced by hypoxia TME and quick energy attenuation often leads to limited PDT effects. To improve PDT outcomes, various nanozymes, including CaO_2_, MnO_2,_ and manganese silicate (MnSiO_3_), have been used to relieve tumor hypoxia, thereby achieving O_2_ self‐sufficient PDT.^[^ ^13^ ^]^ Huang et al. developed novel zinc silicate photocatalyst using a low‐temperature hydrothermal method, which endowed ZnSiO_4_ with high photocatalytic activity.^[^ ^14^ ^]^ Besides, Hyeon et al. designed a Ce6‐loaded manganese ferrite nanoparticle‐anchored mesoporous silica nanoparticles to enhance the therapeutic effects of PDT against hypoxic tumors.^[^ ^15^ ^]^ Meanwhile, Zhang et al. designed mesoporous copper/manganese silicate nanospheres that released O_2_ by decomposing endogenous H_2_O_2_ under hypoxic tumor conditions and further reacted with O_2_ to generate toxic ^1^O_2_ with 635 nm laser irradiation.^[13a]^ To overcome the limitations of single‐mode therapy, the PDT/chemodynamic therapy (CDT) combination therapy has been continuously explored to amplify tumor oxidative stress and achieve improved anticancer effects.^[^ ^1a^ ^,16]^ Dong et al. reported that an MnSiO_3_‐supported CaO_2_ nanoplatform achieved excellent CDT/PDT synergistic therapeutic effects.^[^ ^1^ ^]^ The Fenton‐like agent Mn^2+^ released from MnSiO_3_ can deplete ROS scavenger (GSH), further reducing ROS wastage and triggering GSH‐depletion‐enhanced CDT. Liu et al. successfully synthesized copper ferrite nanospheres as a nanodiagnostic and treatment platform for synergistic CDT/PDT therapy.^[^ ^17^ ^]^ However, the narrow light response range and high recombination efficiency of photogenerated electron‐hole pairs severely limit the practical application of silicates in photocatalytic anticancer applications.^[^ ^18^ ^]^

Defect engineering, especially that of oxygen vacancies (OVs), is considered an effective strategy for significantly boosting the photocatalytic activities of silicate nanomaterials.^[^ ^19^ ^]^ OVs can serve as electron‐ or hole‐trapping centers, significantly suppressing the recombination of photogenerated carriers.^[^ ^20^ ^]^ In addition, OVs can broaden the light‐absorption range of the catalysts. Li et al. reported Bi_2_O_3‐_ *_x_* possessing a substantial absorption range, attributing to the OVs‐induced local surface plasmon resonance effect.^[^ ^21^ ^]^ Additionally, many local electrons near the OVs can be transferred to the anoxic surface. Not only that, some small molecules including O_2_ and H_2_O_2_ can be activated and dissociated, generating more active substances.^[^ ^22^ ^]^ Wang et al. found that W_18_O_49_ with enriched surface OVs increased the adsorption energy of H_2_O_2_, indicating that the O–O bond of H_2_O_2_ was elongated and weakened owing to the stretching effect.^[^ ^23^ ^]^

In light of these ideas, herein, coupled with Ce6, OVs‐enriched PMn_SA_GMSNs‐V@Ce6 with atomically dispersed Mn/Gd dual sites was designed as TME‐responsive Fenton‐like reagent and dual‐photosensitizer for GSH‐triggered CDT, O_2_ self‐sufficient PDT, and magnetic resonance imaging (MRI). PMn_SA_GMSNs‐V continuously generated sufficient amounts of O_2_ as a photodynamic substrate by the endogenous H_2_O_2_. Meanwhile, PMn_SA_GMSNs‐V not only served as photosensitizer but also as a semiconductor. Upon 650 nm laser irradiation, PMn_SA_GMSNs‐V produced ^1^O_2_ via energy transfer. Of course, PMn_SA_GMSNs‐V can be excited by light to form photogenerated electrons and holes, thereby achieving multiple ROS. Subsequently, the photosensitizer molecule Ce6 was integrated into PMn_SA_GMSNs‐V, and PMn_SA_GMSNs‐V@Ce6 with dual photosensitizers displayed the high photodynamic efficiency and relieved hypoxia for enhanced PMn_SA_GMSNs‐V@Ce6‐mediated PDT. Significantly, atomic dispersion Mn active sites with 100% atomic utilization in PMn_SA_GMSNs‐V@Ce6 can directly trigger multiple enzyme activities. Because the –Mn–O– bonds are sensitive to TME, Fenton‐like Mn^2+^ degraded from the PMn_SA_GMSNs‐V@Ce6 may catalyze endogenous H_2_O_2_ to produce toxic ^•^OH, resulting in GSH‐depletion‐enhanced CDT. Particularly, the OVs in PMn_SA_GMSNs‐V@Ce6 promote the decomposition of H_2_O_2_ to achieve a large number of ^•^OH radicals. TME‐responsive *T*_1_‐weighted MRI nature of released Mn^2+^/Gd^3+^ and atomically dispersed Mn/Gd was employed to monitor the progress of multiple ROS‐mediated cancer treatments.

## 2 Results and Discussion

**Scheme 1:**
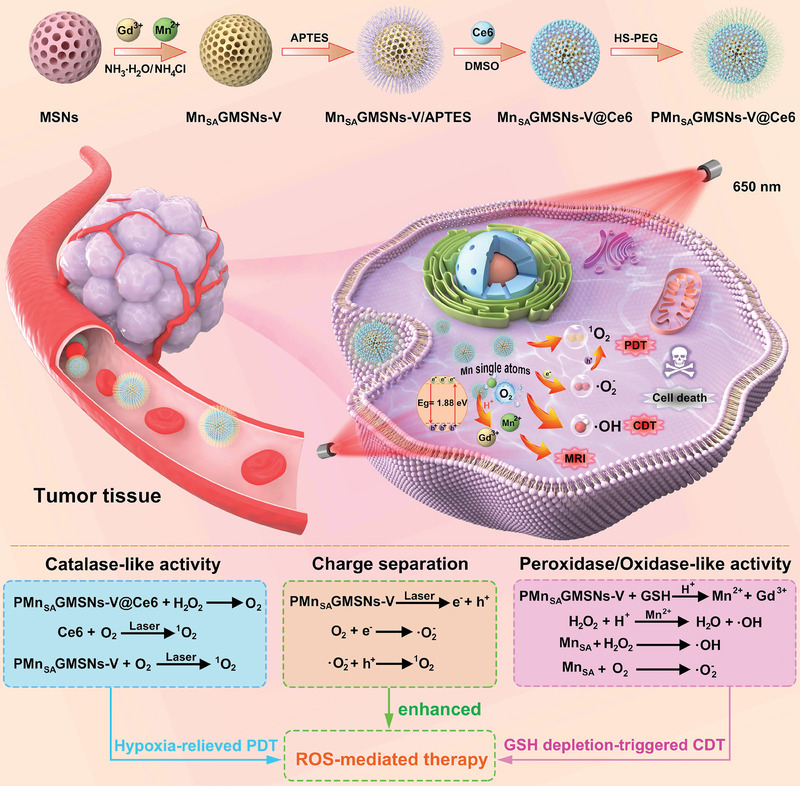
Schematic illustration of the synthesis of PMn_SA_GMSNs‐V@Ce6 and the theranostic mechanism of PMn_SA_GMSNs‐V@Ce6 for O_2_ self‐sufficient PDT, tumor GSH‐triggered CDT, and MRI under 650 nm laser irradiation.

The as‐obtained PMn_SA_GMSNs‐V@Ce6 with atomically dispersed Mn and Gd sites was synthesized via simple hydrothermal strategy using SiO_2_ nanospheres as template (**Scheme** **1**). In this nanosystem, PMn_SA_GMSNs‐V@Ce6 has some merits for potentiating cancer theranostics: i) PMn_SA_GMSNs‐V can serve as semiconductor to generate photogenerated carriers to produce multiple ROS under 650 nm laser irradiation (charge separation). ii) PMn_SA_GMSNs‐V@Ce6 continuously triggered catalase (CAT)‐like activity to generate sufficient amounts of O_2_ to continue PDT reaction. As a result, upon 650 nm laser irradiation, PMn_SA_GMSNs‐V@Ce6 can directly convert O_2_ into ^1^O_2_, exhibiting the hypoxia‐relieved PDT (catalase‐like activity). iii) Atomically dispersed Mn sites modulated by the Gd sites in PMn_SA_GMSNs‐V@Ce6 could trigger efficient peroxidase (POD)‐like and oxidase (OXD)‐like activities, endowing PMn_SA_GMSNs‐V@Ce6 with high enzymatic activity. Meanwhile, the Mn^2+^ release from the PMn_SA_GMSNs‐V@Ce6 due to reduced GSH can react with endogenous H_2_O_2_ to produce a large amount of ^•^OH, resulting in GSH‐depletion‐enhanced CDT effects. Importantly, the large number of OVs in PMn_SA_GMSNs‐V@Ce6 can elongate and weaken the O‐O bond of H_2_O_2_, which favors the decomposition of H_2_O_2_ to generate more ^•^OH. Collectively, PMn_SA_GMSNs‐V@Ce6 achieved high tumor inhibition based on the ROS‐mediated catalytic therapy. Additionally, Mn^2+^ and Gd^3+^ can be used as MRI contrast agent.

**Figure 1. a):**
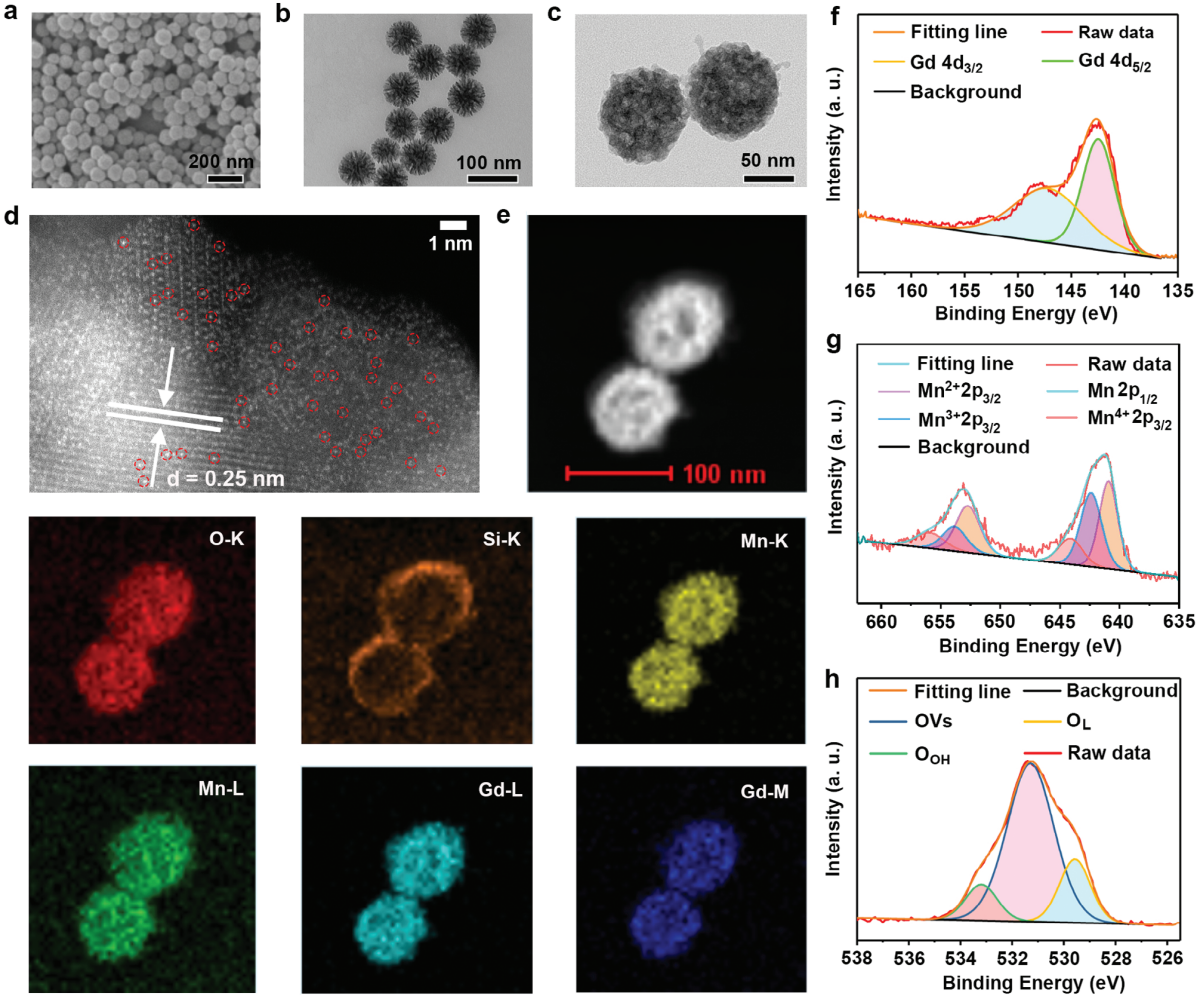
SEM image of MSNs, b) TEM images of MSNs and c) Mn_SA_GMSNs‐V, d) HAADF‐STEM image of Mn_SA_GMSNs‐V, e) corresponding TEM‐EDS mapping, and EDS of Mn_SA_GMSNs‐V. The high‐resolution XPS spectra of Mn_SA_GMSNs‐V for f) Gd 4d, g) Mn 2p, and h) O 1s.

The first step in fabricating SAzymes was successfully synthesizing monodisperse mesoporous silica nanoparticles (MSNs) via a microemulsion process. The images obtained using scanning electron microscopy (SEM) and transmission electron microscopy (TEM) revealed that the average size of MSNs was ≈80 nm, as illustrated in **Figure** **1**a,b. Especially, the MSNs exhibited the three‐dimensional central radiation dendritic structure. The porous characteristics and specific surface areas of MSNs were determined by the N_2_ adsorption‐desorption tests (Figure S1, Supporting Information). According to the Brunauer‐Emmett‐Teller (BET) fitting results, the MSNs possessed an average pore size of 3.4 nm and a specific surface area of 520.2 m^2^ g^−1^. The formation of crystalline phases for SiO_2_ was studied using the powder X‐ray diffraction (XRD) analysis. A prominent broad diffraction peak located at 20∼30° is observed in Figure S2, Supporting Information, which belongs to the typical diffraction peak of amorphous SiO_2_. When Mn ions were introduced into SiO_2_, silicate ions generated by MSNs in an alkaline environment can react with the manganese‐ammonium complex ions (Mn(NH_3_)_4_^2+^) to form MnSiO_3_ during the hydrothermal process. In Figure S3a, Supporting Information, the SEM images showed that MnSiO_3_ consists of uniform nanospheres with a mean size of ≈85 nm. The obtained MnSiO_3_ had a rough surface formed by accumulation of some granular nanoparticles. The corresponding X‐ray energy dispersive spectra (EDS) mapping of MnSiO_3_ revealed that only C, Si, O, and Mn elements were detected (Figure S3b, Supporting Information). From the TEM images, it is clear that MnSiO_3_ has a hollow structure, indicating more mesopores in MnSiO_3_ (Figure S3c,d, Supporting Information). As the core of SiO_2_ was constantly consumed, more basic MnSiO_3_ was produced by Mn(NH_3_)_4_^2+^, forming a hollow shell. High‐resolution transmission electron microscopy (HRTEM) shows a lattice spacing of 0.25 nm, corresponding to the typical lattice plane of MnSiO_3_.^[^ ^24^ ^]^ The element percentages of MnSiO_3_ were calculated to be C (41.39 wt.%), Si (14.67 wt.%), O (18.33 wt.%), and Mn (25.61 wt.%) (Figure S3e, Supporting Information). Moreover, the SEM, TEM images, and corresponding SEM‐EDS mapping of Gd/SiO_2_ are shown in Figure S4a‐d, Supporting Information. Compared to MnSiO_3_ with hollow structure, Gd/SiO_2_ exhibited agglomerated nanoparticles, indicating that MnSiO_3_ and Gd/SiO_2_ possess different morphology formation mechanisms. The existence of C (19.65 wt.%), Si (8.13 wt.%), O (69.91 wt.%), and Gd (2.32 wt.%) in Gd/SiO_2_ nanoparticles was verified by EDS mapping (Figure S4e, Supporting Information).

XRD patterns of MnSiO_3_ and Gd/SiO_2_ are displayed in Figure S5, Supporting Information. For MnSiO_3_, the diffraction peaks correspond well with the standard cards (JCPDS card 12–0181).^[13a]^ However, compared to standard Gd_2_O_3_ pattern (JCPDS card 88–2165), Gd/SiO_2_ showed poor crystallinity. This phenomenon can be explained by the fact that Mn ions can easily form Mn(NH_3_)_4_^2+^, indicating that Mn ions are more easily doped into SiO_2_ than Gd ions. When Mn and Gd ions were simultaneously integrated into SiO_2_, atomically dispersed Mn/Gd dual sites embedded in the mesoporous Gd and Mn co‐doped silicate nanospheres with OVs (Mn_SA_GMSNs‐V), as shown in Figure 1c and the corresponding size of Mn_SA_GMSNs‐V was about 85 nm. Subsequently, aberration‐corrected high‐angle annular dark‐field scanning TEM (HAADF‐STEM) was performed. In Figure 1d, many bright spots (circled in red) are attributed to the atomically dispersed Mn and Gd sites. The line‐scanning intensity profiles indicated the existence of an atomic dispersion of Gd (slightly brighter Gd dots and darker Mn dots) in Figure S6a‐b, Supporting Information. The corresponding atomic distance between Gd and Mn is ≈0.36 nm. Additionally, an obvious lattice fringe (0.25 nm) is observed, corresponding to the typical characteristic plane of MnSiO_3_. As shown in Figure S7, Supporting Information, a straightforward hydrothermal procedure may successfully manufacture Mn_SA_GMSNs‐V (0.995 g) on a large scale in a 300 mL reactor. Furthermore, the corresponding XRD pattern of Mn_SA_GMSNs‐V, which included distinctive peaks of both Gd_2_O_3_ and MnSiO_3_, confirmed the successful preparation of Gd/Mn co‐doped silicate. In Figure 1e, the TEM‐EDS mapping showed that Si, O, Mn, and Gd elements were evenly distributed throughout the Mn_SA_GMSNs‐V structure. Notably, Si was concentrated on the shell of the nanospheres rather than in the middle, suggesting that the internal SiO_2_ has been depleted. However, the morphology of Mn_SA_GMSNs‐V differed from MnSiO_3_, which was attributed to the presence of internal Gd_2_O_3_. According to EDS results (Figure S8, Supporting Information), the element contents of Si, O, Mn, and Gd are 10.29 wt.%, 39.33 wt.%, 16.58 wt.% and 33.80 wt.%, respectively.

Inductively coupled plasma (ICP) testing revealed the elemental content of Gd (34.90 wt.%) and Mn (17.00 wt.%) (Table S1) were consistent with EDS results. The surface elemental states and chemical compositions of the samples were examined using X‐ray photoelectron spectroscopy (XPS). The full‐scan spectra of Mn_SA_GMSNs‐V further confirm the successful introduction of Gd and Mn (Figure S9, Supporting Information). The high‐resolution XPS spectrum of Gd 4d is shown in Figure 1f. With a spin‐orbit splitting of 4.8 eV, the two prominent peaks at 142.4 and 147.2 eV are associated with the 4d_5/2_ and 4d_3/2_ energy levels of Gd, respectively.^[^ ^25^ ^]^ These findings indicate that the main valence of Gd was 3+. Meanwhile, the peaks located at 653.3 and 641.4 eV are attributed to the 2 p_1/2_ and 2 p_3/2_ of Mn, respectively. For Mn 2p_3/2_, the peak at 641.4 eV was divided into the three main peaks located 640.8, 642.0, and 644.8 eV, which correspond to Mn^2+^ (13.21%), Mn^3+^ (33.03%) and Mn^4+^ (16.20%), respectively.^[13a]^ The possibility of redox interaction with intratumoral GSH is significantly increased by the high‐valence Mn^3+^ and Mn^4+^, thereby improving the PDT and Fenton‐like effects (Figure 1g). Additionally, the high‐resolution XPS spectrum of O 1s was fitted in Figure 1h. The peaks situated at 529.5, 531.2, and 533.2 eV were ascribed to lattice oxygen (O_L_), OVs, and chemisorbed oxygen (O_c_), respectively.^[^ ^26^ ^]^

**Figure 2. a):**
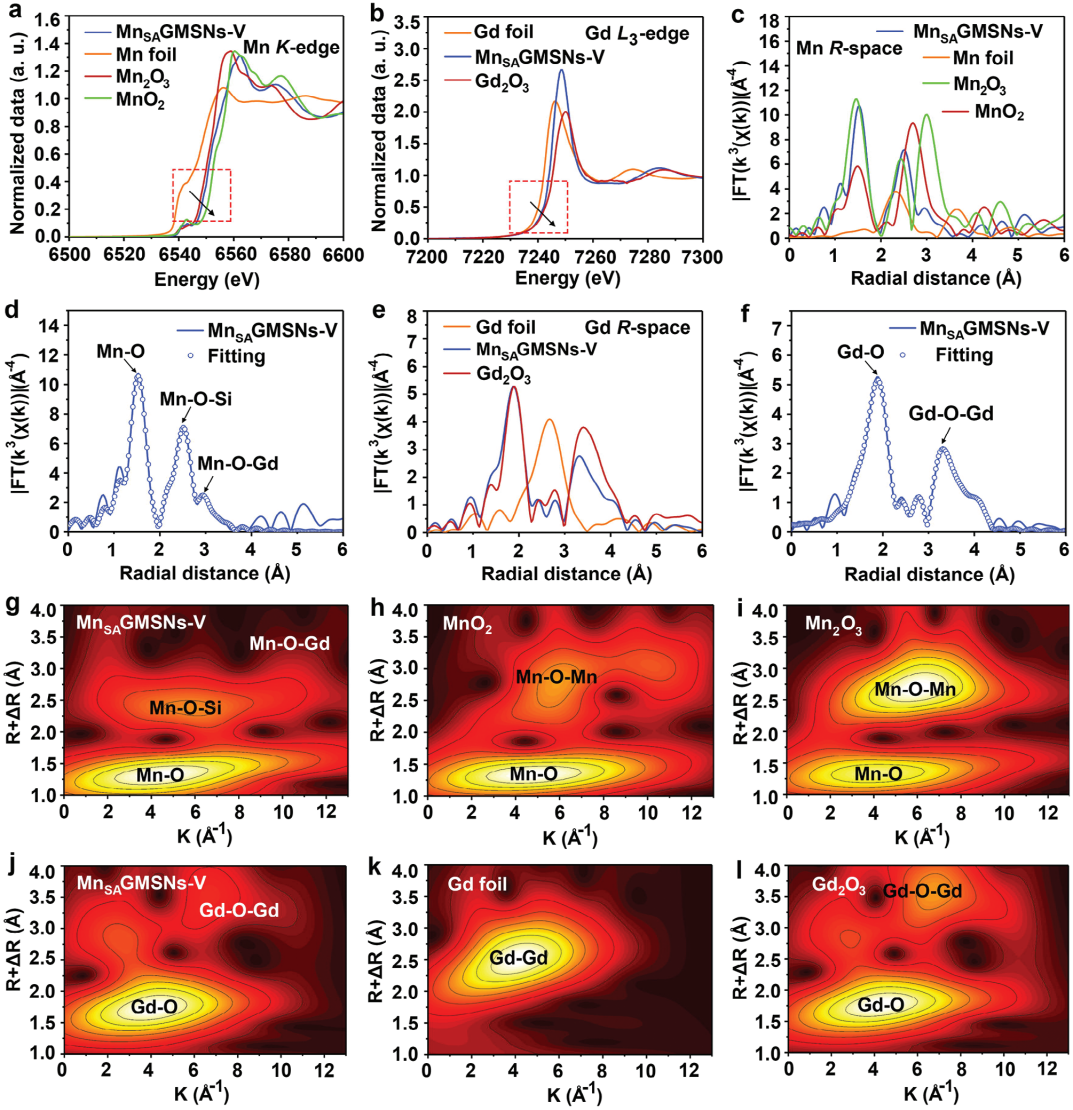
Normalized XANES of Mn K‐edge spectra of Mn foil, MnO_2_ Mn_2_O_3_, and Mn_SA_GMSNs‐V and b) Gd L_3_‐edge spectra of Gd foil, Gd_2_O_3_ and Mn_SA_GMSNs‐V. c) FT k^3^‐weighted Mn K‐edge EXAFS spectra of Mn foil, MnO_2_, Mn_2_O_3,_ and Mn_SA_GMSNs‐V and d) corresponding FT‐EXAFS fitting curves at R space of Mn. FT k^3^‐weighted Gd L_3_‐edge EXAFS spectra of Gd foil, Gd_2_O_3,_ and e) Mn_SA_GMSNs‐V and f) corresponding FT‐EXAFS fitting curves at R space of Gd. g‐l) WT‐EXAFS plots of Mn_SA_GMSNs‐V and corresponding reference samples.

To elucidate the chemical environment and coordination state of Mn and Gd species, Mn_SA_GMSNs‐V were investigated using X‐ray absorption near‐edge structure (XANES) and extended X‐ray absorption fine structure (EXAFS) spectroscopy. The Mn K‐edge and Gd L_3_‐edge for Mn_SA_GMSNs‐V, as well as their respective reference samples, were studied using normalized XANES curves. **Figure** **2**a demonstrates the near‐edge absorption of Mn_SA_GMSNs‐V, which is situated between Mn_2_O_3_ and MnO_2_, indicating that Mn atoms carried positive charges with the oxidation state, and the corresponding valence of Mn element was between +3 and +4, which is in agreement with XPS results.^[^ ^27^ ^]^ Similarly, the normalized XANES curves of the Gd L_3_‐edge for Mn_SA_GMSNs‐V are shown in Figure 2b. The absorption edge of the Mn_SA_GMSNs‐V lies between those of Gd foil and Gd_2_O_3_, demonstrating that the valence of Gd was between +0 and +3. Notably, the Gd L_3_‐edge absorption curve of Mn_SA_GMSNs‐V has a positive tendency to move in the direction of higher energy, and the Mn K‐edge absorption edge location was near to the lower energy direction, which supported the electron transfer from Gd to Mn in the XANES spectra.^[^ ^28^ ^]^ These results further revealed that the local electrons of Mn sites could be modulated by the introduction of Gd sites, thereby achieving a rapid catalytic reaction. In addition, the Fourier transformed (FT) k^3^‐weighted EXAFS (without phase correction) of Mn space in Mn_SA_GMSNs‐V were performed with reference samples (Figure 2c–d and Figure S10, Supporting Information). The FT‐EXAFS spectrum of the Mn_SA_GMSNs‐V displayed a predominant peak situated at 1.52 Å, corresponding to the Mn–O scattering path. There was no prominent contribution of metallic Mn‐Mn, revealing the isolation of Mn single atoms throughout the Mn_SA_GMSNs‐V samples, consistent with HAADF‐STEM results. Another peak at 2.51 Å belonged to the Mn–O–Si coordination for Mn_SA_GMSNs‐V, revealing the existence of MnSiO_3_, which follows the HAADF‐STEM results. It is important to note that a weaker peak situated at 2.94 Å could be associated with Mn‐O‐Gd coordination. Furthermore, the fitting curve in k spaces (Figure S11, Supporting Information) was employed to investigate the structural parameters of reference samples and Mn_SA_GMSNs‐V.

The corresponding curve‐fitting results were in excellent agreement with the experimental data. Similarly, the k^3^‐weighted FT‐EXAFS was recorded at Gd L_3_‐edge of the Mn_SA_GMSNs‐V with Gd foil and Gd_2_O_3_ (Figure 2e). The k^3^‐weighted FT‐EXAFS spectroscopy clarified the coordination surrounding of Gd. In Figure 2f, the two major peaks at about 1.88 and 3.29 Å for Mn_SA_GMSNs‐V corresponded to the Gd‐O and Gd‐O‐Gd coordination peak, respectively. Compared with Gd foil and Gd_2_O_3_ (Figure S12, Supporting Information), the Gd‐Gd coordination peak was not detected in Mn_SA_GMSNs‐V, indicating that metallic Gd did not aggregate. Figure S13, Supporting Information, illustrates the results of the EXAFS curve fitting of the Mn_SA_GMSNs‐V, Gd foil, and Gd_2_O_3_ in k spaces to determine the coordination arrangement of Gd, which suggested the experimental data and the results of curve fitting were quite similar. Wavelet transform (WT) analysis with a higher resolution was used to determine the roles of different scattering paths. In Figure S14, Supporting Information, Mn foil has a predominant intensity maximum at 6.2 Å^−1^, which was attributed to metallic Mn‐Mn coordination. As displayed in Figure 2g‐i, WT contour plots of MnO_2_, Mn_2_O_3,_ and Mn_SA_GMSNs‐V were obtained. For Mn_SA_GMSNs‐V, three obvious intensity maximum at 4.4, 5.8, and 10.6 Å^−1^ was observed, corresponding to the Mn–O, Mn–O–Si, and Mn–O–Gd, respectively. Moreover, compared to the Gd foil and Gd_2_O_3_, the WT contour plots of Mn_SA_GMSNs‐V showed the similar characteristic maximum intensity located at 4.06 and 6.7 Å^−1^ (Figure 2j,l) and no obvious Gd–Gd coordination peak was detected, indicating the existence of Gd_2_O_3_ in Mn_SA_GMSNs‐V. The Mn–O and Gd–O average coordination numbers were determined to be 3.7 and 5.5 based on the EXAFS fitting parameters, respectively, which revealed that Mn sites were bonded with four atoms in the structure of Mn_SA_GMSNs‐V (Table S2). These data can further verify the successful doping of Gd and Mn, consistent with TEM mapping results. Taken all together, the coordination environment of the Mn element proves that some Mn atoms mainly exist in atomic dispersion in Mn_SA_GMSNs‐V due to the capture of OVs, and Mn atoms do not agglomerate. However, according to the results of HAADF‐STEM and EXAFS, Gd was primarily composed of Gd_2_O_3_ and a small amount of atomic dispersion of Gd.

**Figure 3. a):**
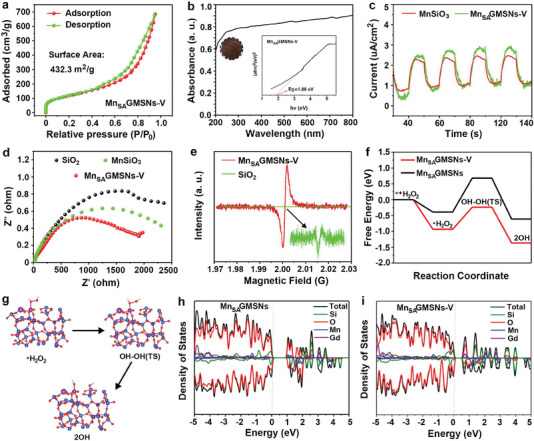
N_2_ adsorption‐desorption isotherms of Mn_SA_GMSNs‐V, b) UV‐vis diffuse absorbance spectra of Mn_SA_GMSNs‐V (insert: the corresponding plots of (αhν)*^n^* ^/2^ for Mn_SA_GMSNs‐V). c) Photocurrent transient responses, d) electrochemical impedance spectroscopy. e) The ESR spectra of pure SiO_2_ and Mn_SA_GMSNs‐V. The free energy diagrams of Mn_SA_GMSNs‐V and f,g) Mn_SA_GMSNs during a catalytic process in an acidic environment. h) DOS of Mn_SA_GMSNs and i) Mn_SA_GMSNs‐V.

In **Figure** **3**a, the N_2_ adsorption‐desorption isotherms exhibited an obvious hysteresis loop, indicating that Mn_SA_GMSNs‐V possessed many abundant mesoporous structures.^[^ ^24^ ^]^ The Brunauer–Emmett–Teller (BET) surface area and average pore size were calculated to be 432.3 m^2^ g^−1^ and 5.8 nm (Figure S15, Supporting Information), respectively. Compared to the pure SiO_2_, BET value of Mn_SA_GMSNs‐V significantly decreased, which was attributed to the deduction of pore. Ultraviolet‐visible diffuse reflectance spectra (UV‐vis DRS) was employed to determine the light‐absorption properties of the synthesized nanomaterials. The UV‐vis DRS spectrum of pure SiO_2_ is displayed in Figure S16a, and corresponding band gap was calculated to be 2.78 eV (Figure S16b) according to Kubelka‐Munk Equation (1),^[^ ^29^ ^]^ indicating lower light absorption of SiO_2_.

(1) $$\alpha hv=k{\left(hv-Eg\right)}^{n/2}$$

The band gaps of Gd/SiO_2_ (2.63 eV) (Figure S16c,d, Supporting Information) and MnSiO_3_ (2.12 eV) (Figure S16e,f, Supporting Information) are lower than those of pure SiO_2_. However, as Gd and Mn were co‐doped into SiO_2_, the band gap of Mn_SA_GMSNs‐V (1.88 eV) is shown in Figure 3b, indicating that Mn_SA_GMSNs‐V showed a broad absorption range and could be stimulated using a 650 nm laser (*E* = 1.91 eV) for PDT.

Separation efficiency of charge carriers was further revealed by photoluminescence (PL) emission spectroscopy and photoelectrochemical characterizations. Generally, a lower emission intensity is caused by a lower efficiency radiative recombination.^[^ ^30^ ^]^ Figure S17, Supporting Information, shows the separation efficiency of charge carriers for MnSiO_3_, Mn_SA_GMSNs‐V, and Mn_SA_GMSNs‐V@Ce6. Obviously, MnSiO_3_ showed the strongest fluorescence signal among all materials, revealing that MnSiO_3_ has the highest recombination rate of carriers. For Mn_SA_GMSNs‐V, the lower fluorescence intensity was observed due to the doping of Gd element. By contrast, Mn_SA_GMSNs‐V@Ce6 exhibited the lowest fluorescence intensity due to the quenching of molecule aggregation.

Moreover, the charge transfer and separation efficiencies over MnSiO_3_ and Mn_SA_GMSNs‐V@Ce6 were investigated. A weaker intensity is consistent with lower separation efficiency. As shown in Figure 3c, Mn_SA_GMSNs‐V displayed the highest photocurrent value than MnSiO_3_, indicating that more photogenerated electrons were generated under light irradiation. Electrochemical impedance spectroscopy (EIS) was conducted to study the charge‐transfer performance (Figure 3d). The semicircle of pure SiO_2_ is the largest, indicating that its resistance is the largest. When Mn ions were doped into SiO_2_, MnSiO_3_ exhibited the smaller semicircle than SiO_2_ due to its lower resistance. Among these samples, Mn_SA_GMSNs‐V had the lowest semicircle, indicating Mn_SA_GMSNs‐V possessed the lowest resistance and highest efficiency of electron‐hole transfer by the introduction of Gd sites, which can accelerate the catalytic reaction.^[^ ^31^ ^]^ The time‐resolved transient PL (TRPL) decays of pure SiO_2_ and Mn_SA_GMSNs‐V were fitted to reveal the specific charge carrier dynamics (Figure S18, Supporting Information). The average lifetimes of pure SiO_2_ and Mn_SA_GMSNs‐V were calculated to be 5.27 and 4.73 ns, respectively. Compared to the SiO_2_, when Gd and Mn ions were doped and integrated into SiO_2_, Mn_SA_GMSNs‐V displayed a shorter average lifetime, suggesting Mn_SA_GMSNs‐V had the highest carrier separation efficacy.^[19a]^ Furthermore, linear sweep voltammetry (LSV) was employed to study the adsorption capacity of different materials, including SiO_2_, MnSiO_3_, and Mn_SA_GMSNs‐V for H_2_O_2_ substrate (Figure S19, Supporting Information). In the presence of H_2_O_2_, pure SiO_2_ displayed poor current density under weakly acidic conditions. Compared to SiO_2_, an obvious current density for MnSiO_3_ was observed, indicating that MnSiO_3_ showed better affinity under the same conditions. To our delight, Mn_SA_GMSNs‐V possessed the highest current density along with doping of Gd and Mn, suggesting Mn_SA_GMSNs‐V exhibited the best affinity for H_2_O_2_. Electron spin resonance (ESR) can be regarded as powerful evidence to verify the presence of OVs in Mn_SA_GMSNs‐V. As shown in Figure 3e and Figure S20, Supporting Information, pure SiO_2_ showed a weaker Lorentz line due to the presence of a small amount of unpaired electrons, suggesting the existence of OVs. However, the SiO_2_ after hydrothermal treatment showed a slightly stronger signal. Compared to pure SiO_2_ and SiO_2_ after hydrothermal treatment, a stronger electron paramagnetic resonance signal for Mn_SA_GMSNs‐V (*g* = 2.001) was observed, indicating the number of unpaired electrons was markedly increased. This phenomenon reveals that many unpaired electron‐localized OVs can anchor redundant Mn atoms that are not converted into MnSiO_3_. That is, the Mn atoms can occupy OVs sites.^[^ ^32^ ^]^

OVs with many local electrons can activate or dissolve some small molecules, such as O_2_ and H_2_O_2_, and thus positively affect the catalytic process. Subsequently, density functional theory (DFT) computations were performed to determine the critical effects of OVs. The adsorption capacity of H_2_O_2_ for Mn_SA_GMSNs‐V without or with OVs at different active sites was studied. As shown in Figure S21a‐d, Supporting Information, compared to the Gd sites without OVs (*E*_ads_ = −0.639 eV), lower adsorption energy (*E*_ads_ = −1.163 eV) was calculated for Gd sites with OVs. However, when Mn was used as the active site, Mn sites with OVs had lower adsorption energy (*E*_ads_ = −1.832 eV) than Mn sites without OVs (*E*_ads_ = −1.054 eV), which verified that OVs favored the adsorption for H_2_O_2_. In addition, the adsorption energy of Mn sites with OVs was lower than Gd sites with OVs, indicating that the adsorption process was more likely to occur at the Mn sites.

The existence of OVs might elongate and weak the O–O bond of H_2_O_2_, which may accelerate H_2_O_2_ breakdown. Importantly, the free energy diagrams of Mn_SA_GMSNs‐V and Mn_SA_GMSNs were simulated during weaker acidic conditions (Figure 3f,g). The simulated catalytic mechanism of Mn_SA_GMSNs‐V under acidic conditions could be divided into the following processes. Initially, activated *H_2_O_2_ may be produced by simply absorbing H_2_O_2_ molecules onto the Mn sites of Mn_SA_GMSNs‐V. Compared to Mn_SA_GMSNs (Δ*G* = −0.39 135 eV), the lower free energy for Mn_SA_GMSNs‐V (Δ*G* = −0.93 542 eV) was observed, indicating Mn_SA_GMSNs‐V was easy to combine with H_2_O_2_ molecules. Secondly, hydroxyl groups (OH*) bonded to the Mn sites, and reactive ^•^OH were generated by uniformly cleaving the activated H_2_O_2_ molecules. Then, ^•^OH could be generated under the stage. However, compared to the Mn_SA_GMSNs, the free energy barrier in the rate‐determining step of the ^•^OH formation for Mn_SA_GMSNs‐V was much negative (−1.367 versus −0.613 eV), indicating Mn_SA_GMSNs‐V is more beneficial for the formation of ^•^OH because of the existence of OVs.

In addition, the density of states (DOS) for Mn_SA_GMSNs and Mn_SA_GMSNs‐V was employed to elucidate the catalytic reaction mechanisms. As illustrated in Figure 3h–i, the strongest interactions between Mn_SA_GMSNs‐V and H_2_O_2_ were inferred by the larger DOS around the Fermi level, and several novel hybridized electronic states were detected for Mn_SA_GMSNs‐V. Subsequently, titanium oxide sulfate (TiOSO_4_) was used to detect changes in the concentration of H_2_O_2_ solution treated with PMn_SA_GMSNs‐V at different times. In Figure S22, Supporting Information, the UV‐Vis absorption peak of TiOSO_4_ solution gradually decreased, indicating that H_2_O_2_ was consumed along with the extension of time. To verify CAT‐like activity of Mn_SA_GMSNs‐V, their catalytic activity of Mn_SA_GMSNs‐V on H_2_O_2_ decomposition was investigated. The corresponding curves of O_2_ concentration change in the different solution were also obtained. As shown in Figure S23, Supporting Information, when H_2_O_2_ was added into Mn_SA_GMSNs‐V, an obvious real‐time increase in O_2_ concentration was observed than SiO_2_ and MnSiO_3_. Almost no oxygen bubbles were observed in the pure SiO_2_ and H_2_O_2_ solution (Video S1). Compared to pure SiO_2_ and H_2_O_2_ solution, a small amount of oxygen bubbles were observed in pure MnSiO_3_ solution. When Mn_SA_GMSNs‐V was added into H_2_O_2_ solution, an obvious generation of oxygen bubbles was observed owing to the introduction of Gd sites, indicating that Mn_SA_GMSNs‐V can facilitate the breakdown of H_2_O_2_ into O_2_, confirming the CAT‐like activity of Mn_SA_GMSNs‐V (2):

(2) $${\mathrm{PMn}}_{SA}\mathrm{GMSNs}-V+H_{2}O_{2}\to O_{2}$$

In order to further verify CAT enzyme activity of the PMn_SA_GMSNs‐V@Ce6, a hypoxia‐inducible factor‐1 (HIF‐1α) protein was employed to assess the degree of cell hypoxia. Compared with control group, western blot analysis of PMn_SA_GMSNs‐V@Ce6 showed that the signal intensity of HIF‐1α decreased (Figure S24, Supporting Information). These results can suggest that PMn_SA_GMSNs‐V@Ce6 could alleviate hypoxia via O_2_ generation following cellular uptake.

**Figure 4. a):**
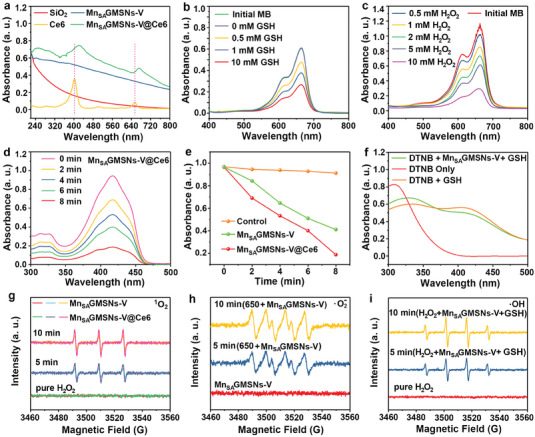
UV‐vis absorption spectra of SiO_2_, Mn_SA_GMSNs‐V, Ce6, Mn_SA_GMSNs‐V@Ce6, MB degradation by ^•^OH generated by different concentrations of GSH‐treated b) Mn_SA_GMSNs‐V@Ce6 (1 mg mL^−1^) and c) H_2_O_2_ (8 mM). d) Time‐dependent absorption spectra and e) the corresponding retention rate of DPBF in the presence of Mn_SA_GMSNs‐V and Mn_SA_GMSNs‐V@Ce6 under 650 nm laser irradiation. GSH depleting abilities using DTNB as f) the trapping agent of sulphydryl (‐SH) in GSH. ESR spectra for detection of ^1^O_2_, ^•^O_2_^−^ and ^•^OH versus magnetic field in different conditions for Mn_SA_GMSNs‐V and Mn_SA_GMSNs‐V@Ce6 (g‐i).

To confirm that Ce6 was successfully loaded in Mn_SA_GMSNs‐V, the UV‐vis absorption spectra of all prepared nanozymes were investigated. As shown in **Figure** **4**a, Mn_SA_GMSNs‐V exhibited the wide absorption than pure SiO_2_ in the visible light range. For Ce6, two absorption peaks were observed at 402 and 655 nm, which correspond to the typical peaks of Ce6. Moreover, Mn_SA_GMSNs‐V@Ce6 demonstrated characteristic peaks similar to those of Ce6 at 422 nm and 672 nm, indicating the successful loading of Ce6.^[^ ^12^ ^]^ Notably, an obvious red‐shift for Mn_SA_GMSNs‐V@Ce6 was observed due to the insertion of metal ion into the framework of Ce6.^[^ ^33^ ^]^ Besides, the loading amount of Ce6 was calculated based on the UV‐Vis absorption spectra of Ce6 at varying concentrations (Figure S25a) and the standard curve for Ce6 (Figure S25b, Supporting Information). As a result, Ce6 loading efficiency in the Mn_SA_GMSNs‐V@Ce6 was calculated to be 24.7 wt.% (Figure S25c, Supporting Information). Subsequently, the response capacity of Mn_SA_GMSNs‐V to different concentrations of GSH and H_2_O_2_ was investigated. As displayed in Figure 4b, under mild acid conditions, the absorbance value of MB obviously decreased at 664 nm as the GSH concentration increased from 0 to 10 mM, demonstrating a concentration‐dependent relationship between the absorbance and GSH concentrations. These results further revealed that the weakly acid environment (pH 6.5) and reduced GSH promoted rapid biodegradation of Mn_SA_GMSNs‐V to coincidentally trigger a Fenton‐like reaction, which was attributed to the response of Mn–O bonds.^[^ ^27^ ^]^ Similarly, the absorbance of MB also decreased as the concentration of H_2_O_2_ increased due to more ROS generation (Figure 4c).

Moreover, 1,3‐diphenylisobenzofuran (DPBF), with a maximum absorbance peak located at 420 nm, was employed to verify the generation of ^1^O_2_.^[^ ^34^ ^]^ Figure S26, Supporting Information, shows absorption spectrum of DPBF at different times in the presence of Mn_SA_GMSNs‐V by 650 nm laser irradiation, indicating Mn_SA_GMSNs‐V can act as photosensitizer (3). When Ce6 was integrated into Mn_SA_GMSNs‐V, a significant decrease in the absorption spectra of DPBF over Mn_SA_GMSNs‐V@Ce6 was observed (Figure 4d). Compared to the Mn_SA_GMSNs‐V, Mn_SA_GMSNs‐V@Ce6 displayed lower absorbance in DPBF absorption spectra (Figure 4e), which was attributed to the synergistic effects of Mn_SA_GMSNs‐V and Ce6 (4).

(3)

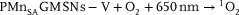



(4)

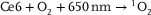




Meanwhile, 650 nm laser illumination (0.6 W cm^−2^), Mn_SA_GMSNs‐V and Mn_SA_GMSNs‐V@Ce6 for ROS generation were investigated using singlet oxygen sensor green (SOSG) as a detector. When ^1^O_2_ reacted with SOSG, a fluorescence emission signal was detected at 526 nm.^[2b]^ Figure S27, Supporting Information, showed that there was no detectable fluorescence emission signal from H_2_O_2_, suggesting H_2_O_2_ basically does not generate ^1^O_2_. For Mn_SA_GMSNs‐V, lower emission intensity was observed, confirming that Mn_SA_GMSNs‐V can serve as a photosensitizer, thereby transforming triplet oxygen (^3^O_2_) to ^1^O_2._ Furthermore, Mn_SA_GMSNs‐V@Ce6 displayed the strongest fluorescence intensity, indicating excellent performance toward the ^1^O_2_ generation due to the synergistic effects of Mn_SA_GMSNs‐V and Ce6.

Additionally, 5, 5′‐dithio‐bis (2‐nitrobenzoic acid) (DTNB) was employed to evaluate the depletion ability of GSH. It is reported that DTNB can react with GSH to produce yellow chromogenic products with a prominent absorbance peak located at 412 nm.^[^ ^35^ ^]^ For pure DTNB, there was no detectable absorption signal at 412 nm in the UV‐vis spectra (Figure 4f). Interestingly, an obvious absorption peak was detected, which was attributed to the color reaction of GSH and DTNB. When Mn_SA_GMSNs‐V was added to the GSH solution, a lower absorbance was observed in the UV absorption spectra, implying Mn_SA_GMSNs‐V can consume GSH. The GSH degradation rate in HeLa cells treated with varying concentrations of PMn_SA_GMSNs‐V is shown in Figure S28, Supporting Information. The intracellular GSH levels showed a concentration‐dependent decline after treatment with PMn_SA_GMSNs‐V. As a result, GSH‐responsive behavior can effectively prevent the ROS loss, thereby realizing GSH‐depletion‐enhanced CDT.

The low‐temperature ESR spectra were collected using 2,2,6,6‐tetramethylpiperidine (TEMP) and 5,5‐dimethyl‐1‐pyrroline N‐oxide (DMPO) as spin‐trapping agents to identify ROS production.^[^ ^36^ ^]^ As shown in Figure 4g, both Mn_SA_GMSNs‐V and Mn_SA_GMSNs‐V@Ce6 presented a straight line in the presence of only pure H_2_O_2_, indicating ^1^O_2_ cannot be produced. However, when Mn_SA_GMSNs‐V were illuminated by a 650 nm laser (5 min), there was a significant signal for ^1^O_2_ with a strength ratio of 1:1:1, revealing Mn_SA_GMSNs‐V can be regarded as photosensitizer, which is following with SOSG results.^[^ ^37^ ^]^ Stronger signals were observed as the illumination time increased (10 min). Compared to the Mn_SA_GMSNs‐V, Mn_SA_GMSNs‐V@Ce6 displayed strongest ^1^O_2_ signals under the same time conditions, which was attributed to the synergistic effects of Mn_SA_GMSNs‐V and Ce6. The Mott‐Schottky (MS) plots were used to examine the conduction band (CB) of Mn_SA_GMSNs‐V to explore the ROS‐generating process better. Figure S29, Supporting Information, shows that the slope of the MS plot was unequivocally positive, indicating that Mn_SA_GMSNs‐V was n‐type semiconductors.^[^ ^38^ ^]^ As a matter of fact, the location of the CB is close to the flat potential of the n‐type semiconductors.^[^ ^39^ ^]^ It was discovered that the flat potential of Mn_SA_GMSNs‐V was −0.75 eV (vs. Ag/AgCl at pH 7). The calculated value for the matching CB of Mn_SA_GMSNs‐V was about −0.55 eV (vs. Normal Hydrogen Electrode (NHE)) based on Equation (5). Meanwhile, the VB of Mn_SA_GMSNs‐V was evaluated by XPS valence band spectrum (Figure S30), consistent with MS results. Based on Equation (6), the VB of Mn_SA_GMSNs‐V was calculated to be +1.33 eV.

(5) $$E_{\mathrm{NHE}}=E_{\mathrm{Ag}/\mathrm{AgCl}}+0.197$$

(6) $$E_{\mathrm{CB}}=E_{\mathrm{VB}}-E_{g}$$

When the Mn_SA_GMSNs‐V was illuminated by 650 nm laser (> 1.88 eV), electrons originating from the VB of Mn_SA_GMSNs‐V were able to transition to the corresponding CB to generate electron‐hole pairs (7). The VB energy of Mn_SA_GMSNs‐V (+1.33 eV) was lower than that of E^θ^(^•^OH/H_2_O) (+1.99 eV), indicating ^•^OH could not be produced in this manner.^[^ ^40^ ^]^ Nevertheless, the CB potential (−0.55 eV) of Mn_SA_GMSNs‐V is more negative than that of E^θ^ (O_2_/^•^O_2_^−^) (−0.33 eV), suggesting the formation of an active species (^•^O_2_^−^) might be formed.^[^ ^41^ ^]^ In addition, the ESR spectra (Figure 4h) further reveal the generation of ^•^O_2_^−^, which was attributed to the fact that the photogenerated electrons were further captured by O_2_ (8).

(7) $$\begin{array}{ccc} & & {\mathrm{PMn}}_{\mathrm{SA}}\mathrm{GMSNs}-V+650\;\mathrm{nm}\to {\mathrm{PMn}}_{\mathrm{SA}}\mathrm{GMSNs}-V\left(e^{-}\right)\\ & & \;+{\mathrm{PMn}}_{\mathrm{SA}}\mathrm{GMSNs}-V\left(h^{+}\right) \end{array}$$

(8)

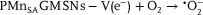




Furthermore, the ESR spectra revealed that pure Mn_SA_GMSNs‐V had no ^•^O_2_^−^ without 650 nm laser irradiation. In contrast, when Mn_SA_GMSNs‐V were excited by 650 nm laser irradiation, a distinguishable characteristic peak that has an intensity ratio of 1:1:1:1 was detected, suggesting the presence of ^•^O_2_^−^. Importantly, Mn_SA_GMSNs‐V possessed the strongest ^•^O_2_^−^ signal after 10 min of illumination, revealing more electron‐hole pairs could induce and generate more ROS. Remarkably, the typical ESR signal of ^•^O_2_^−^ were detected not only under 650 nm laser irradiation but also non‐light conditions (only in the presence of H_2_O_2_) (Figure S31, Supporting Information), indicating the existence of atomically dispersed Mn sites in the Mn_SA_GMSNs‐V, thereby achieving excellent OXD‐like activity. Similarly, a free‐radical trapping experiment further verified the presence of Mn single atom (Figure S32, Supporting Information). Moreover, the production of ^•^OH was confirmed by ESR assays. As shown in Figure 4i, pure H_2_O_2_ presented a straight line and hardly produced ^•^OH. After GSH and Mn_SA_GMSNs‐V were introduced in H_2_O_2_ solution (5 min), the ESR spectrum exhibited a characteristic 1:2:2:1 ^•^OH signal peaks due to the generation of ^•^OH. Notably, more ^•^OH was detected with increased reaction time (10 min). More importantly, the signal of ^•^OH for Mn_SA_GMSNs‐V was also detected only in the presence of H_2_O_2_ (Figure S33, Supporting Information). This phenomenon can be attributed to the atomically dispersed Mn in the Mn_SA_GMSNs‐V, thereby inducing more ^•^OH generation. Besides, compared to the absence of GSH, Mn_SA_GMSNs‐V showed enhanced ^•^OH signal in the presence of GSH due to the release more Fenton‐like ions.

Mn_SA_GMSNs‐V were endowed with degradable property due to the response of Mn–O bonds in Mn_SA_GMSNs‐V. At first, the accumulated releasing of Mn and Gd ions from MnSiO_3_ and Gd/SiO_2_ was evaluated. As show in Figure S34, Supporting Information, the accumulated releasing rate of Mn from pure MnSiO_3_ was faster, indicated that MnSiO_3_ exhibited an obvious biodegradation ability compared to that of Gd/SiO_2_. Subsequently, the biodegradation behavior of Mn_SA_GMSNs‐V in the simulated TME was directly observed by SEM and TEM analysis. The pictures of Mn_SA_GMSNs‐V treated with different solutions for 24 h were shown in Figure S35a‐b, Supporting Information. It was obvious that reductive GSH and acid conditions have a significant effect, thereby accelerating the biodegradation behavior of Mn_SA_GMSNs‐V to achieve the enhanced CDT (9).

(9)

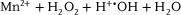




As shown in Figure S36a, Supporting Information, Mn_SA_GMSNs‐V had a completely spherical nanostructure under neutral condition (pH 7.4) without GSH, whereas the majority of Mn_SA_GMSNs‐V significantly collapsed under acidic conditions (pH 6.5) and in the presence of GSH (10 mM) after 24 h. Moreover, ICP‐OES was used to analyze the Mn and Gd ions liberated from Mn_SA_GMSNs‐V after treatment with or without GSH under various pH conditions (Figure S36b,c, Supporting Information). As anticipated, under the mildly acidic conditions, reduced GSH levels greatly stimulated Mn_SA_GMSNs‐V biodegradation to release Mn and Gd. In the simulated environment, the cumulative degradation rate of Mn was much higher than that of Gd, because the Mn–O bond was more sensitive to the tumor environment. Due to the cleavage of the Mn–O bond, the release of Mn ions originating from the Mn_SA_GMSNs‐V resulted in a large number of defects in the skeleton under acidic conditions and GSH, which could further induce the break of the Gd–O bonds.

Benefiting from the biodegradation of Mn and Gd, the MRI capabilities of Mn_SA_GMSNs‐V were examined under simulated normal and tumor conditions. As shown in Figure S36d, Supporting Information, the *T*_1_ signal exhibited increasing brightness of phantom picture in all groups when Mn^2+^ and Gd^3+^ concentration were increased from 0 to 1.6 mM. The enhancements in brightness with reductive GSH (10 mM) and weakly acidic conditions (pH 6.5) exhibited an extraordinarily higher degradation rate than that in the neutral (pH 7.4) and the absence of GSH conditions (0 mM). The corresponding initial longitudinal relaxation rate (r_1_) of Mn_SA_GMSNs‐V in normal condition or weakly acid condition without or with GSH was evaluated to be 1.14 and 6.11 mM^−1^ s^−1^, respectively (Figure S36e, Supporting Information), indicating the remarkably improved release of Mn^2+^ and Gd^3+^ from Mn_SA_GMSNs‐V under acid reduced environment, thereby confirming the enhancement of TME‐responsive MRI of Mn_SA_GMSNs‐V (10).

(10) $${\mathrm{PMn}}_{\mathrm{SA}}\mathrm{GMSNs}-V+\mathrm{GSH}/H^{+}\to {\mathrm{Gd}}^{3+}+{\mathrm{Mn}}^{2+}+\mathrm{GSSG}$$

Biological transmission electron microscopy (bio‐TEM) was used to observe intracellular biodegradation of PMn_SA_GMSNs‐V. When the incubation time of PMn_SA_GMSNs‐V was only 0.5 h, in addition to the observation that part of the shell remained almost intact, apparent shell collapse and degradation fragments (red circle) were also observed (Figure S36f, Supporting Information). In addition, a stronger *T*_1_‐weighted MRI signal was observed in the tumor‐bearing mouse treated with PMn_SA_GMSNs‐V injection than in control mice (Figure S36g, Supporting Information). As displayed in Figure S37, Supporting Information, the intensity of the MRI signal first increased and then decreased. At an injection time of 1 h, the intensity of MRI signal reached its maximum value. Notably, a much brighter tumor area (red circle) was observed after injection, indicating PMn_SA_GMSNs‐V accumulated in the tumor area and gradually released Mn^2+^ and Gd^3+^ via GSH, thereby providing an effective *T*_1_‐weighted MRI contrast for diagnosing tumors.

**Figure 5. a):**
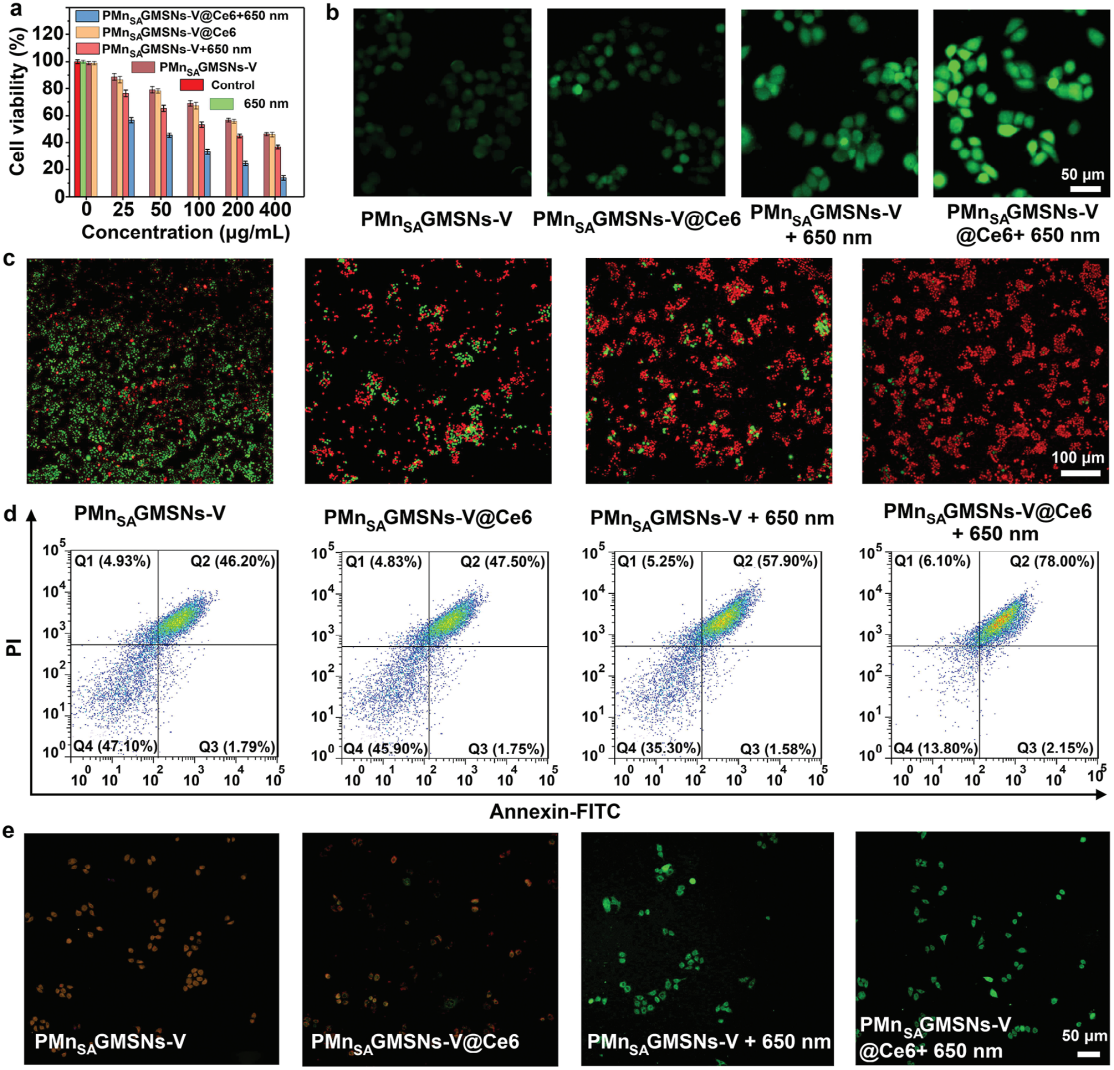
Viabilities of HeLa cells in the control group, treated with 650 nm, PMn_SA_GMSNs‐V, PMn_SA_GMSNs‐V plus 650 nm, PMn_SA_GMSNs‐V@Ce6 and PMn_SA_GMSNs‐V@Ce6 plus 650 nm (0.6 W cm^−2^, 10 min). b) Intracellular ROS detection using DCFH‐DA probe and c) CLSM images dyed with AM and PI of HeLa cells treated with PMn_SA_GMSNs‐V, PMn_SA_GMSNs‐V plus 650 nm, PMn_SA_GMSNs‐V@Ce6 and PMn_SA_GMSNs‐V@Ce6 plus 650 nm. Apoptosis of HeLa cells detected by flow‐cytometry in the groups of control, 650, PMn_SA_GMSNs‐V, PMn_SA_GMSNs‐V@Ce6, PMn_SA_GMSNs‐V plus 650, and d) PMn_SA_GMSNs‐V@Ce6 plus 650. CLSM images of HeLa cells stained by JC‐1 after incubation with PMn_SA_GMSNs‐V, PMn_SA_GMSNs‐V@Ce6, PMn_SA_GMSNs‐V plus 650 nm, and e) PMn_SA_GMSNs‐V@Ce6 plus 650 nm (error bars denote the standard deviation (*n* = 3, mean ± SD).

To improve the biocompatibility of Mn_SA_GMSNs‐V@Ce6, HS‐PEG was employed to synthesize PMn_SA_GMSNs‐V@Ce6 (Figure S38). The peaks located at 1072 and 2979 cm^−1^ were observed for PMn_SA_GMSNs‐V@Ce6 and were ascribed to the stretching vibration of C–H and –CH_2_–O–, indicating the successful modification of Mn_SA_GMSNs‐V@Ce6. The cellular phagocytosis behavior of PMn_SA_GMSNs‐V decorated by FITC was evaluated by the HeLa cells. As shown in Figure S39, Supporting Information, cell phagocytosis results for PMn_SA_GMSNs‐V at different times were collected by the confocal laser scanning microscopy (CLSM). In the overlay image, the nuclei and cytoplasm of HeLa cells were labeled with DAPI (blue fluorescent) and FITC (green fluorescent), respectively, indicating that more PMn_SA_GMSNs‐V were taken up by the HeLa cells over time. Subsequently, the in vitro cytotoxicity of PMn_SA_GMSNs‐V and PMn_SA_GMSNs‐V@Ce6 at the cellular level were studied by 4,5‐dimethylthiazol2‐yl‐2,5‐diphenyl tetrazolium bromide (MTT) method. The cell viabilities of L929 were calculated to be above 95% in each group when the concentration of PMn_SA_GMSNs‐V and PMn_SA_GMSNs‐V@Ce6 increased from 0 to 400 µg mL^−1^ (Figure S40, Supporting Information). These results revealed that PMn_SA_GMSNs‐V and PMn_SA_GMSNs‐V@Ce6 were non‐toxic to normal cells and had good biocompatibility. The cytotoxicity of PMn_SA_GMSNs‐V@Ce6 in the HeLa cells was investigated based on the synergy of CDT and PDT (**Figure** **5**a). The survival rate of HeLa cells in each group irradiated with 650 nm laser irradiation was as high as 96%, revealing that laser irradiation by 650 nm was non‐toxic to HeLa cells. Nevertheless, the viability of HeLa cells incubated with PMn_SA_GMSNs‐V decreased with increasing concentrations of PMn_SA_GMSNs‐V from 0 to 400 µg mL^−1^, suggesting that a large amount of ^•^OH was produced by high‐efficiency Mn single atomic active centers. Similarly, when Ce6 was integrated into PMn_SA_GMSNs‐V, the cells treated with PMn_SA_GMSNs‐V@Ce6 without laser irradiation exhibited a survival rate equivalent to that of PMn_SA_GMSNs‐V. When the concentration of PMn_SA_GMSNs‐V and PMn_SA_GMSNs‐V@Ce6 reached a maximum of 400 µg mL^−1^, the survival rates of HeLa cells were ≈46.1 and 45.9% in the existence of PMn_SA_GMSNs‐V and PMn_SA_GMSNs‐V@Ce6 alone, respectively. Then, when HeLa cells were exposed to 650 nm light illumination, it was obvious that the viabilities of cells incubated with PMn_SA_GMSNs‐V decreased to ≈36.8%, which was attributed to the increased ROS generation by inducing efficient separation of photogenerated electron‐holes. More importantly, the viability of HeLa cells decreased obviously to about 13.8% after treatment with PMn_SA_GMSNs‐V@Ce6 plus 650 nm light illumination (0.6 W cm^−2^) due to the additive effects of numerous ROS.

In general, cell apoptosis is often associated with the production of intracellular ROS.^[1c]^ To further investigate the reaction mechanism by which PMn_SA_GMSNs‐V@Ce6 kill cancer cells, an intracellular 2,7‐dichlorohydrofluorescein diacetate (DCFH‐DA) probe was used to study the production of ROS under different conditions based on the oxidized DCFH with green‐fluorescence signal.^[^ ^42^ ^]^ As shown in Figure S41, Supporting Information, no obvious green fluorescence signals were detected in the groups of control and 650 nm, suggesting that no ROS can be generated in the aforesaid treatment. Because of the active sites of MnSiO_3_ and atomic dispersed Mn in PMn_SA_GMSNs‐V, HeLa cells incubated with PMn_SA_GMSNs‐V exhibited green fluorescence (Figure 5b). For PMn_SA_GMSNs‐V@Ce6, HeLa cells incubated with PMn_SA_GMSNs‐V without 650 nm laser irradiation showed similar the fluorescence intensity similar to that of PMn_SA_GMSNs‐V. However, a stronger green fluorescence was observed in HeLa cells after treatment with PMn_SA_GMSNs‐V plus 650 nm, which was attributed to the multiple ROS generation originating from synergistic effects of the efficient separation of photogenerated electrons‐holes and atomic dispersed Mn sites. In addition, HeLa cells incubated with PMn_SA_GMSNs‐V@Ce6 with 650 nm laser irradiation presented strongest green fluorescence in all groups when Ce6 was introduced to PMn_SA_GMSNs‐V, following ESR results. Under the 650 nm irradiation, PMn_SA_GMSNs‐V acted as a photosensitizer, and Ce6 were excited simultaneously to generate ^1^O_2_. Meanwhile, PMn_SA_GMSNs‐V can not only trigger CAT effect to achieve the decomposition of H_2_O_2_ and efficiently convert H_2_O_2_ to O_2_ and H_2_O due to the introduction of Gd sites but also generate many electron‐hole pairs. On the one hand, the generation of O_2_ can further alleviate hypoxia in tumors. On the other hand, O_2_ will also serve as a substrate for PDT, thereby efficiently achieving ^1^O_2_ conversion. Then, photogenerated electrons in the CB of PMn_SA_GMSNs‐V can capture O_2_ to further produce ^•^O_2_^−^. While photogenerated holes in the VB of PMn_SA_GMSNs‐V cannot directly convert H_2_O into ^•^OH, but holes can combine with ^•^O_2_^−^ and convert it into ^1^O_2_ (11).^[2b]^ Benefiting from atomic dispersed Mn sites in PMn_SA_GMSNs‐V, PMn_SA_GMSNs‐V can generate ^•^OH and ^•^O_2_^−^ by POD‐like and OXD‐like activities, respectively.

(11)

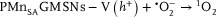




Subsequently, in response to TME (GSH, weakly acidic conditions), the PMn_SA_GMSNs‐V degraded and released Mn^2+^ to trigger a Fenton‐like chemical reaction to produce a large amount of ^•^OH. Because of the production of multiple ROS, the cancer cell‐killing efficiency of PMn_SA_GMSNs‐V@Ce6 based on the synergy of CDT and PDT was investigated using calcein‐acetoxymethyl ester (calcein‐AM) (green signals for representing living cells) and propidium iodide (PI) (red signals for representing dead cells), respectively.^[^ ^43^ ^]^ As shown in Figure S42, most HeLa cells presented green regions in the groups of control and 650 nm, indicating most of the HeLa cells maintained normal physiological activity. In contrast, the ratio of red regions was detected in PMn_SA_GMSNs‐V and PMn_SA_GMSNs‐V@Ce6 groups because of the Mn single atomic active centers and GSH depletion‐induced CDT reaction (Figure 5c). Upon 650 nm laser irradiation, the HeLa cells treated with PMn_SA_GMSNs‐V showed an increased ratio of red cells. For PMn_SA_GMSNs‐V@Ce6 plus 650 nm laser irradiation, the whole regions demonstrated the strongest red fluorescence compared to other groups, indicating PMn_SA_GMSNs‐V@Ce6 plus 650 nm possessed the highest killing ability for the HeLa cells owing to the synergistic results between PDT and CDT. Moreover, the effect of HeLa cells apoptosis was revealed by flow cytometry (Figure 5d and Figure S43). The corresponding proportions of tumor cells apoptosis for PMn_SA_GMSNs‐V, PMn_SA_GMSNs‐V@Ce6, PMn_SA_GMSNs‐V plus 650, and PMn_SA_GMSNs‐V@Ce6 plus 650 were evaluated to be 47.99, 49.25, 59.48 and 80.15%, respectively. Compared with other groups, the HeLa cells treated with PMn_SA_GMSNs‐V@Ce6 plus 650 nanozymes had severe mortality, consistent with MTT results.^[^ ^44^ ^]^

Many studies have shown that apoptosis is closely associated with mitochondrial destruction. Subsequently, the difference in the potential of mitochondrial membrane was detected by 1,1′,3,3′‐tetraethyl‐imidacarbocyanine (JC‐1) staining. Notably, JC‐1 mainly displayed red aggregation fluorescence in healthy mitochondria and green monomer fluorescence in unhealthy mitochondria.^[^ ^45^ ^]^ For control and 650 nm groups, the HeLa cells treated with by JC‐1 demonstrated the strongest red fluorescence in whole regions, suggesting no obvious changes in mitochondrial membrane potential (Figure S44, Supporting Information). However, for PMn_SA_GMSNs‐V and PMn_SA_GMSNs‐V@Ce6 without 650 nm laser irradiation, the strong fluorescence intensity ratio of green to red was observed in HeLa cells (Figure 5e). Importantly, the group treated with PMn_SA_GMSNs‐V@Ce6 plus 650 nm laser presented strong green monomer fluorescence, suggesting that the mitochondria of most HeLa cells were destroyed. Besides, the intratumoral GSH levels were further detected using glutathione detection reagent. As shown in Figure S45, Supporting Information, CLSM images display the strongest green fluorescence, indicating highest GSH levels in the groups of control and laser irradiation. Obviously, lower fluorescence signals were observed for PMn_SA_GMSNs‐V and PMn_SA_GMSNs‐V@Ce6 without laser irradiation. More importantly, CLSM images of PMn_SA_GMSNs‐V and PMn_SA_GMSNs‐V@Ce6 with 650 nm laser irradiation showed a sharp decrease in green fluorescence intensity, which was attributed to the GSH depletion. The HeLa cells treated with PMn_SA_GMSNs‐V@Ce6 and laser irradiation exhibited the lowest fluorescence intensity, indicating that the ROS consumed large amounts of GSH produced. In fact, CDT‐induced GSH depletion can prevent the loss of ROS, thereby achieving synergy between PDT and CDT.

**Figure 6. a):**
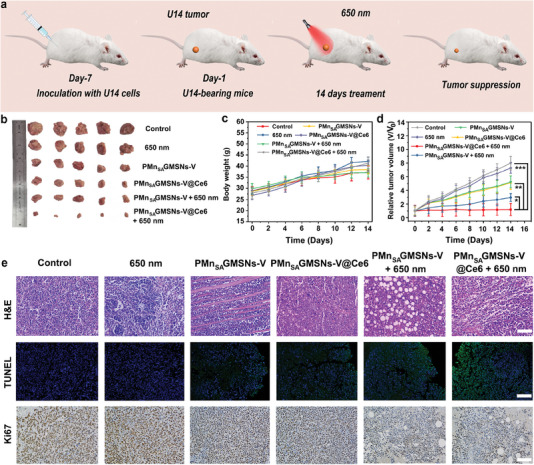
Schematic illustration of therapeutic outcome of PMn_SA_GMSNs‐V@Ce6 administrated by injection on U14‐tumor‐bearing mice. b) Photographs of dissected tumors from different treatments, c) body weight changes, and d) the relative tumor volume of U14 tumor‐bearing mice in 14 days. H&E, TUNEL, and Ki67 stained images of tumor tissues in the control group, treated with 650 nm, PMn_SA_GMSNs‐V, PMn_SA_GMSNs‐V plus 650 nm, PMn_SA_GMSNs‐V@Ce6 and PMn_SA_GMSNs‐V@Ce6 plus 650 nm treated groups after 14 days of treatment (e) (scale bar: 100 µm) (error bars denote the standard deviation (*n* = 5, mean ± SD, *p < 0.05, **p < 0.01, ***p < 0.001).

Benefiting from the satisfactory results on a cellular level, the in vivo antitumor experiments for PMn_SA_GMSNs‐V@Ce6 were systematically investigated in a U14 tumor‐bearing mouse model (**Figure** **6**a). First, based on the different treatment conditions, the tumor‐bearing animals were randomly divided into six groups including control, 650 nm laser irradiation only (650 nm), PMn_SA_GMSNs‐V, PMn_SA_GMSNs‐V@Ce6, PMn_SA_GMSNs‐V plus 650 nm and PMn_SA_GMSNs‐V@Ce6 plus 650 nm to evaluate the antitumor effects. In Figure 6b, photographs of tumors dissected from the different treatments are collected. Compared to control and 650 nm groups, PMn_SA_GMSNs‐V and PMn_SA_GMSNs‐V@Ce6 exhibited a low tumor suppression effect due to the atomically dispersed Mn only. However, when PMn_SA_GMSNs‐V@Ce6 was illuminated at 650 nm, PMn_SA_GMSNs‐V@Ce6 plus 650 nm demonstrated the highest effect of inhibiting tumor growth due to the synergistic effects of PDT by dual photosensitizers, CDT by GSH‐depleted and atomically dispersed Mn active center. Additionally, differences in the body weight and tumor volume were recorded to assess the therapeutic effect during 14 days of treatment (Figure 6c). In comparison with the groups of control and 650 nm, the weight of animals in the other groups was also normal and slightly increased over time, suggesting these treatments basically had no harmful effects on the health of mice. Subsequently, the relative tumor volumes of mouse was recorded for 14 days (Figure 6d). Compared with the control and 650 nm groups, limited suppression of tumor growth was observed in PMn_SA_GMSNs‐V and PMn_SA_GMSNs‐V@Ce6 groups. As expected, for the group of PMn_SA_GMSNs‐V@Ce6 plus 650 nm laser irradiation, much more obvious tumor inhibitory effects were observed, which was attributed to multiple ROS generation. Besides, the hematoxylin and eosin (H&E) stained images of main organs (Figure S46, Supporting Information), including heart, liver, spleen, lung, and kidney, showed no obvious damage or inflammation, indicating the excellent histocompatibility and biosafety of PMn_SA_GMSNs‐V@Ce6. Subsequently, the H&E stained images of representative tumor issues from different groups were also examined to verify the destruction of tumor cells. As shown in Figure 6e, PMn_SA_GMSNs‐V@Ce6 plus 650 nm group exhibited a remarkable decrease in the proportion of blue‐stained nuclei, verifing that PMn_SA_GMSNs‐V@Ce6 plus 650 nm treatment displayed the maximum destruction level to tumor cells. Besides, Ki67 staining and terminal deoxynucleotidyl transferase‐mediated deoxyuridinetriphosphate nick end labeling (TUNEL) were used to evaluate the tumor apoptosis and proliferation. Compared with other groups, TUNEL and Ki67 stained tumor tissue slices showed that many of tumor cells were necrotic and the proliferation of tumor cells was reduced in the PMn_SA_GMSNs‐V@Ce6 plus 650 nm group. To evaluate the biosafety of PMn_SA_GMSNs‐V@Ce6, the corresponding the Mn and Si contents from tumor tissues and the main organs at various time point after injection were detected by biodistribution in vivo (Figure S47 and Figure S48, Supporting Information). Owing to the capture of blood circulation and reticuloendothelial system, the injected PMn_SA_GMSNs‐V@Ce6 nanozymes were mainly located in the liver and tumor, suggesting that nanozymes possessed efficient excretion capacity and that the toxicity of PMn_SA_GMSNs‐V@Ce6 to mice during treatment was negligible. These results indicated PMn_SA_GMSNs‐V@Ce6 could act as an efficient antitumor agent with high biosafety because of the multiple ROS generation in acidic tumor microenvironment.

## 3 Conclusion

In summary, a mass‐produced SAzymes synthetic strategy based on OVs‐immobilization by a simple hydrothermal process was developed to prepare TME‐activable PMn_SA_GMSNs‐V@Ce6 with atomically dispersed Mn/Gd dual sites for synergistic CDT/PDT. OVs induced by co‐doping of Gd and Mn ions into SiO_2_ can capture unbound metal ions to form atomically dispersed Mn and Gd, as verified by the XANES and HAADF‐STEM. The local electrons of atom‐catalyzed Mn active sites were modulated by the introduction of Gd sites, thereby achieving effective CAT‐like‐, OXD‐like‐ and POD‐like‐ enzyme activities. Systemic DFT calculation results revealed that the O–O bond of endogenous H_2_O_2_ could be elongated and weakened due to the presence of OVs, thereby promoting the decomposition of endogenous H_2_O_2_. Importantly, PMn_SA_GMSNs‐V@Ce6 can serve as dual photosensitizers to generate ^1^O_2_ upon 650 nm light excitation. The PMn_SA_GMSNs‐V‐triggered CAT effect is beneficial for O_2_ generation and favorable of PDT. In addition, laser‐irradiated PMn_SA_GMSNs‐V can generate several electron‐hole pairs to facilitate the generation of ^•^O_2_^−^. Significantly, intratumoral GSH triggered the biodegradation of PMn_SA_GMSNs‐V to liberate Fenton‐like Mn^2+^ that reacted with H_2_O_2_ to produce ^•^OH for self‐enhanced CDT. The in vivo experiments results revealed that PMn_SA_GMSNs‐V@Ce6 had the highest anticancer efficacy due to effective GSH depletion and multiple ROS. Interestingly, tumor GSH‐induced release of Mn^2+^ and Gd^3+^ exhibited ideal *T*_1_ relaxivity for tumor‐enhanced MRI. Our work provides a novel strategy to encapsulate atomically dispersed Mn/Gd dual sites by OVs‐anchored in a biodegradable nano‐diagnostic platform with dual photosensitizers to achieve synergistic effects of GSH‐depletion enhanced CDT and hypoxia‐improved PDT.

## Conflict of Interest

The authors declare no conflict of interest.

## Supporting information

Supporting Information

Supporting Information

## Data Availability

The data that support the findings of this study are available on request from the corresponding author. The data are not publicly available due to privacy or ethical restrictions.
