# Supplementary material for: Embedding Atomically Dispersed Manganese/Gadolinium Dual Sites in Oxygen Vacancy‐Enriched Biodegradable Bimetallic Silicate Nanoplatform for Potentiating Catalytic Therapy
Source: Adv Sci (Weinh). 2023 Nov 30;11(4):2307424. doi: 10.1002/advs.202307424 (PMC10962490; doi:10.1002/advs.202307424)
Supplement: Supplementary file 1 — Supporting Information [file ADVS-11-2307424-s002.pdf]

ADVANCED SCIENCE
Open Access

Supporting Information

for *Adv. Sci.*, DOI 10.1002/advs.202307424

Embedding Atomically Dispersed Manganese/Gadolinium Dual Sites in Oxygen Vacancy-Enriched Biodegradable Bimetallic Silicate Nanoplatform for Potentiating Catalytic Therapy

Jin Ye, Kefen Zhang, Xing Yang, Mengting Liu, Yujie Cui, Yunlong Li, Chunsheng Li, Shuang Liu, Yong Lu, Zhiyong Zhang, Na Niu, Ligang Chen, Yujie Fu* and Jiating Xu**

Supporting Information

Embedding Atomically Dispersed Manganese/Gadolinium Dual Sites in Oxygen Vacancy-Enriched Biodegradable Bimetallic Silicate Nanoplatform for Potentiating Catalytic Therapy

Jin Ye, Kefen Zhang, Xing Yang, Mengting Liu, Yujie Cui, Yunlong Li, Chunsheng Li, Shuang Liu, Yong Lu, Zhiyong Zhang, Na Niu, Ligang Chen, Yujie Fu,* and Jiating Xu**

Experimental Section

Chemicals and Reagents. Hexadecyltrimethylammonium p-toluenesulfonate (CTA·Tos) and triethanolamine (TEOA) were obtained from the Tianjin Fengchuan Chemical Reagent Co., Ltd. 1-butyl-3-methylimidazolium trifluoromethanesulfonate ([BMIM] OTF) and tetraethoxysilicon (TEOS) were purchased from Sigma-Aldrich. Manganese (II) chloride tetrahydrate ($MnCl_2·4H_2O$) and ethanol (C_2H_5OH) were got from Tianjin Institute of Chemical Agents. $GdCl_3$ was gained from Sinopharm Chemical Reagent Co., Ltd. Ammonium chloride (NH_4Cl) and ammonium hydroxide ($NH_3·H_2O$) were derived from Shanghai Macklin Biochemical Co., Ltd. And 3-(4, 5-dimethyl-2-thiazolyl)-2,5-diphenyl-2-H-tetrazolium bromide (MTT), dimethyl sulfoxide (DMSO) and 4′, 6-diamidino-2-phenylindole (DAPI) were acquired from Sigma-Aldrich. Photosensitizer chlorin e6 (Ce6) was obtained from Shanghai Macklin Biochemical Co., Ltd. Thiol-polyethylene glycol (HS-PEG), 1,3-Diphenylisobenzofuran (DPBF) and Dulbecco's modified Eagle's medium (DMEM) were received from Aladdin Reagents Company Co., Ltd. 2′,7′,2-dichlorodihydrofluorescein diacetate (DCFH-DA) was order from Sigma-Aldrich. 5,5-dithiobis (2-nitrobenzoic acid) (DTNB) and methylene blue (MB) were acquired from Sigma-Aldrich. Calcein-acetoxymethyl ester (calcein-AM) and propidium iodide (PI) were acquired from Beijing Solarbio Science & Technology Co., Ltd. 1,1′,3,3′-tetraethyl-imidacarbocyanine (JC-1), thiol-polyethylene glycol (HS-PEG) and H_2O_2 (30%) were purchased from Macklin Biochemical Technology Co., Ltd. Distilled water was used in the experiment. All of the chemical reagents were used without further purification.

Characterization

The high-angle annular dark-field scanning transmission electron microscope (HAADF-STEM, Thermo Scientific, Themis Z) was employed to observe the existence of single atom. Mn K-edge and Gd L_3-edge EXAFS and XANES spectra were performed with Si (111) crystal monochromators at the BL11B beamlines at the Shanghai Synchrotron Radiation

Facility (SSRF) (Shanghai, China). X-ray powder diffractometer (Shimadzu XRD-7000) was employed to investigate the crystallographic properties of the catalyst. The morphology of the sample was analyzed by scanning electron microscope (SEM, JSM-7500F) and transmission electron microscope (JEM-2100) (Japan electronics). Fourier-transform infrared spectra (FT-IR) were performed on NicoletiS10. Energy disperse X-ray (EDX) was performed on a field emission scanning electron microscope (JSM-7500F) to analyze elemental features of the samples. The ultraviolet-visible-infra-red (UV-vis-NIR) spectrometry was measured on UH5700 spectrophotometer. The electron spin resonance (ESR) spectra were examined on a Bruker ELEXSYS-II E500 CW-EPR. The cell fluorescence imaging experiments were carried out using confocal laser scanning fluorescence microscope (CLSM). Photoluminescence (PL) spectroscopy was recorded via an Agilent Cary Eclipse (F-7000) at room temperature. Time-resolved photoluminescence (TRPL) spectra for samples were conducted from 350 to 600 nm with a photoluminescence spectrometer (QM8000) at an excitation 325 nm. All electrochemical and photoelectrochemical characterizations were performed on an electrochemical workstation (CHI660E) using a three-electrode system (pH 7.0) with Pt as the counter electrode, Ag/AgCl as the reference electrode, and ITO as the working electrode under visible light. In Mott-Schottky plots, the test frequency is 1000 Hz and the potential range is -1 to 1 V. The chemical composition and element valence were analyzed using X-ray photoelectron spectroscopy (XPS, Kratos Axis Ultra) with a monochromated Al Kα X-ray source (hν=1486.6 eV). N_2 adsorption-desorption isotherm measurements were investigated to study the Brunauer-Emmett-Teller (BET) specific surface areas of the samples.

Computational Methods

We have employed the Vienna Ab Initio Package (VASP)[1] to perform all the density functional theory (DFT) calculations within the generalized gradient approximation (GGA) using the PBE formulation.[2] We have chosen the projected augmented wave (PAW) potentials [3] to describe the ionic cores and take valence electrons into account using a plane

wave basis set with a kinetic energy cutoff of 450 eV. Partial occupancies of the Kohn−Sham orbitals were allowed using the Gaussian smearing method and a width of 0.05 eV. The electronic energy was considered self-consistent when the energy change was smaller than 10^{-4} eV. A geometry optimization was considered convergent when the force change was smaller than 0.05 eV $Å^{-1}$. Grimme's DFT-D_3 methodology[4] was used to describe the dispersion interactions. The Brillouin zone integral uses the surfaces structures of 2×2×1 monkhorst pack K-point sampling. Finally, the adsorption energies (E_{ads}) were calculated as:

$$E_{ads}= E_{ad/sub} -E_{ad} -E_{sub} \quad (1)$$

where $E_{ad/sub}$, E_{ad}, and E_{sub} are the total energies of the optimized adsorbate/substrate system, the adsorbate in the gas phase, and the clean substrate, respectively. The free energy (ΔG) for elemental reaction steps were calculated as:

$$\Delta G = \Delta E + \Delta E_{ZPE}- T\Delta S \quad (2)$$

where ΔE is the difference between the total energy, ΔE_{ZPE} and ΔS are the differences in the zero-point energy and the change of entropy, T is the temperature (T =300 K in this work), respectively. The U correction had been adopted in our systems, and fixed the section atoms.

Synthesis of Mesoporous Silica Nanoparticles (MSNs)

MSNs with monodisperse were successfully prepared according to previous method. In a word, 0.96 g of CTA·Tos, 0.105 g of TEOA, and 10 mg of [BMIM] OTF were first dissolved in 50 mL of water, and stirred at 80 °C for 1 h to obtain a mixable solution. Then, 7.8 mL of TEOS was quickly added into the above mixture. Subsequently, the mixed solution was maintained at 80 °C for another 2 h. After the reaction was cooled to room temperature, the resultant white sample was separated by centrifugation and washed with H_2O and methanol several times to remove the residual ions. In order to remove CTA·Tos template, as-prepared sample was added into ethanol. After the suspension was sonicated and stirred for 2 h, then MSNs was collected by washing with ethanol and drying in a vacuum oven at 70 °C for 12 h.

Preparation of Mn_{SA}GMSNs-V

Atomically dispersed Mn/Gd dual sites embedded in the oxygen vacancy-enriched mesoporous gadolinium and manganese co-doped silicate nanospheres (Mn_{SA}GMSNs-V) were successfully synthesized by one-step hydrothermal method using MSNs as a template. In a typical method, 50 mg MSNs and $MnCl_2·4H_2O$ (0.08 g, 0.4 mmol) were added in 20 mL water to form uniform solution A. Furthermore, NH_4Cl (0.54 g, 10 mmol), $GdCl_3$ (0.105 g, 0.4 mmol) and 1 mL of $NH_3·H_2O$ (28%) were dissolved in 20 mL of water to obtain uniform solution B. Then, solutions A and B were quickly mixed and stirred for 30 min. Then, above mixture solution was sealed in a 100 mL Teflon-lined autoclave and maintained at 180 °C for 24 h. After the autoclave was cooled to room temperature, the resultant product was separated *via* centrifugation and washed with ethanol several times to remove residual ions. The final products were placed in an oven and dried overnight at 50 °C. Besides, $MnCl_2$ and $GdCl_3$ were doped into SiO_2 under the same conditions and the corresponding products were labeled as $MnSiO_3$ and Gd/SiO_2, respectively.

Ce6 Loading

Atomically dispersed Mn/Gd dual sites embedded in the oxygen vacancy-enriched mesoporous gadolinium and manganese co-doped silicate nanospheres (Mn_{SA}GMSNs-V@Ce6) were successfully synthesized by simple method. Firstly, 20 mg Ce6 was dissolved in 25 mL DMSO containing 1-Ethyl-3-(3-dime thylaminopropyl) carbodiimide (EDC) and N-hydroxysulfosuccinimide sodium salt (sulfo-NHS) and stirred for 2 h to obtain activated carboxyl group (solution A). Then, 100 mg Mn_{SA}GMSNs-V and 300 μL APTES were dissolved and stirred in 25 mL DMSO for 2h (solution B). Then, solutions A and B were quickly mixed and stirred for 12 h. Finally, the resultant product was separated *via* centrifugation and washed with ethanol several times to remove residual ions.

Modification with PEG

Coupling Chlorin e6 (Ce6) to synthesize PEGylated atomically dispersed Mn/Gd sites embedded in the mesoporous gadolinium and manganese silicate nanospheres with oxygen

vacancies ($PMn_{SA}GMSNs$-V@Ce6) was prepared by one-step synthesis. Firstly, 100 mg $Mn_{SA}GMSNs$-V@Ce6 nanospheres were dissolved in 50 mL H_2O, and then 100 mg HS-PEG was added into above solution under stirring. Secondly, the mixed solution was stirred overnight at room temperature. Finally, the product was further collected by centrifugation, washed with ethanol three times, and dried at 60 °C overnight.

Peroxidase-Like Activity of $PMn_{SA}GMSNs$-V

The peroxidase (POD)-like activities of $PMn_{SA}GMSNs$-V (200 μg mL^{-1}) were performed using MB (400 μM) as substrates in the presence of H_2O_2 (400 mM) and PBS solution (pH 6.5). The absorbance of the MB was recorded after a certain reaction time using a UV-vis-NIR spectrophotometer at 664 nm.

Catalase-Like Activity of $PMn_{SA}GMSNs$-V

The catalase (CAT)-like activity of $Mn_{SA}GMSNs$-V was evaluated at 25 °C by measuring the O_2 production. Specifically, 3 mL H_2O_2 was mixed with 1 mL H_2O, followed by the addition of 4 mg $Mn_{SA}GMSNs$-V (1.0 mg mL^{-1}). As a control, the other group only added H_2O_2 and SiO_2. Then we recorded the experimental phenomenon of the by taking videos.

MB Degradation by ROS

In order to verify the generation of ROS ($\bullet O_2^-$ and •OH), methylene blue (MB) indicator was employed to record the absorbance of MB. $Mn_{SA}GMSNs$-V (200 μL, 3 mg mL^{-1}) and NaCl solution (200 μL, 50 mM) were added into PBS (pH 6.5, 800 μL) containing a series of different concentrations of H_2O_2 (0, 0.5, 1, 5, 10 mM). And then, 100 μL of MB (100 μg mL^{-1}) was added into the mixture solution and incubated at 37 °C for 0.5 h. Additionally, 1,4-Benzoquinone (BQ) was used to act as a free radical scavenger for $\bullet O_2^-$, respectively. Finally, the supernatant was washed by centrifugation and tested the absorbance of MB at 664 nm.

Detection of ROS by ESR

For ESR detection, TEMPO and DMPO were employed as the $^1O_2$, •OH and $\bullet O_2^-$ trapping agent, respectively. In a word, $Mn_{SA}GMSNs$-V (1 mg) and $Mn_{SA}GMSNs$-V@Ce6 (1 mg)

with or without H_2O_2 (1.0 mL) and GSH (10 mM) were added into the weak acidic buffer (pH 6.5) and 100 μM DMPO, respectively. Then, above the mixture solutions were transferred to a quartz tube for ESR assay after mixing by sonication for 1 min.

In Vitro Cellular Uptakes

A confocal laser scanning microscope (CLSM) was employed to investigate cell phagocytosis behavior. The FITC-labeled PMn_{SA}GMSNs-V@Ce6 was prepared using the following method. Ethanol solutions (20 mL) containing FITC (2 mg mL^{-1}) and APTES (100 μL mL^{-1}) were stirred vigorously together with 10 mg of Mn_{SA}GMSNs-V for 24 h to synthesize FITC-modified Mn_{SA}GMSNs-V@Ce6. Then, HeLa cells were seeded in a 6-well culture plate, followed by culturing the cells overnight and thus obtaining a monolayer. After that, the FITC-labeled PMn_{SA}GMSNs-V@Ce6 was added to the culture plates and further incubated the cells for 1 h, followed by washing with PBS for three times. Next, the HeLa cells were further stained with DAPI for 10 min. Subsequently, the cells were fixed with 1 mL glutaraldehyde at a concentration of 2.5% for 10 min, and then washed several times with PBS. Finally, the fluorescence images were recorded by CLSM.

In Vitro Biocompatibility

In order to access the in vitro biocompatibility of the as-prepared sample, a typical MTT method was carried out. After seeding L929 cells in a 96-well plate, cultured the cells in a humid incubator (37 °C, 5% CO_2) for 24 h to obtain monolayer L929 cells. PMn_{SA}GMSNs-V@Ce6 and PMn_{SA}GMSNs-V were diluted by culture medium to solutions with concentrations of 0, 25, 50, 100, 200, and 400 μg mL^{-1}, then the samples were put into each well and further incubated for another 3 h to ensure cell uptake. Subsequently, the c·ulture was removed, then after MTT solution (20 μL, 5 mg mL^{-1}) was added, incubate the cells for further 4 h. Afterward, DMSO (150 μL) was added into the culture wells, and the absorbance

at 490 nm was recorded after 10 min. The proportion of living cells between the experimental group and the control group is calculated to express the viabilities.

In Vitro Cytotoxicity

The cytotoxicity of the control, 650 nm, PMn_{SA}GMSNs-V, PMn_{SA}GMSNs-V plus 650 nm, PMn_{SA}GMSNs-V@650 nm and PMn_{SA}GMSNs-V@Ce6 plus 650 nm laser against HeLa cells were measured. HeLa cells were seeded into 96-well plates in 5% CO_2 at 37 °C for 24 h to obtain adherent cells. A sample solution of volume 100 μL and of different concentrations of (0, 25, 50, 100, 200 and 400 μg mL^{-1} in DMEM) was added to each well, and then, the cells were further incubated for 3 h to ensure cell uptake. And the power density of the 650 nm laser is 0.6 W cm^{-2}. Then, 20 μL of MTT solution (5 mg mL^{-1}) was added into each well, and the cells were incubated for another 4 h. The absorbance at 490 nm was obtained to calculate the cell survival rate using the plate reader.

Detection of Intracellular ROS Production

DCFH-DA was employed to detect the intracellular ROS generation ability of the PMn_{SA}GMSNs-V@Ce6. First of all, HeLa cells were seeded onto 6-well plates and incubated for 24 h. Then, these HeLa cells can be divided into four groups including control, 650 nm, PMn_{SA}GMSNs-V (1 mg mL^{-1}), PMn_{SA}GMSNs-V plus 650 nm (1 mg mL^{-1}), PMn_{SA}GMSNs-V@Ce6 (1 mg mL^{-1}) and PMn_{SA}GMSNs-V@Ce6 plus 650 nm (1 mg mL^{-1}). After incubation for 4 h, DCFH-DA (1 mL) was added into above 4 groups, respectively. Then, the four groups of HeLa cells have be incubated in the dark for another 10 min and washed three times with PBS.

Flow Cytometric Analysis of Apoptosis

Apoptosis was investigated using FITC/PI double staining. The HeLa cells were seeded in 6-well plates and incubated for 24 h. After the indicated treatments, cells were trypsinized, washed, and re-suspended in 195 μL of binding solution, followed by the incubation with the

5 μL Annexin V-FITC and 10 μL PI in dark for 15 min. Then, the HeLa cells were analyzed immediately using a flow cytometer (CytoFLEX S).

Bio-TEM

The HeLa cells were incubated with a DMEM medium containing PMn_{SA}GMSNs-V@Ce6 (0.5 mg mL^{-1}), 5% CO_2 at 37 °C for 24 h. After that, cells were washed with PBS for 3 times and further fixed with 2.5% glutaraldehyde in 0.03 M potassium phosphate buffer for more than 24 h. Finally, the images were obtained on JEOL2100 electron microscope.

Intracellular GSH Measurement

The HeLa cells were seeded in 6-well plates and incubated for 24 h. Then, the HeLa cells were treated with PBS, 650 nm, PMn_{SA}GMSNs-V (1 mg mL^{-1}), PMn_{SA}GMSNs-V (1 mg mL^{-1}) plus 650 nm, PMn_{SA}GMSNs-V@Ce6 (1 mg mL^{-1}), PMn_{SA}GMSNs-V@Ce6 (1 mg mL^{-1}) plus 650 nm for 12 h, respectively. After that, the contents of intracellular GSH were determined by GSH assay kit according to the manufacturer's protocol.

In Vivo Antitumor Evaluation

Female Kunming mice (18~22 g) were purchased from Second Affiliated Hospital of Harbin Medical University (Harbin, China) and all animal experiments were approved by the Ethics Committee of the Second Affiliated Hospital of Harbin Medical University. Animal experimental procedures were executed in accordance with the Guidelines for Care and Use of Laboratory Animals of the Drug Safety Evaluation Center of Harbin Medical University (No. SYDW 2019-82). Typically, each female mouse was inoculated with a tumor at the site of its right forelimb. When the tumor size grew to 120 mm^3, the mice were randomly divided into 6 groups (n = 5 per group) and treated with PBS (10 mM, 200 μL) injection (control), 650 nm (0.6 W cm^{-2}, 10 min) irradiation, PMn_{SA}GMSNs-V (2 mg mL^{-1}, 200 μL) injection, PMn_{SA}GMSNs-V (2 mg mL^{-1}, 200 μL) plus 650 nm (0.6 W cm^{-2}, 10 min) irradiation, PMn_{SA}GMSNs-V@Ce6 (2 mg mL^{-1}, 200 μL) and PMn_{SA}GMSNs-V@Ce6 (2 mg mL^{-1}, 200 μL) plus 650 nm (0.6 W cm^{-2}, 10 min) irradiation, respectively. The body weight was

recorded every 2 days after the first treatment. After 14 days, the cancer tissues and main organs were collected from the sacrificed mice for histological analysis.

Histological Examination

The tissues of liver, lung, kidney, heart, and spleen of control, 650 nm, PMn_{SA}GMSNs-V, PMn_{SA}GMSNs-V plus 650 nm, PMn_{SA}GMSNs-V@Ce6 and PMn_{SA}GMSNs-V@Ce6 plus 650 nm treated mice were excised and sliced into less than 1 cm × 1 cm, dehydrated successively with buffered formalin, ethanol of different concentrations, and xylene in turn after 14 days of treatment. Finally, all dehydrated tissues were embedded in liquid paraffin, sections were stained with hematoxylin and eosin (H&E), and observed using an optical microscope. In addition, the tumor tissues of control, 650 nm, PMn_{SA}GMSNs-V, PMn_{SA}GMSNs-V plus 650 nm, PMn_{SA}GMSNs-V@Ce6 and PMn_{SA}GMSNs-V@Ce6 plus 650 nm were also observed.

Statistical Analysis

The experimental data were expressed quantitatively as mean ± standard error of the mean using unpaired Student's t-test for comparison (n=5 each group, mean ± SD). Each experiment included at least three replicates. A statistical significance of $p < 0.05$ was selected, with (*) for $p < 0.05$, (**) for $p < 0.01$, and (***) for $p < 0.001$, respectively. The Origin 2017 software was used for graph plotting.

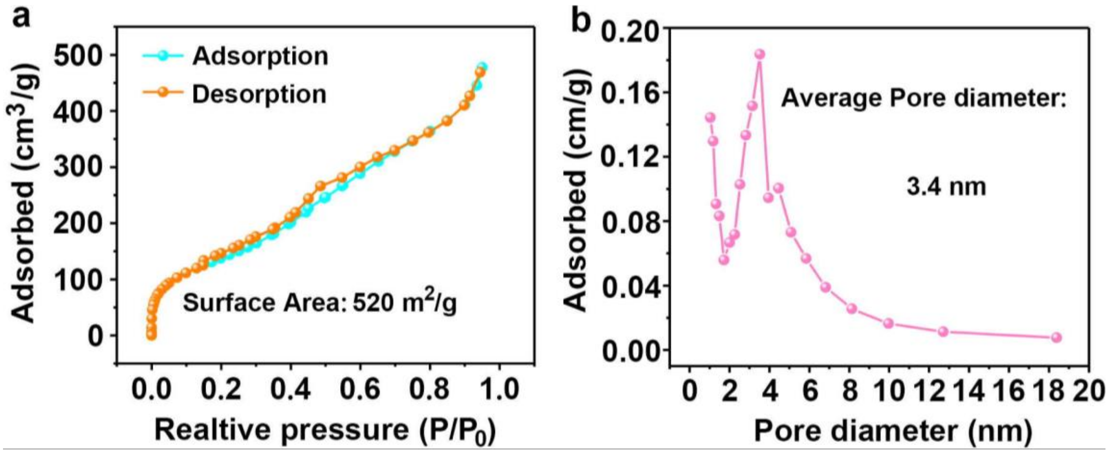

Figure S1. N_2 adsorption-desorption isotherms (a) and the corresponding pore-size distribution (b) of pure SiO_2.

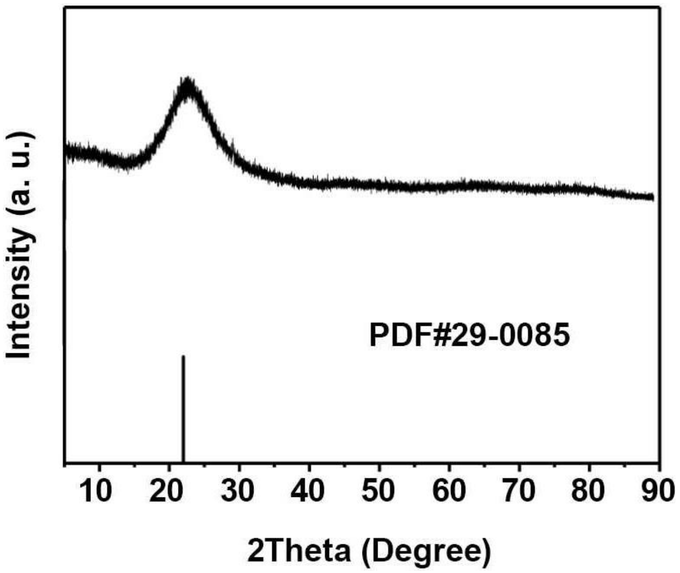

Figure S2. XRD patterns of pure SiO_2.

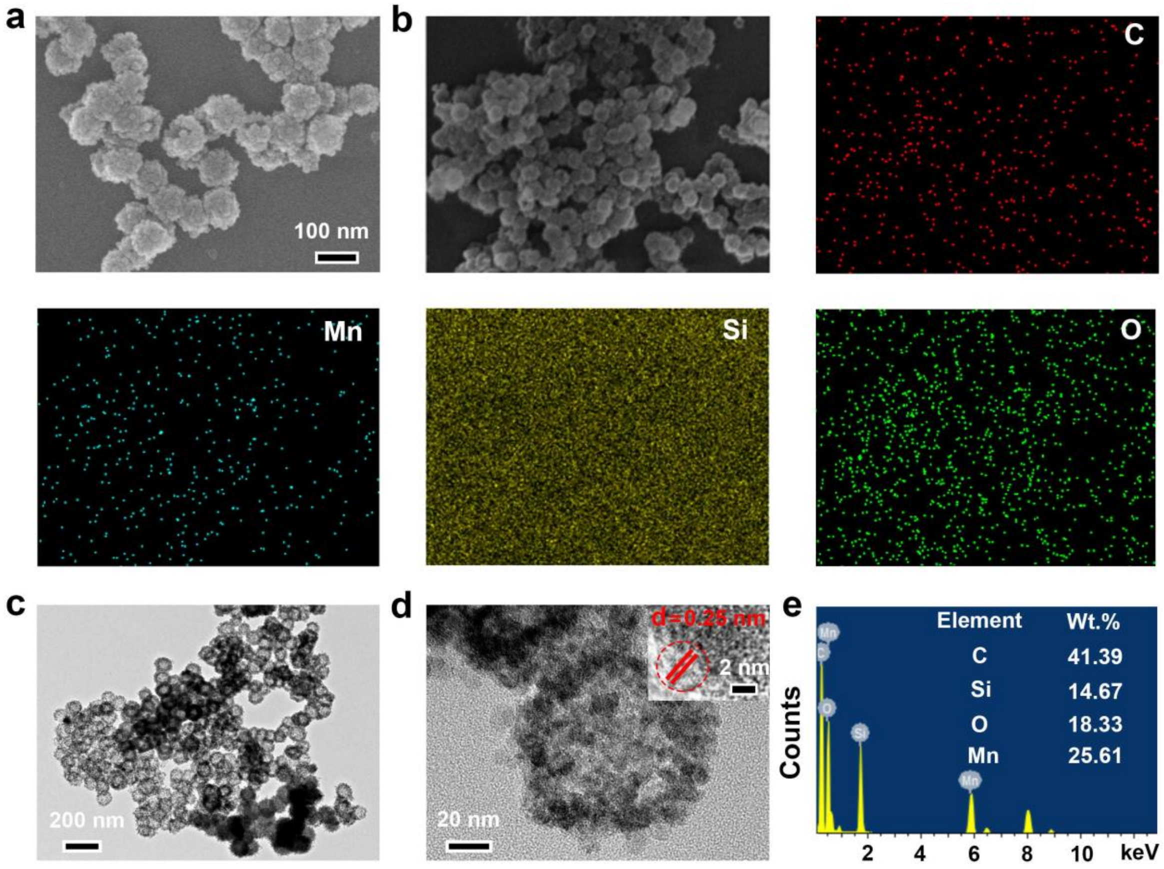

Figure S3. SEM image (a) and the corresponding SEM-EDS mapping (b), TEM images (inset: the HRTEM image of pure $MnSiO_3$) (c, d) and the corresponding EDS of $MnSiO_3$ (e).

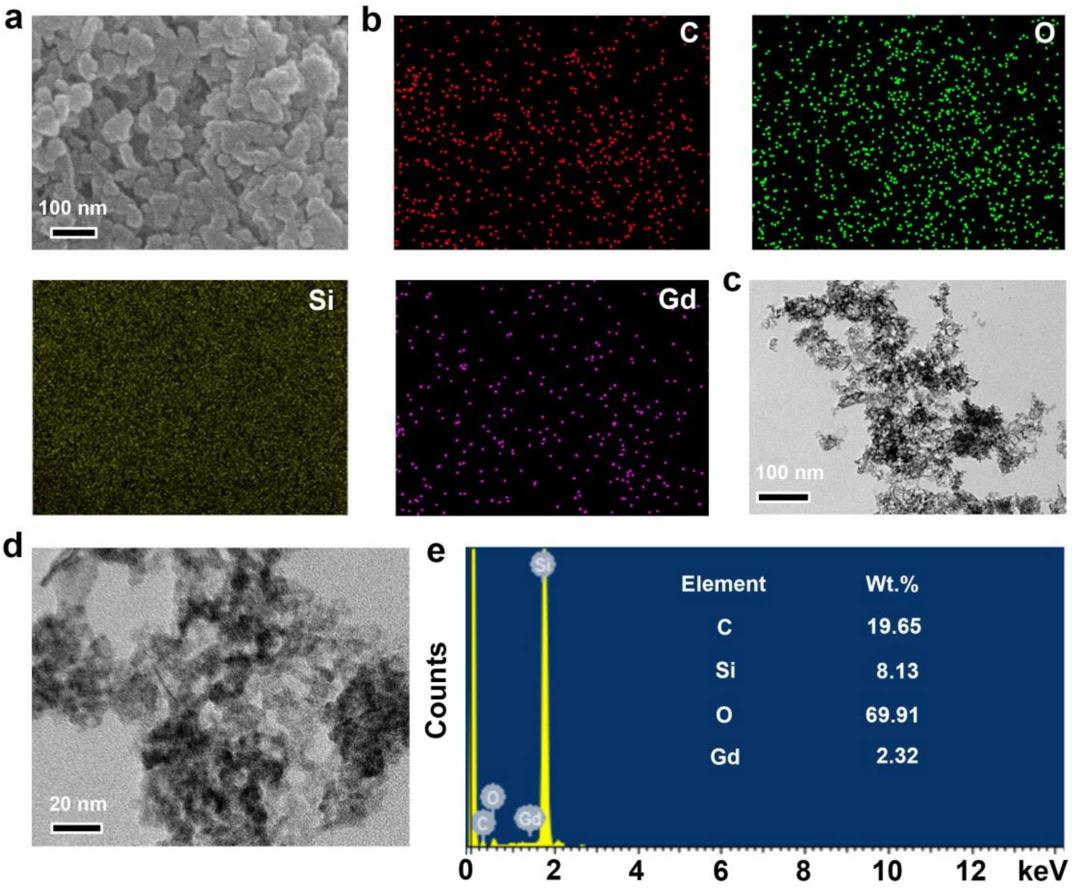

Figure S4. SEM image (a) and the corresponding SEM-EDS mapping (b), TEM images (c, d) and the corresponding EDS of Gd/SiO_2 (e).

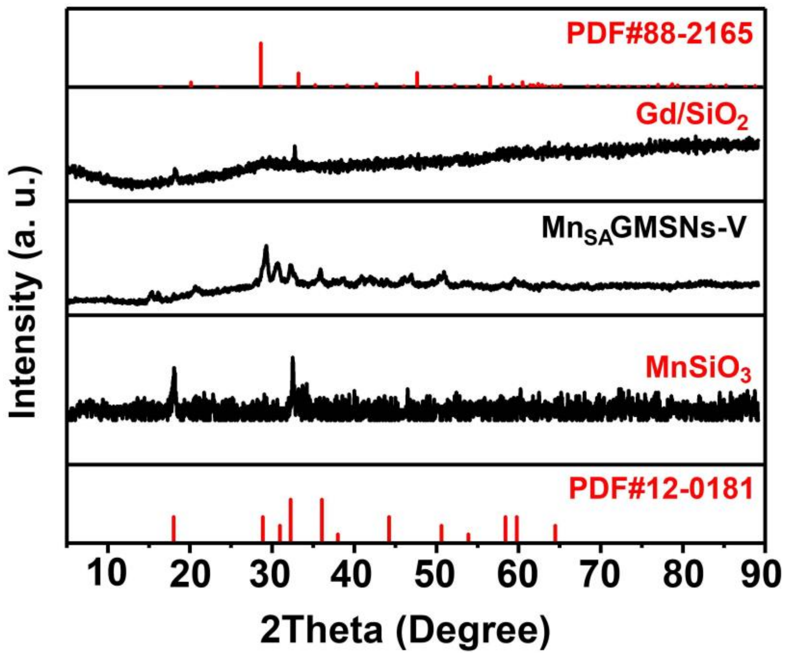

Figure S5. XRD patterns of pure $MnSiO_3$, Gd/SiO_2 and Mn_{SA}GMSNs-V.

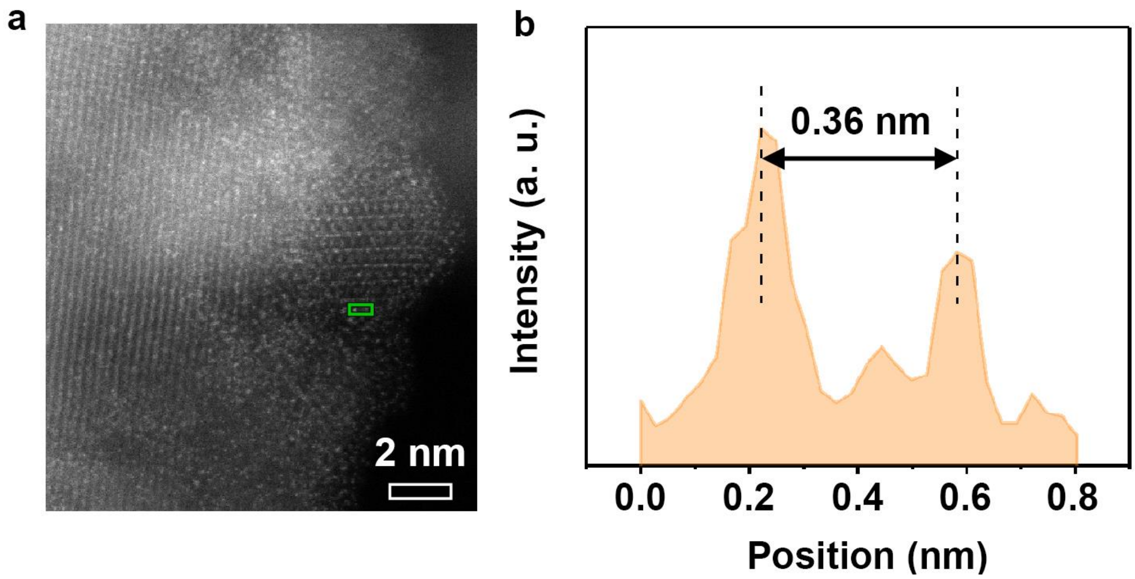

Figure S6. HAADF-STEM image of Mn_{SA}GMSNs-V (a) and the corresponding line-scanning intensity profiles (b).

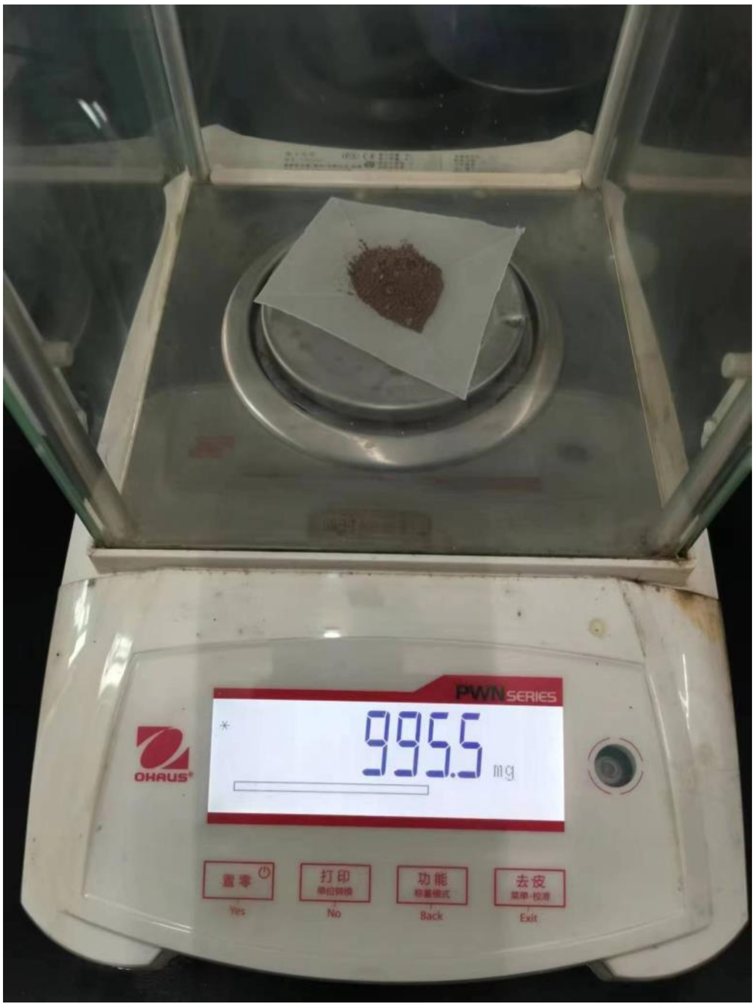

Figure S7. The digtal photographs of the synthesized Mn_{SA}GMSNs-V using 300 mg SiO_2 from a 300 mL reactor.

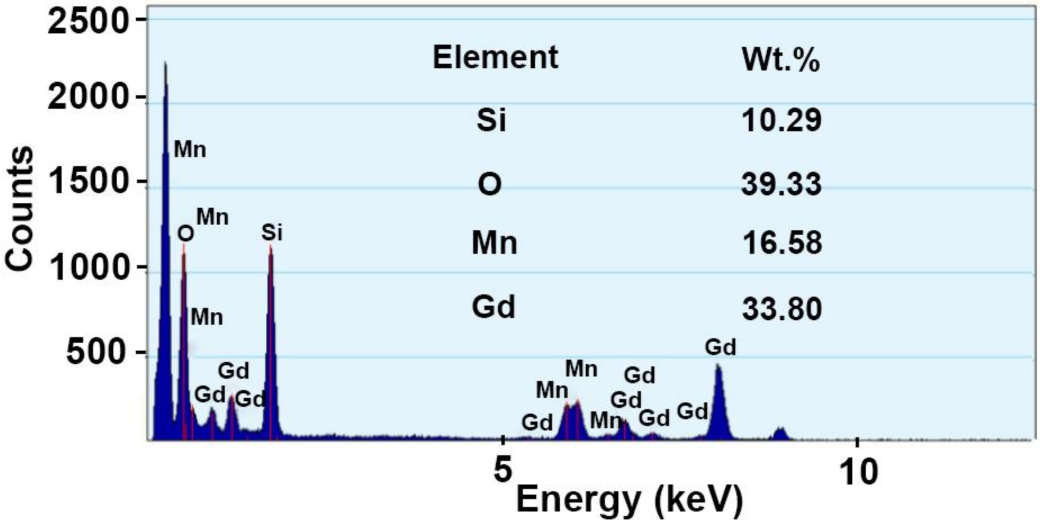

Figure S8. EDS of Mn_{SA}GMSNs-V.

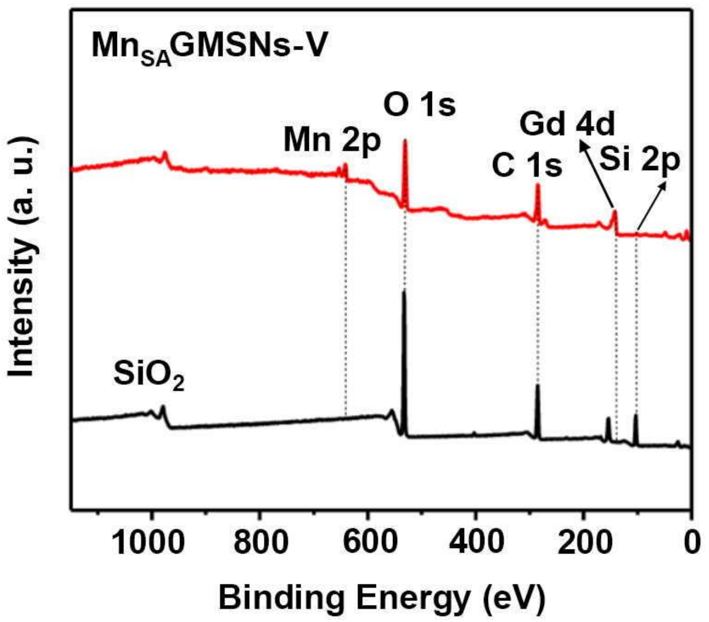

Figure S9. XPS survey spectra of SiO_2 and Mn_{SA}GMSNs-V.

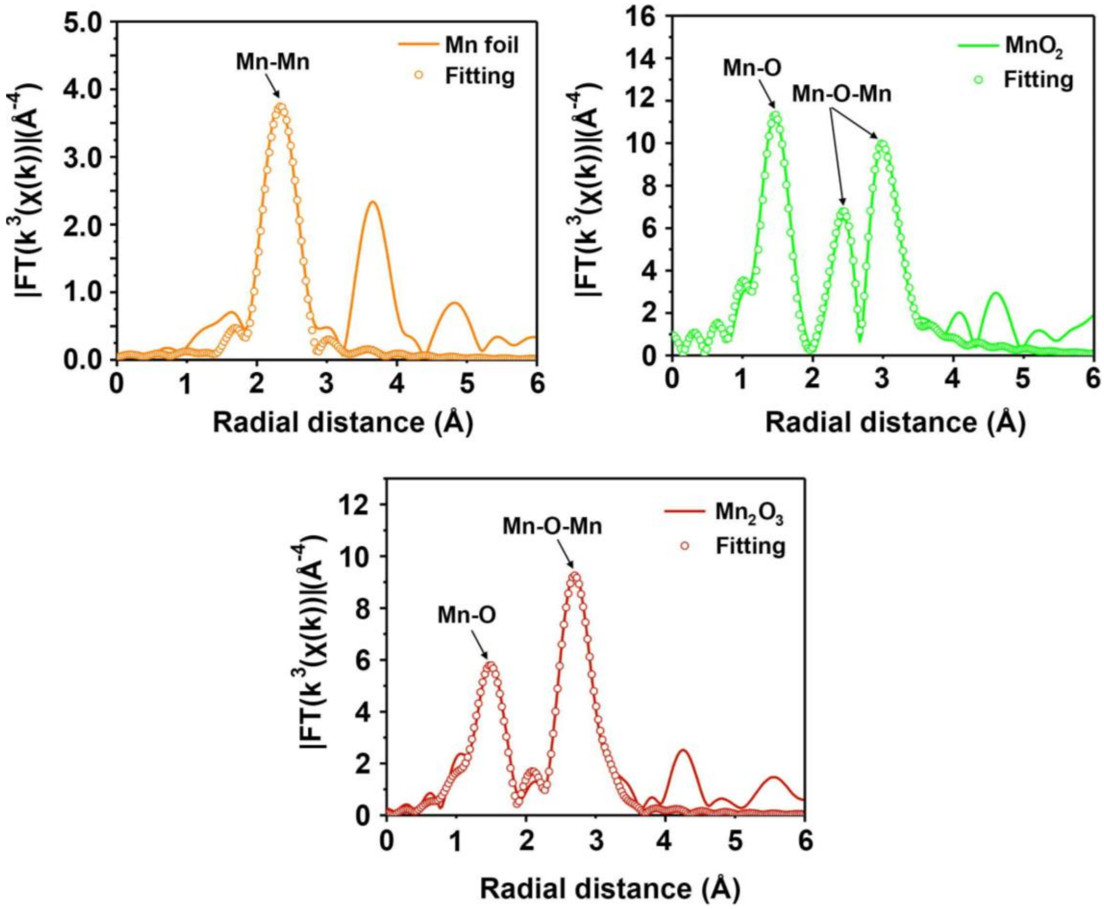

Figure S10. FT k^3-weighted EXAFS fitting curves at R space of Mn foil, MnO_2, Mn_2O_3.

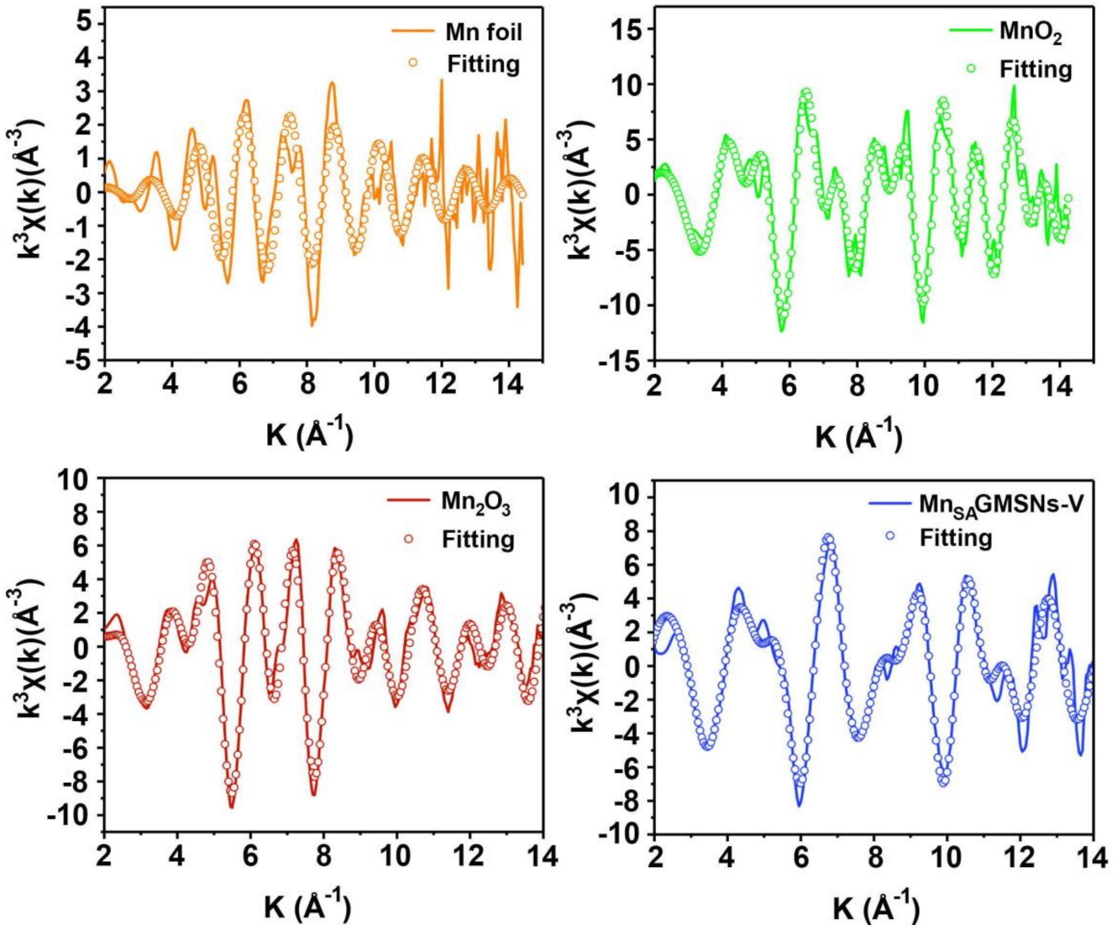

Figure S11. $K^3\chi(k)$ space spectra fitting curve of Mn foil, MnO_2, Mn_2O_3 and Mn_{SA}GMSNs-V.

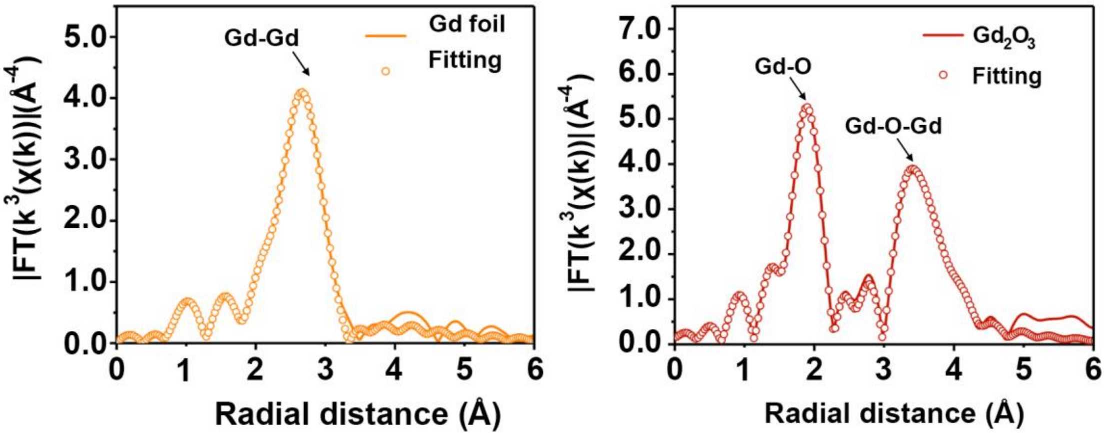

Figure S12. FT k^3-weighted EXAFS fitting curves at R space of Gd foil and Gd_2O_3.

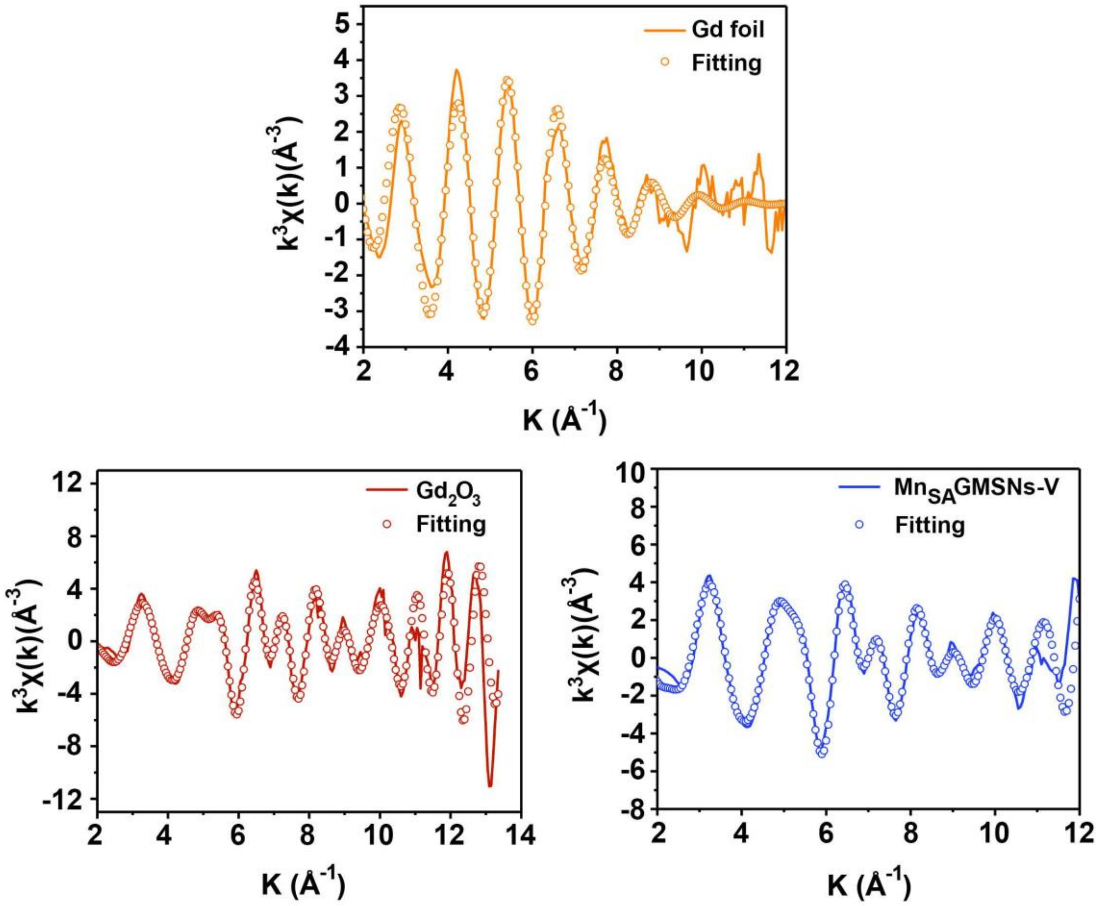

Figure S13. $K^3\chi(k)$ space spectra fitting curve of Gd foil, Gd_2O_3 and Mn_{SA}GMSNs-V.

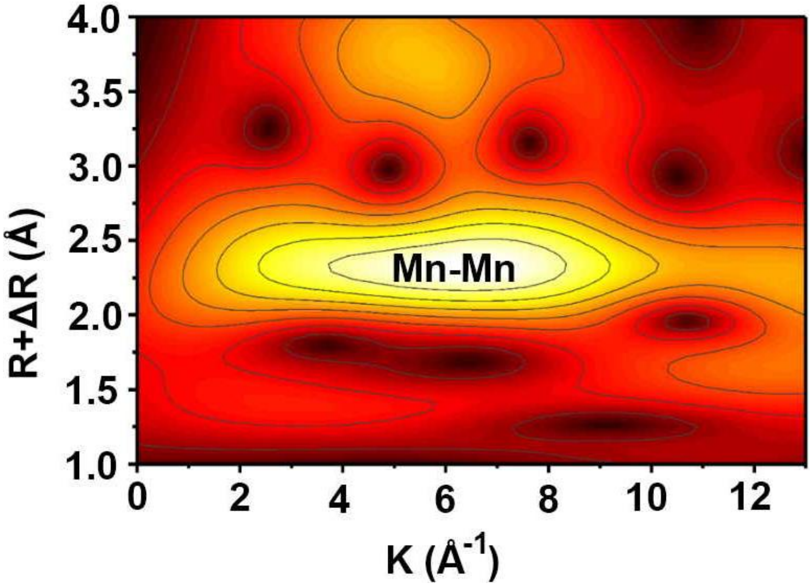

Figure S14. WT-EXAFS contour maps of Mn foil.

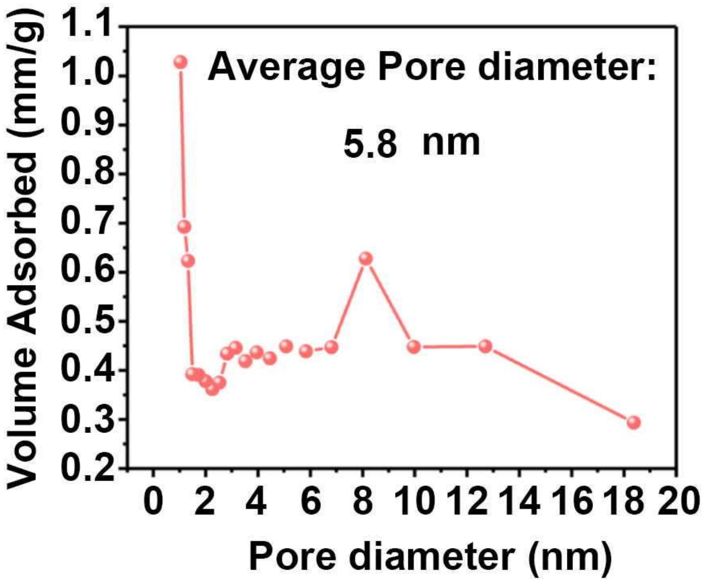

Figure S15. The corresponding pore-size distribution of Mn_{SA}GMSNs-V.

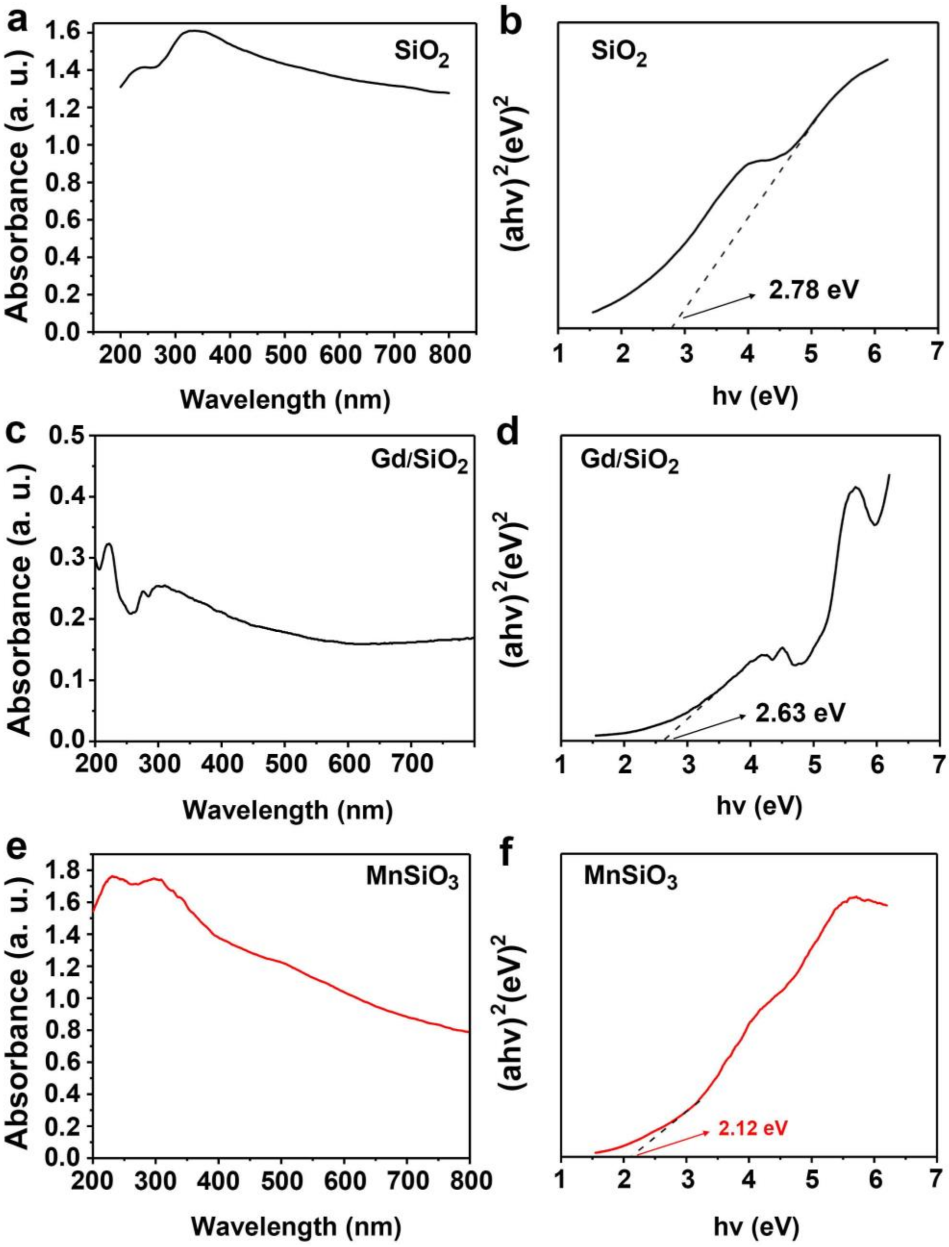

Figure S16. UV-vis diffuse absorbance spectra and the corresponding plots of $(\alpha h\nu)^{n/2}$ for pure SiO_2 (a, b), Gd_2O_3 (c, d) and $MnSiO_3$ (e, f).

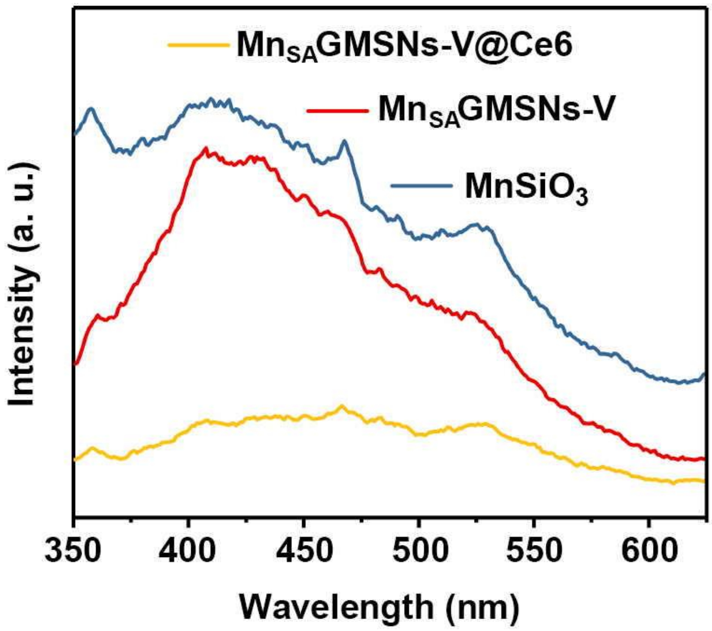

Figure S17. Photoluminescence spectra of $MnSiO_3$, Mn_{SA}GMSNs-V and Mn_{SA}GMSNs-V@Ce6.

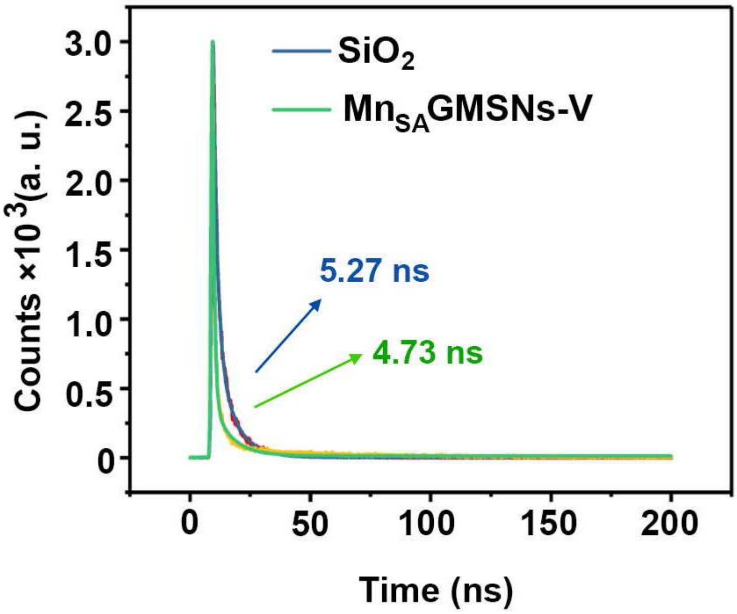

Figure S18. Time-resolved transient PL decay for pure SiO_2 and Mn_{SA}GMSNs-V.

$$\tau = \frac{A_1\tau_1^2 + A_2\tau_2^2}{A_1\tau_1 + A_2\tau_2}$$

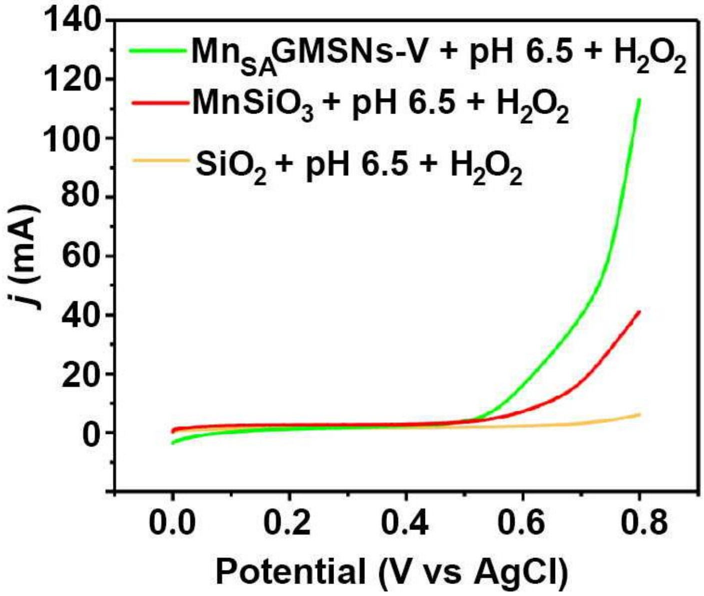

Figure S19. LSV curves of SiO_2, $MnSiO_3$ and Mn$_{SA}$GMSNs-V.

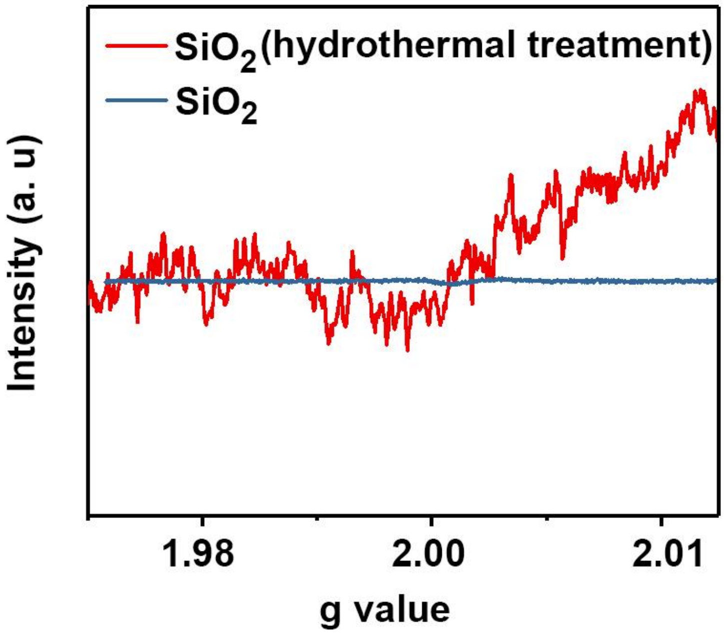

Figure S20. The ESR spectra of pure SiO_2 and SiO_2 after hydrothermal treatment.

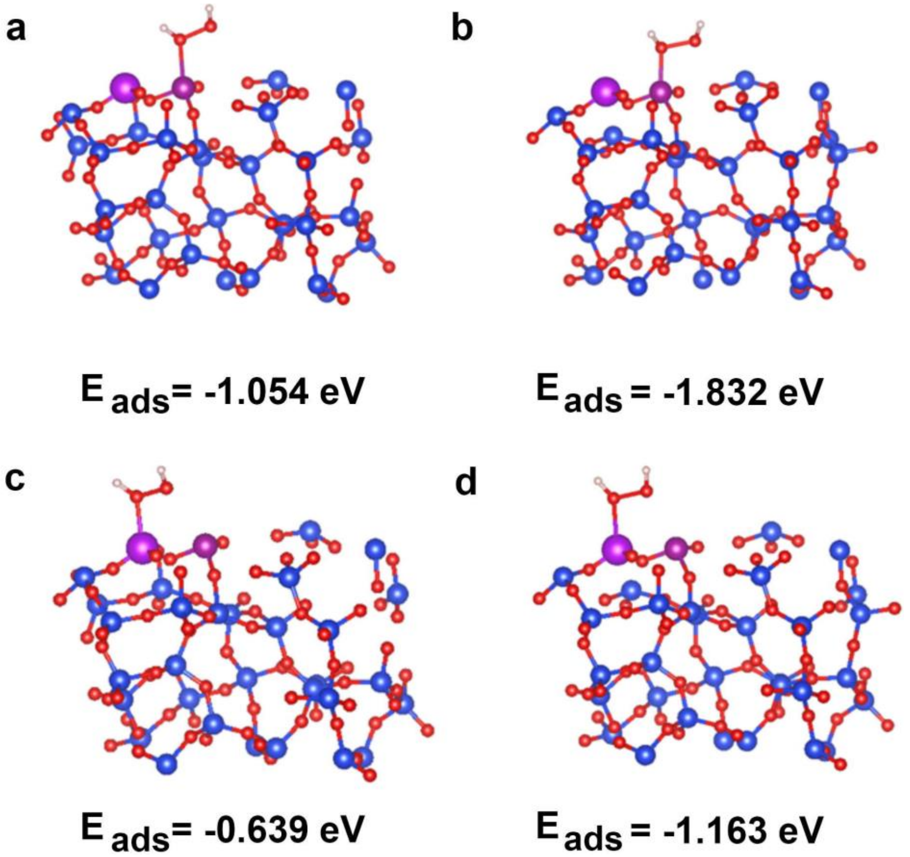

Figure S21. Adsorption structure and free energy of H_2O_2 on Mn (a,b) and Gd (c,d) sites (a,c represent Mn_{SA}GMSNs without oxygen vacancy and b,d represent Mn_{SA}GMSNs-V with oxygen vacancy).

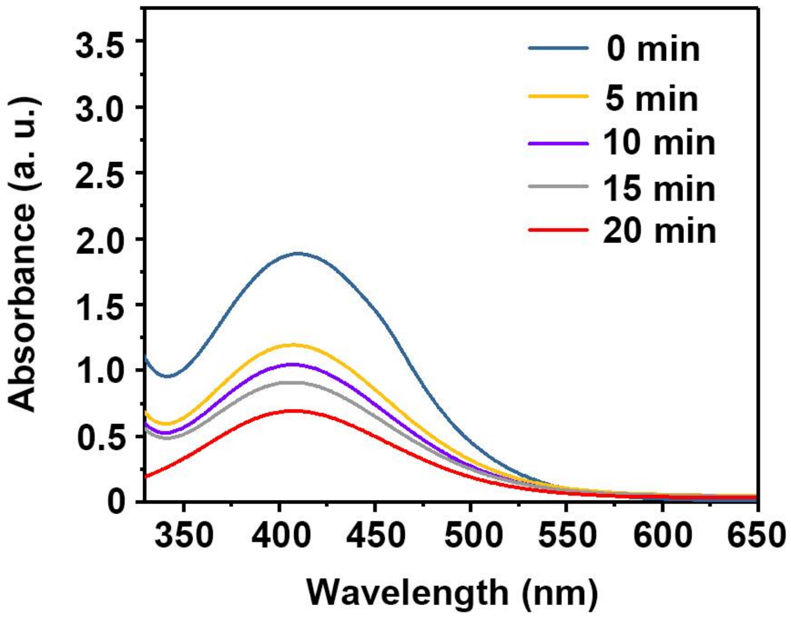

Figure S22. The changes in the color of the H_2O_2 solution treated with PMn_{SA}GMSNs-V under different times.

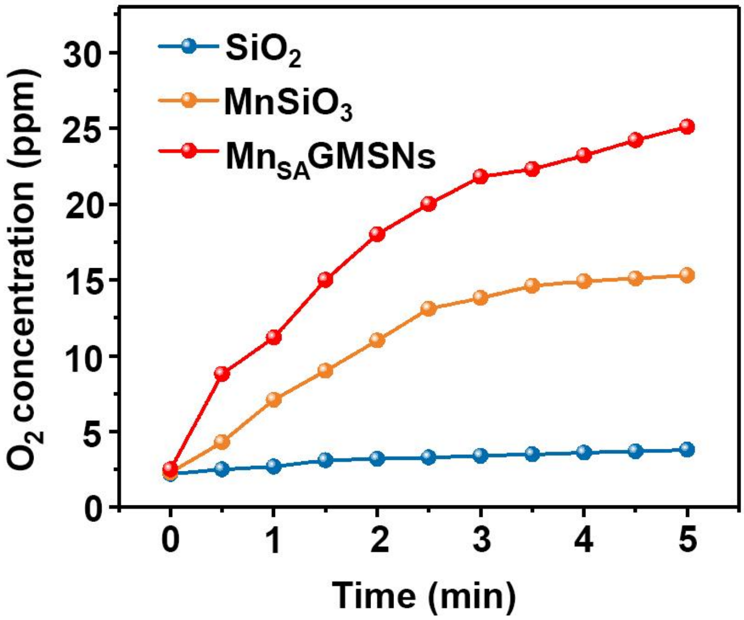

Figure S23. The O_2 generation of different samples with H_2O_2 (1 mL).

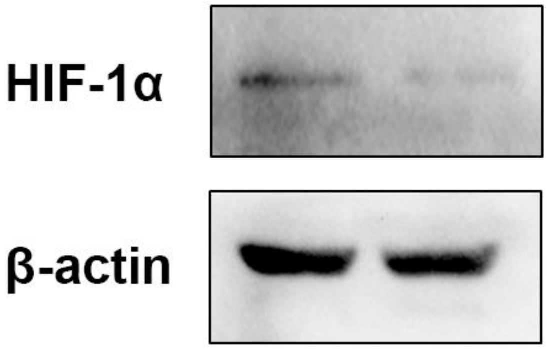

Figure S24. Western blots of HIF-1α expression in HeLa cells treatedwith $PMn_{SA}GMSNs$-V@Ce6.

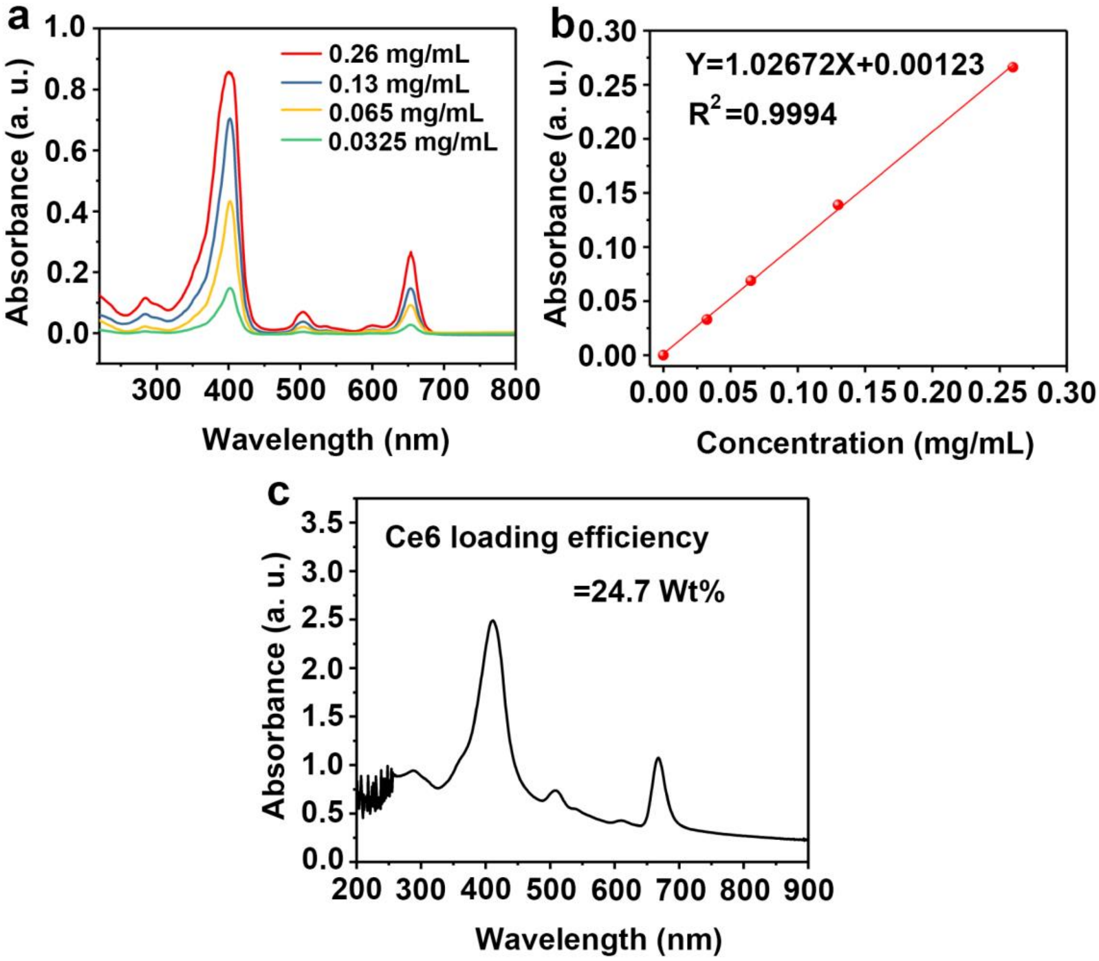

Figure S25. UV-Vis absorbance spectra of Ce6 with different concentrations (a), the standard curve for Ce6 (b), and the Ce6 loading efficiency in Mn_{SA}GMSNs-V@Ce6 (c).

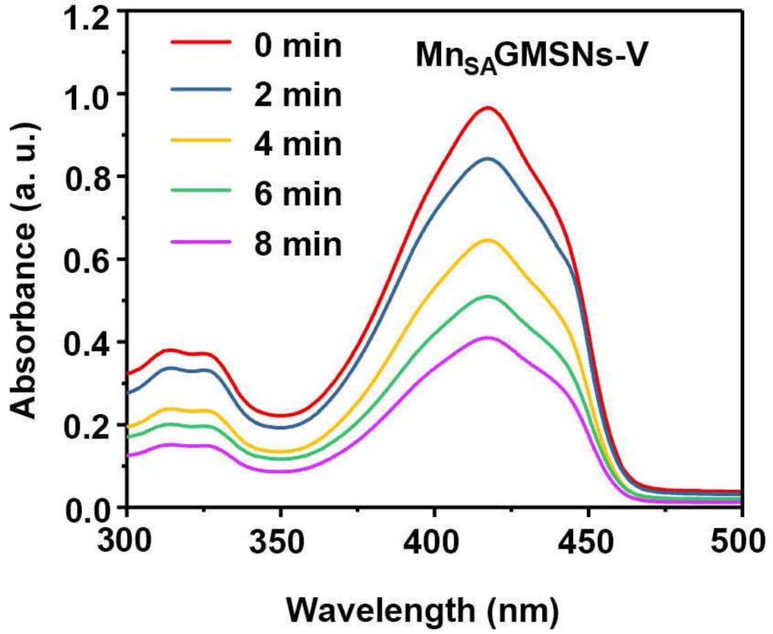

Figure S26. Time-dependent absorption spectra of DPBF in the presence of Mn$_{SA}$GMSNs-V under 650 nm laser irradiation.

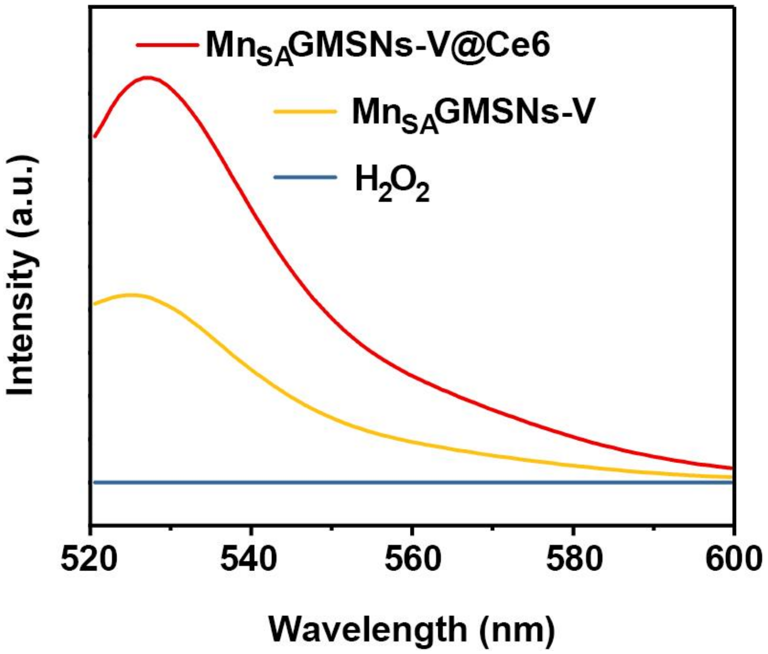

Figure S27. The fluorescence intensity of SOSG in presence of the H_2O_2, Mn$_{SA}$GMSNs-V and Mn$_{SA}$GMSNs-V@Ce6 under 650 nm laser irradiation, respectively.

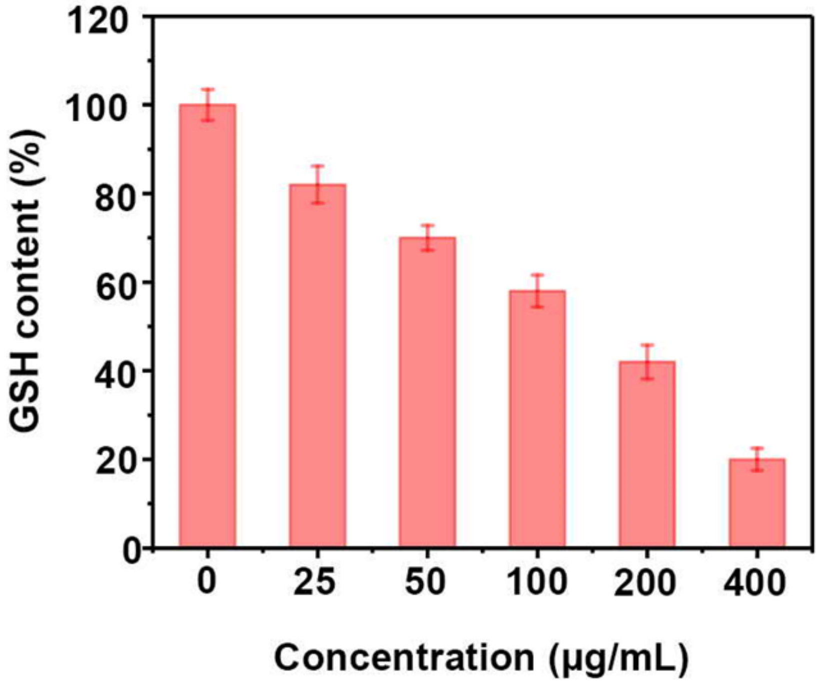

Figure S28. GSH degradation rate in HeLa cells after treatment with different concentrations of Mn_{SA}GMSNs-V.

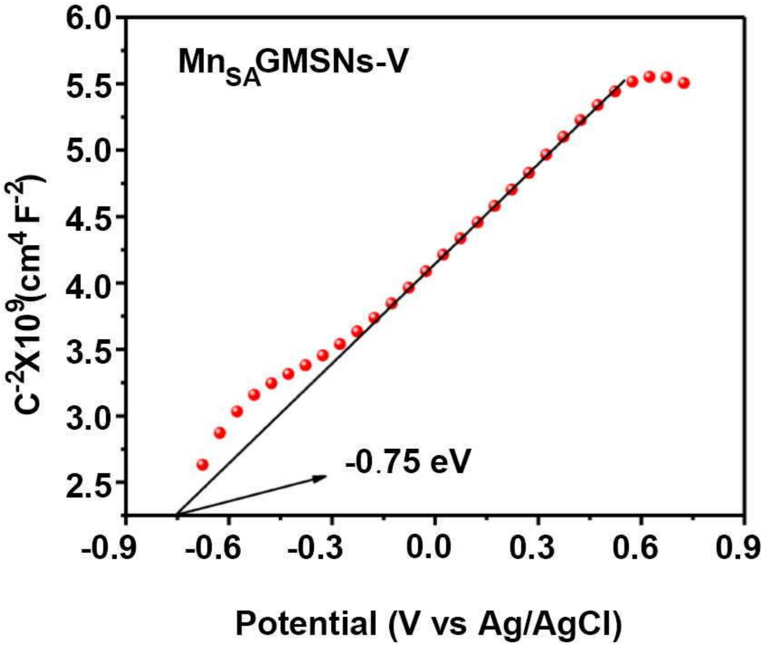

Figure S29. Mott-Schottky plots of Mn$_{SA}$GMSNs-V.

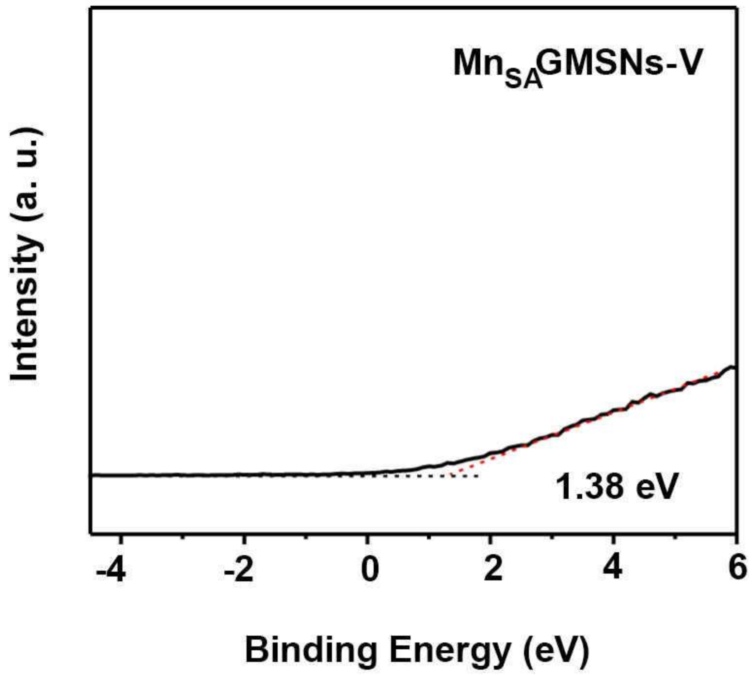

Figure S30. XPS valence band spectrum of Mn_{SA}GMSNs-V.

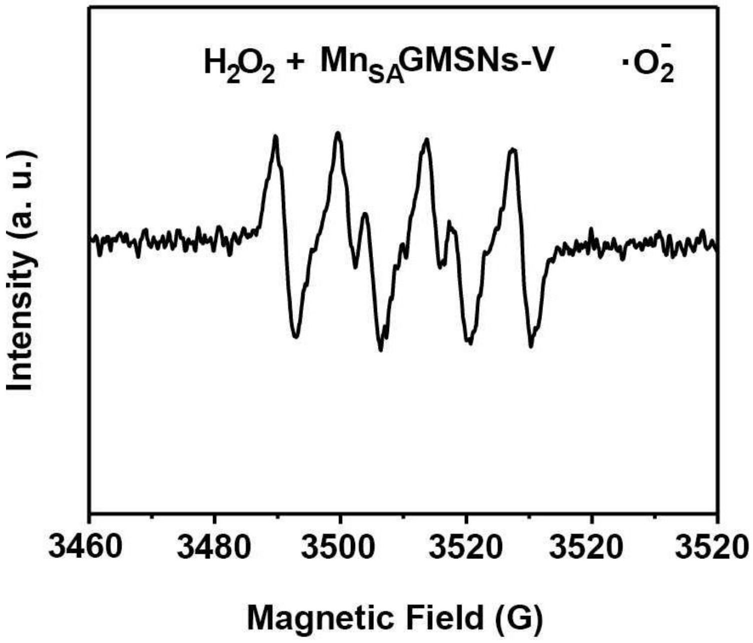

Figure S31. ESR spectrum of $\bullet O_2^-$ for Mn_{SA}GMSNs-V in the presence of H_2O_2 only.

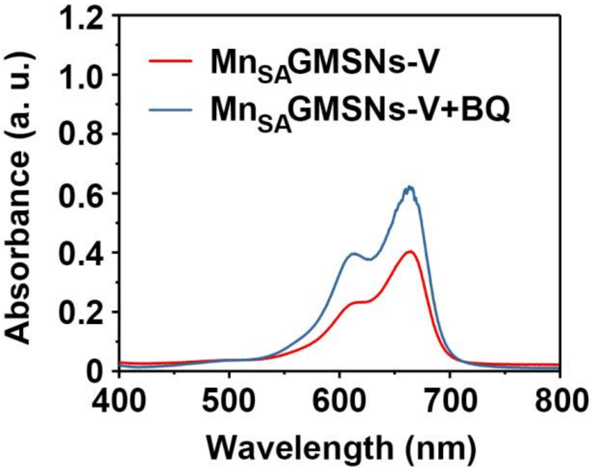

Figure S32. MB degradation process in the presence of BQ and the corresponding to the absorbance of MB at different times.

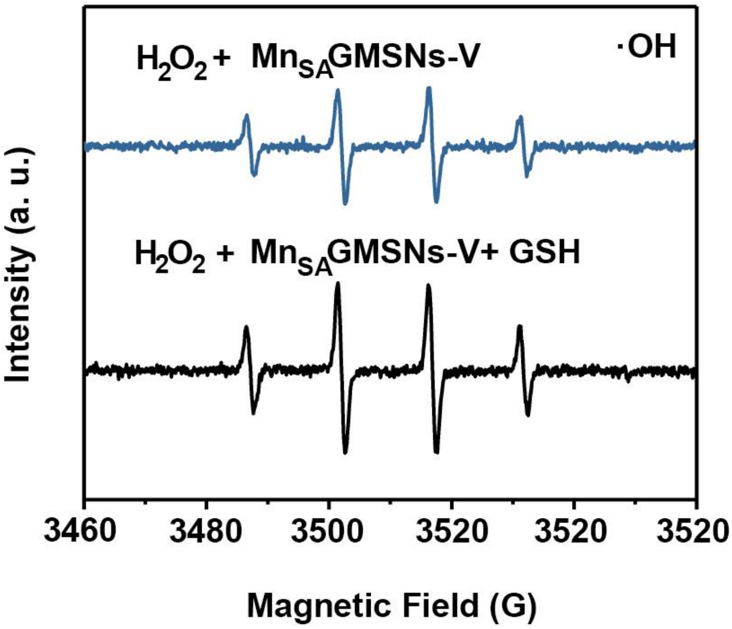

Figure S33. ESR spectra of •OH for Mn_{SA}GMSNs-V in the presence or absence of GSH, respectively.

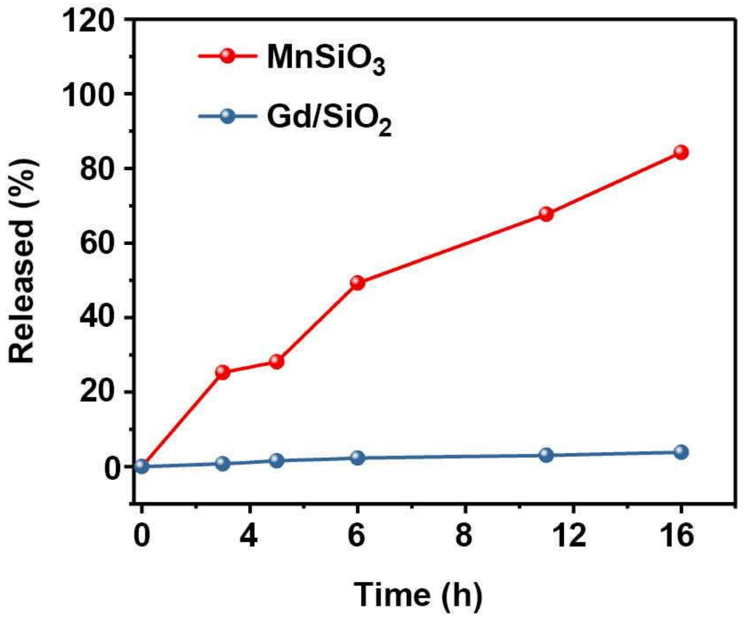

Figure S34. Accumulated releasing Mn and Gd elements from $MnSiO_3$ (1 mg mL^{-1}) and Gd/SiO_2 (1 mg mL^{-1}) with different treatments, respectively.

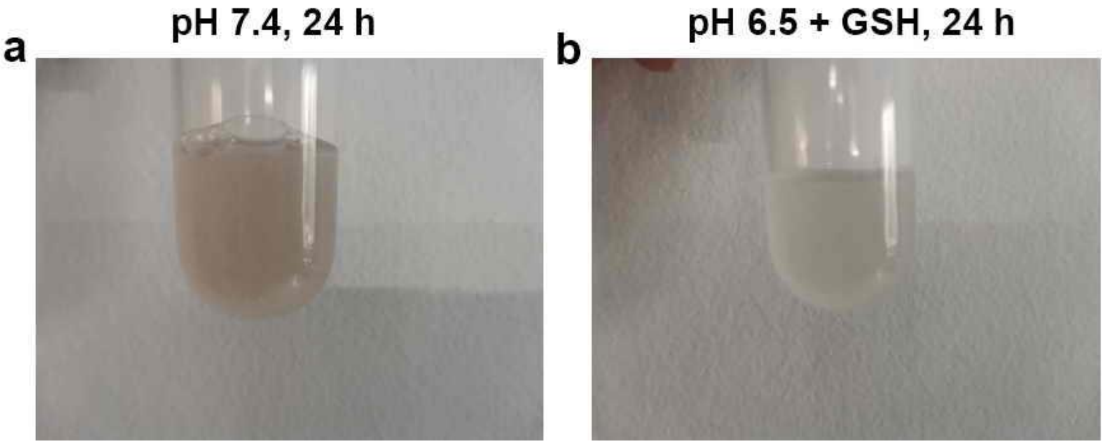

Figure S35. The pictures of Mn_{SA}GMSNs-V treated with solutions of pH 7.4 (a) and pH 6.5 plus GSH (b) for 24 h.

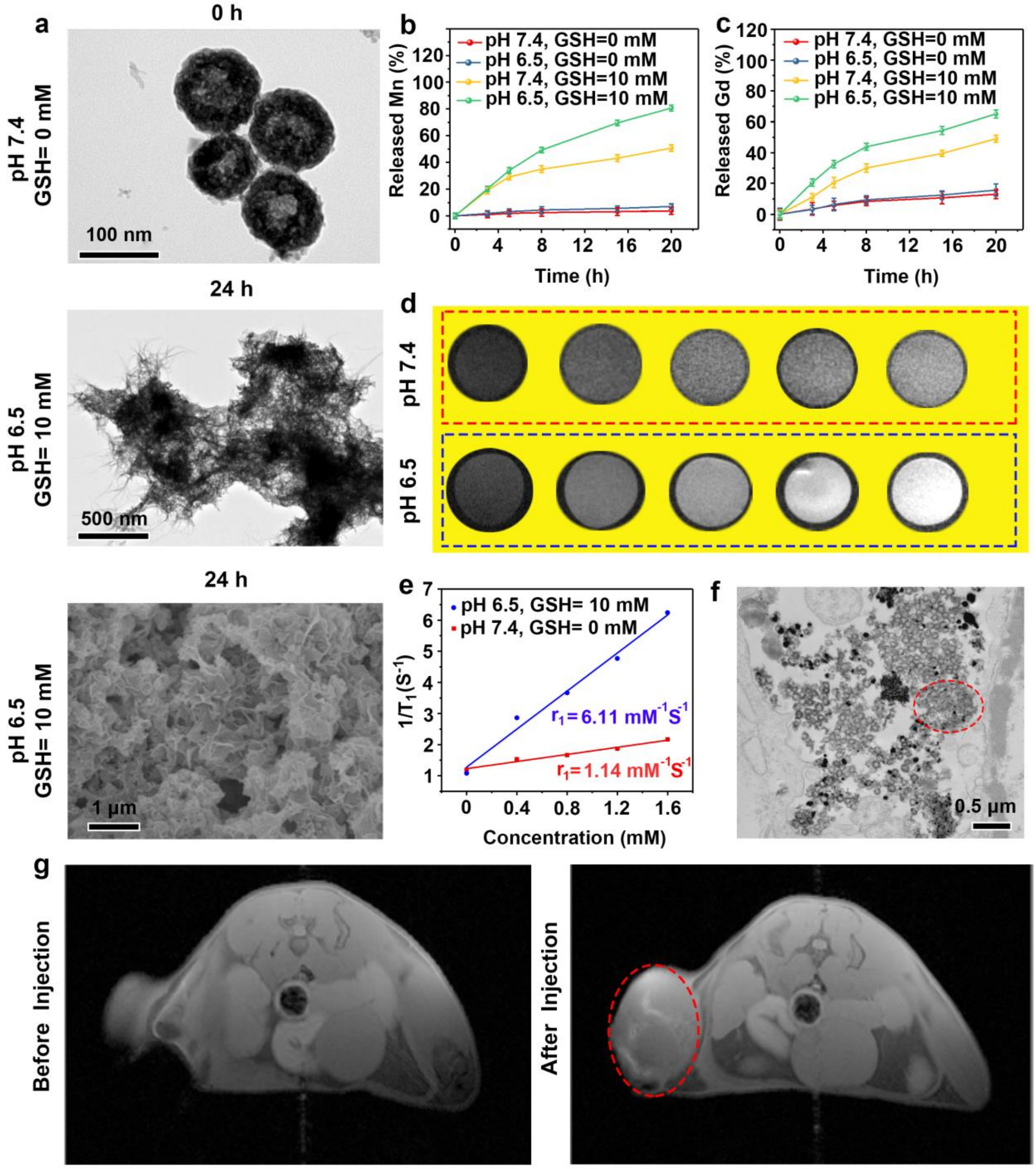

Figure S36. TEM and SEM images of Mn_{SA}GMSNs-V after different treatments for various periods of time (a). Accumulated releasing Mn (b) and Gd (c) elements from Mn_{SA}GMSNs-V (1 mg mL^{-1}) with different treatments. *In vitro* MRI of Mn_{SA}GMSNs-V (d) with different treatments and the corresponding r_1 value (e). Bio-TEM images of HeLa cells incubated with Mn_{SA}GMSNs-V for 0.5 h (f). *In vivo* T_1-weighted MRI of tumor-bearing mice before and after injection of Mn_{SA}GMSNs-V (g).

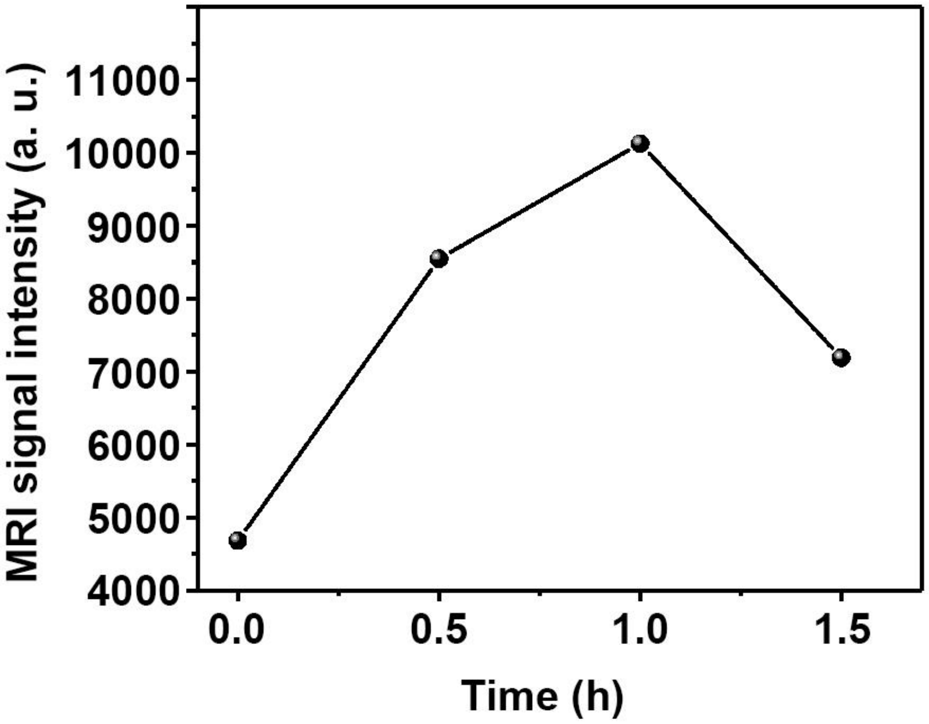

Figure S37. The corresponding MRI signal intensity in tumor tissue at different time after injection of of $PMn_{SA}GMSNs$-V.

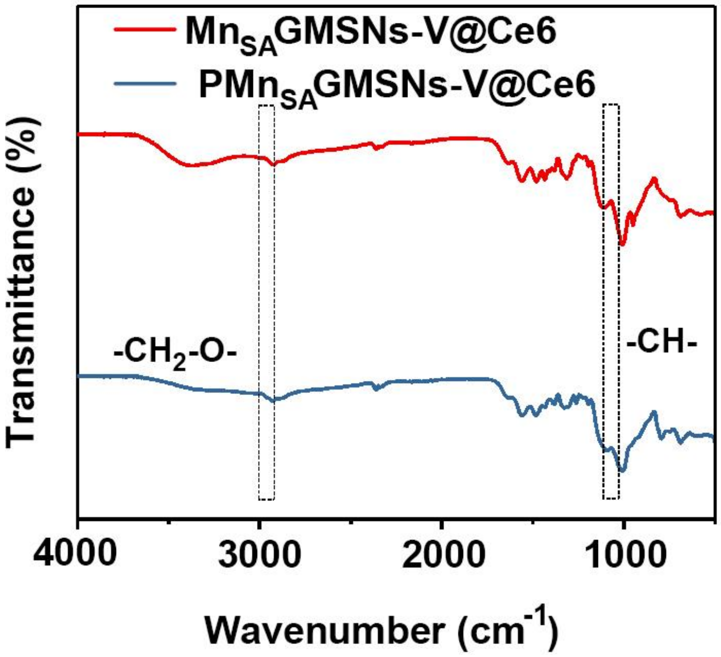

Figure S38. The fourier transform infrared (FTIR) spectra of Mn$_{SA}$GMSNs-V and PMn$_{SA}$GMSNs-V.

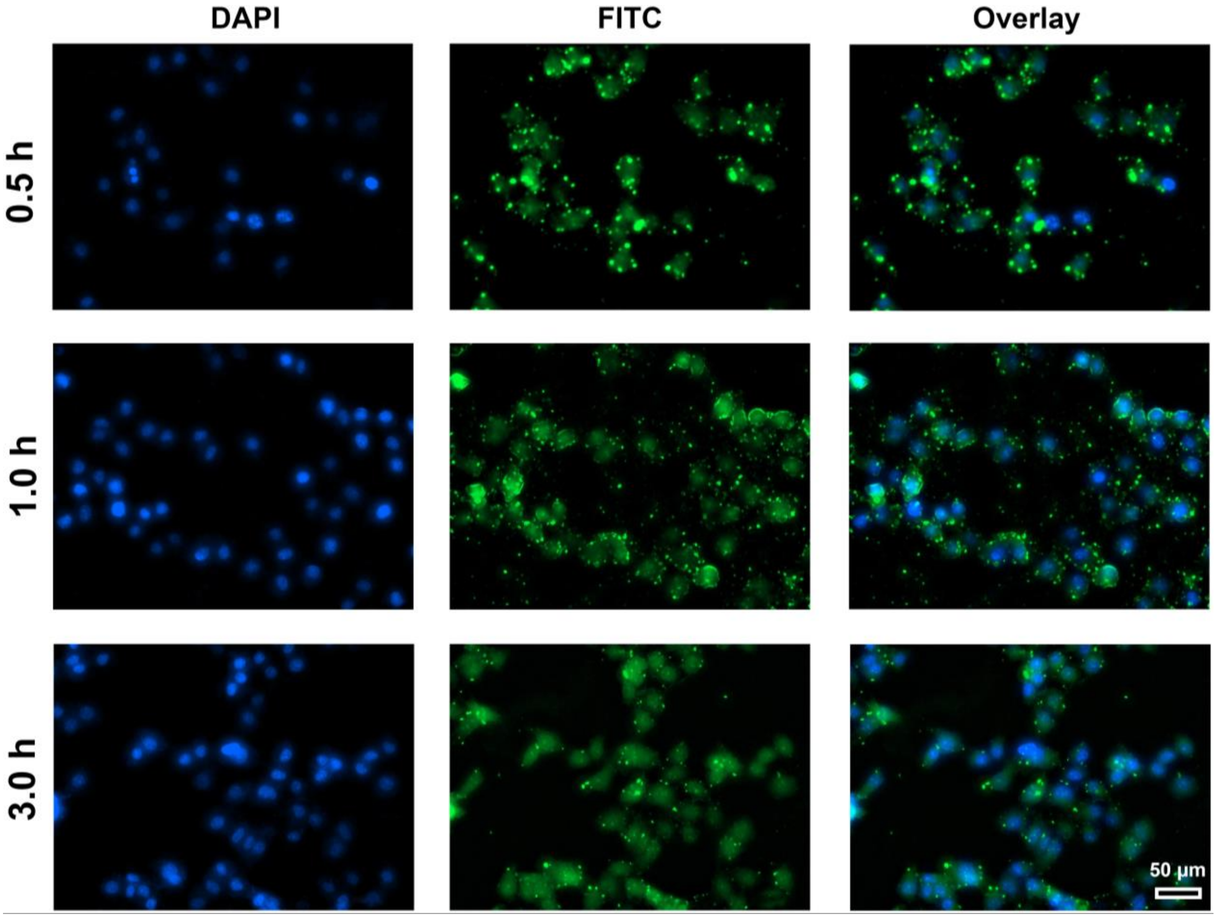

Figure S39. CLSM images of HeLa cells incubated with DAPI and FITC-decorated $PMn_{SA}GMSNs$-V@Ce6 at different times.

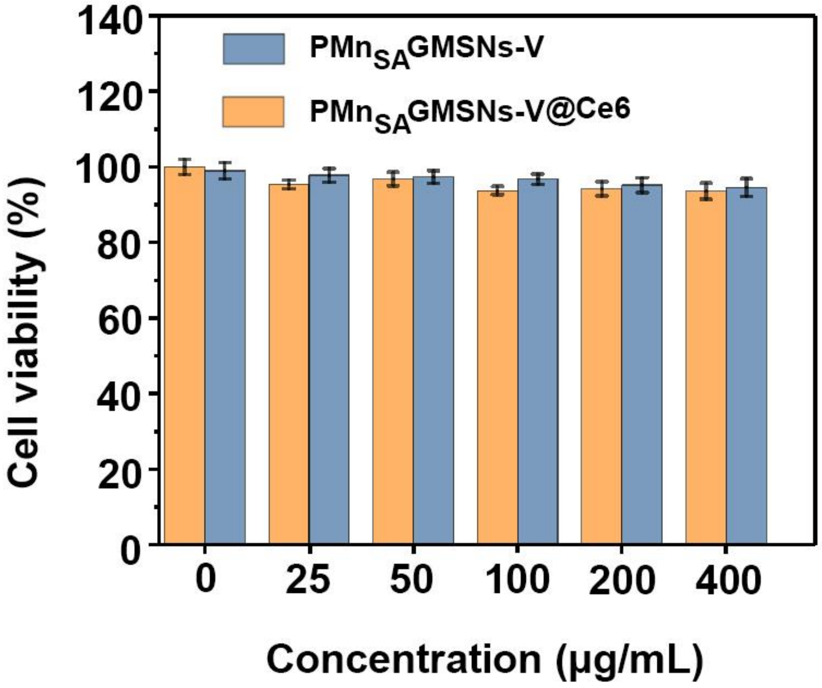

Figure S40. Viabilities of L929 cells treated with $PMn_{SA}GMSNs$-V and $PMn_{SA}GMSNs$-V@Ce6.

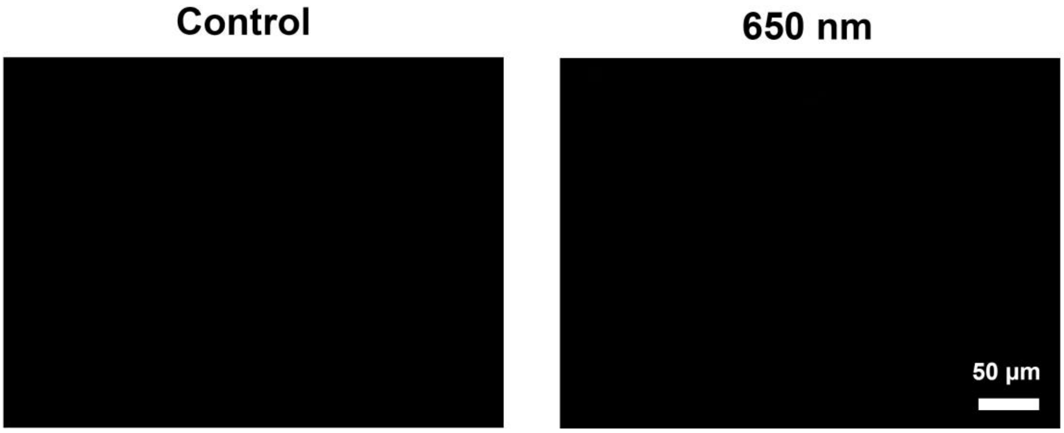

Figure S41. Intracellular ROS detections using DCFH-DA as probe in the control and 650 nm laser irradiated groups.

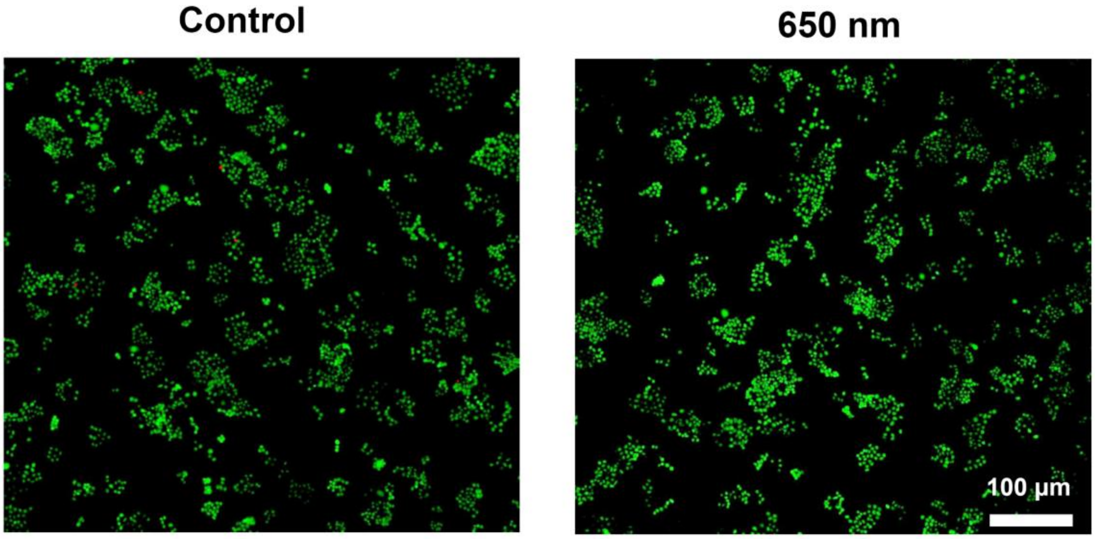

Figure S42. CLSM images dyed with AM and PI of HeLa cells in the control and 650 nm laser irradiated groups.

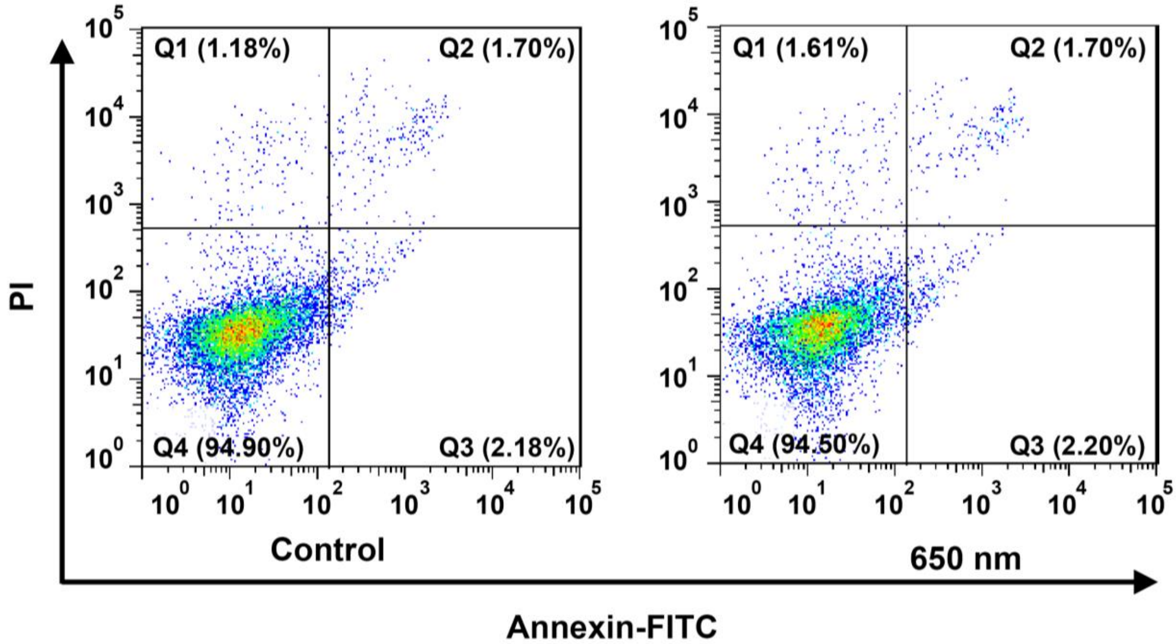

Figure S43. Apoptosis of HeLa cells detected by flow-cytometry in the groups of control and 650 nm.

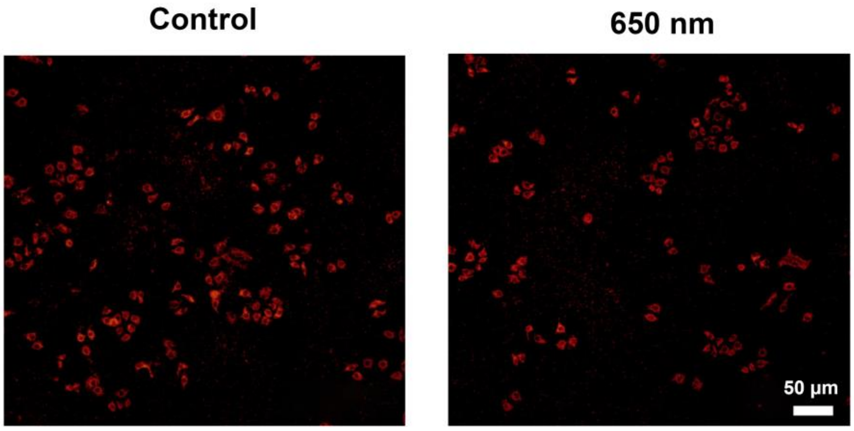

Figure S44. CLSM images of HeLa cells stained by JC-1 after incubation with control and 650 nm.

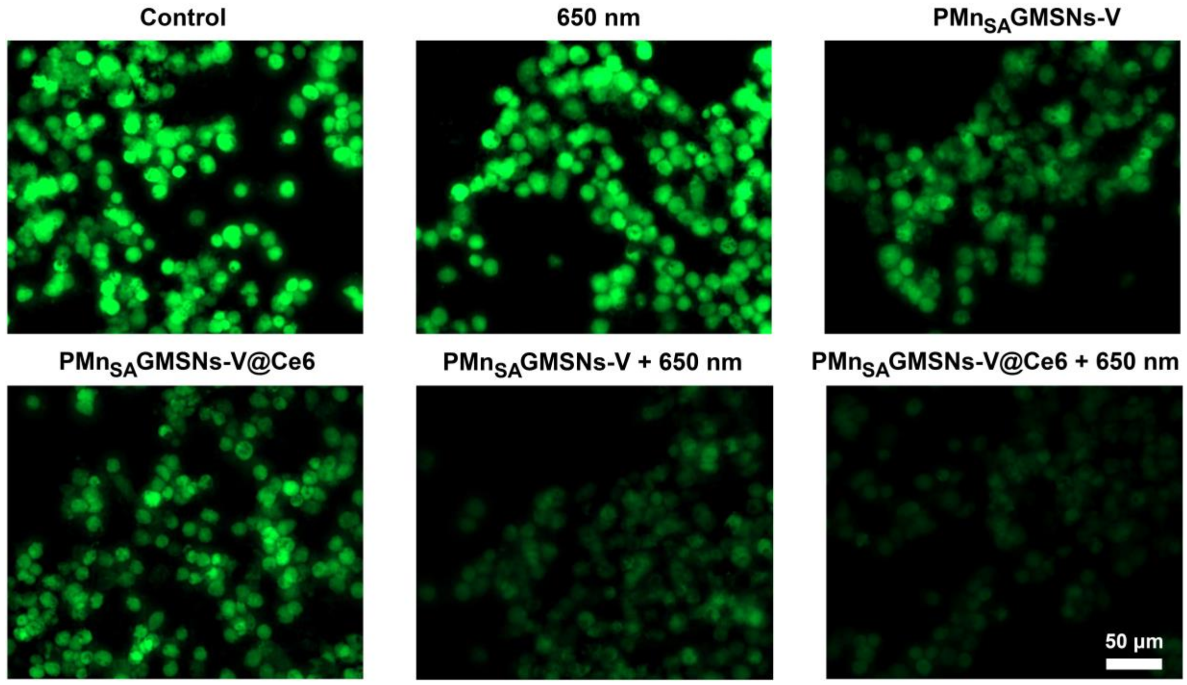

Figure S45. CLSM images of the intratumoral GSH levels by glutathione detection reagent.

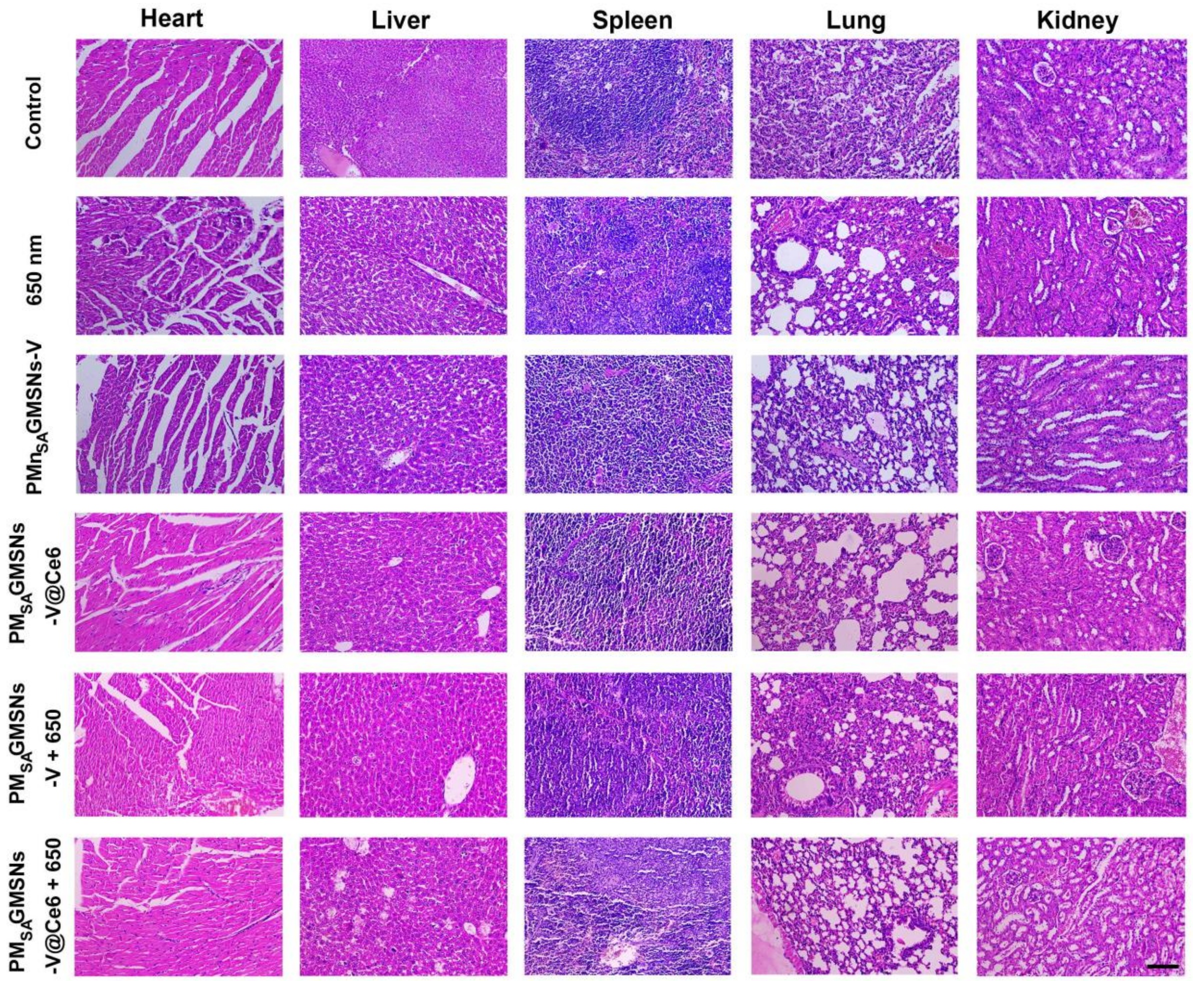

Figure S46. H&E stained images of heart, liver, spleen, lung, and kidney in control, 650 nm, $PMn_{SA}GMSNs$-V, $PMn_{SA}GMSNs$-V@Ce6, $PMn_{SA}GMSNs$- plus 650 nm and $PMn_{SA}GMSNs$-V@Ce6 plus 650 nm (scale bar: 100 μm).

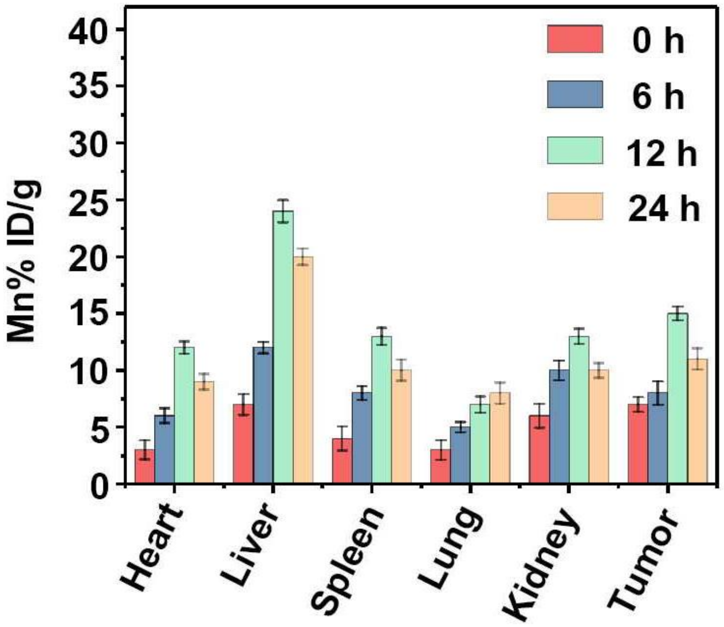

Figure S47. Biodistribution of PMn$_{SA}$GMSNs-V@Ce6 *in vivo* at 0, 6, 12 and 24 h.

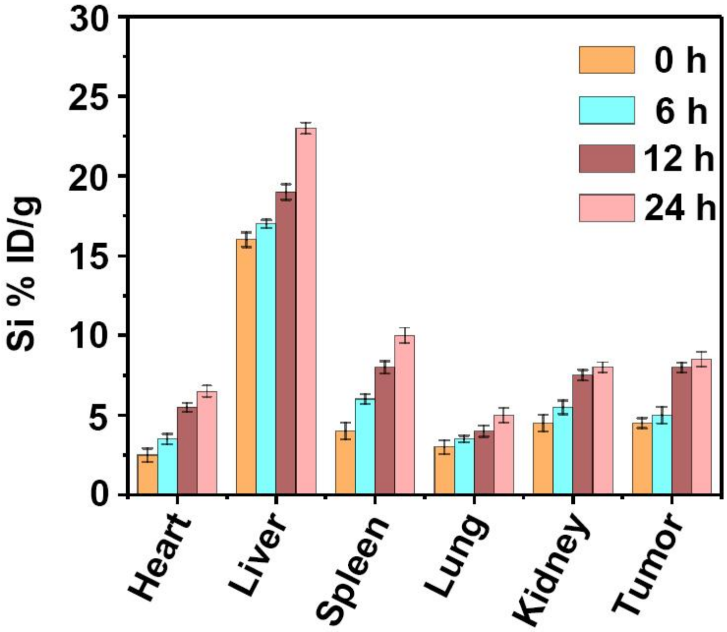

Figure S48. Biodistribution of Si element *in vivo* at 0, 6, 12 and 24 h.

Table S1. The contents of Gd and Mn on Mn_{SA}GMSNs tested by ICP-OES.

Elements	Gd	Mn
Wt (%)	34.90	17.00

Table S2. EXAFS fitting parameters at the Mn K-edge and Gd L_3-edge for various samples.

Sample	Shell	CN^a	R(Å)^b	σ^2(Å^2)^c	ΔE_0(eV)^d	R factor
			Mn K-edge (S_0^2 = 0.837)			
Mn foil	Mn-Mn	12*	2.684±0.001	0.0067±0.0025	8.6±3.4	0.0072
Mn_2O_3	Mn-O	6.0±0.3	1.939±0.010	0.0039±0.0008	-7.8±2.6	0.0045
	Mn-Mn	12.3±0.5	3.121±0.012	0.0057±0.0011		
MnO_2	Mn-O	6.0±0.3	1.927±0.008	0.0028±0.0008	-2.6±2.1	0.0089
	Mn-Mn	4.2±0.5	2.928±0.024	0.0063±0.0010		
	Mn-Mn	6.3±0.6	3.476±0.012			
Mn_{SA}GMSNs-V	Mn-O	3.7±0.2	1.947±0.004	0.0018±0.0004	3.9±1.1	0.0027
	Mn-Si	5.5±0.7	2.965±0.008	0.0037±0.0008		
	Mn-Gd	1.6±0.8	3.227±0.025	0.0081±0.0026		
			Gd L_3-edge (S_0^2 = 0.920)			
Gd foil	Gd-Gd	6.0*	2.706±0.035	0.0132±0.0035	-5.9±2.5	0.0070
	Gd-Gd	6.0*	2.976±0.028			
Gd_2O_3	Gd-O	6.2±0.3	2.315±0.010	0.0070±0.0012	2.1±1.3	0.0097
	Gd-Gd	6.1±0.5	3.584±0.013	0.0067±0.0014		
	Gd-Gd	5.8±0.5	4.086±0.011			
Mn_{SA}GMSNs-V	Gd-O	5.5±0.6	2.313±0.010	0.0117±0.0016	1.3±1.2	0.0079
	Gd-Gd	2.7±0.6	3.569±0.020	0.0107±0.0040	3.5±3.8	
	Gd-Gd	2.8±0.3	3.887±0.017			

^{a}*CN*, coordination number; ^{b}*R*, the distance to the neighboring atom; $^c\sigma^2$, the mean square relative displacement (MSRD); $^d\Delta E_0$, inner potential correction; *R* factor indicates the goodness of the fit. S_0^2 was fixed to 0.837, 0.734 and 0.920, according to the experimental EXAFS fit of Mn foil and Gd foil by fixing *CN* as the known crystallographic value. * This value was fixed during EXAFS fitting, based on the known structure of Mn and Gd. Fitting range: $3.0 \le k$ (/Å) ≤ 11.5 and $1.8 \le R$ (Å) ≤ 3.0 (Mn foil and Gd foil); $3.0 \le k$ (/Å) ≤ 13.5 and $1.1 \le R$ (Å) ≤ 3.5 (Mn_{SA}GMSNs-V); $3.0 \le k$ (/Å) ≤ 12.0 and $1.0 \le R$ (Å) ≤ 4.5 (Mn_{SA}GMSNs-V). A reasonable range of EXAFS fitting parameters: $0.700 < S_0^2 < 1.000$; $CN > 0$; $\sigma^2 > 0$ Å^2; $|\Delta E_0| < 10$ eV; *R* factor < 0.02.

References

[1] a) J. F. l. G. Kresse, *Phys. Rev. B* **1996**, *54*, 11169; b) K. B. John P. Perdew, Matthias Ernzerhof, *Phys. Rev. Lett.* **1996**, *77*, 3865.

[2] G. Kresse, & Joubert, D., *Phys. Rev. B* **1999**, *59*, 1758.

[3] a) S. Grimme, J. Antony, S. Ehrlich, H. Krieg, *J. Chem. Phys.* **2010**, *132*, 154104; b) P. E. Blochl, *Phys. Rev. B* **1994**, *50*, 17953.

[4] G. Henkelman, B. P. Uberuaga, H. Jónsson, *J. Phys. Chem. C* **2000**, *113*, 9901.
